# Protoporphyrin IX iron(II) revisited. An overview of the Mössbauer spectroscopic parameters of low-spin porphyrin iron(II) complexes

**DOI:** 10.1007/s00775-024-02075-9

**Published:** 2024-10-10

**Authors:** Jack Silver, Daniel den Engelsen, Golzar al-Jaff, Jehad A. Taies, Michael T. Wilson, George R. Fern

**Affiliations:** 1 Department of Chemical Engineering, Wolfson Centre for Sustainable Materials Processing and Development, Brunel University of London, Kingston Lane, Uxbridge, Middlesex UB8 3PH UK; 2 School of Life Sciences, University of Essex, Wivenhoe Park, Colchester, Essex CO4 3SQ UK; 3 Department of Chemistry, College of Education, Salahaddin University, Erbil, Iraq; 4 Depatment of Chemistry, College of Education for Pure Science, Ramedi, Iraq

**Keywords:** Iron porphyrins, Mössbauer spectroscopy, Myoglobin, Haemoglobin, Carbon monoxide, Nitrogenous bases

## Abstract

Mössbauer parameters of low-spin six-coordinate [Fe(II)(Por)L_2_] complexes (where Por is a synthetic porphyrin; L is a nitrogenous aliphatic, an aromatic base or a heterocyclic ligand, a P-bonding ligand, CO or CN) and low-spin [Fe(Por)LX] complexes (where L and X are different ligands) are reported. A known point charge calculation approach was extended to investigate how the axial ligands and the four porphyrinato-N atoms generate the observed quadrupole splittings (*ΔE*_Q_) for the complexes. Partial quadrupole splitting (p.q.s.) and partial chemical shifts (p.c.s.) values were derived for all the axial ligands, and porphyrins reported in the literature. The values for each porphyrin are different emphasising the importance/uniqueness of the [Fe(PPIX)] moiety, (which is ubiquitous in nature). This new analysis enabled the construction of figures relating p.c.s and p.q.s values. The relationships presented in the figures indicates that strong field ligands such as CO can, and do change the sign of the electric field gradient in the [Fe(II)(Por)L_2_] complexes. The limiting p.q.s. value a ligand can have and still form a six-coordinate low-spin [Fe(II)(Por)L_2_] complex is established. It is shown that the control the porphyrin ligands exert on the low-spin Fe(II) atom limits its bonding to a defined range of axial ligands; outside this range the spin state of the iron is unstable and five-coordinate high-spin complexes are favoured. Amongst many conclusions, it was found that oxygen cannot form a stable low-spin [Fe(II)(Por)L(O_2_)] complex and that oxy-haemoglobin is best described as an [Fe(III)(Por)L(O_2_^−^)] complex, the iron is ferric bound to the superoxide molecule.

**Graphical abstract:**

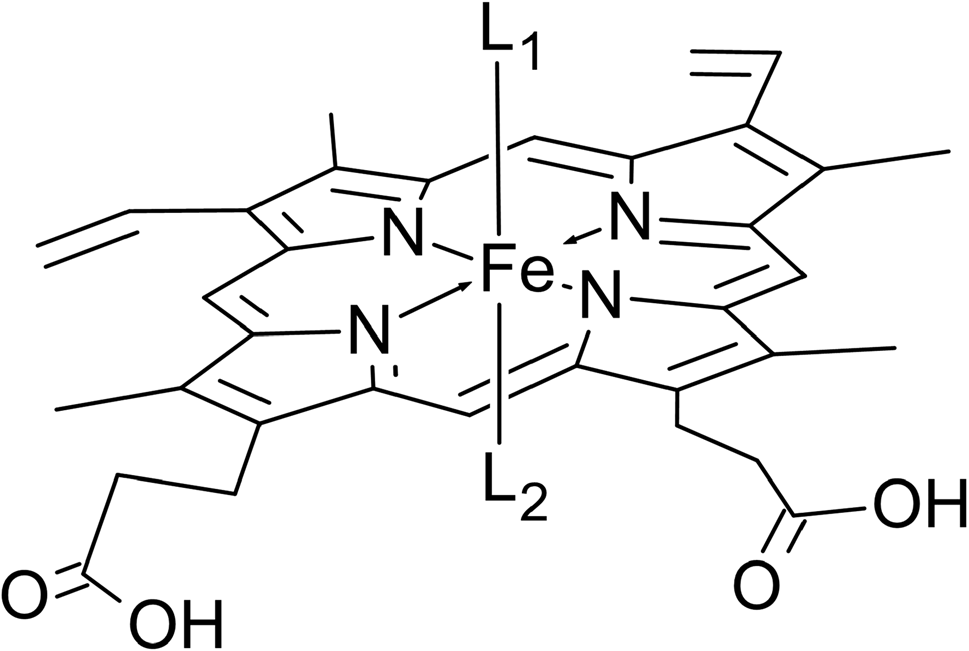

## Introduction

Iron protoporphyrin IX, ([Fe(PPIX)]) and related naturally occurring haems (iron porphyrin macrocycles) form the active centres in a wide range of biological molecules that are all vital for living organisms. These haem groups perform crucial functional roles such as in oxygen transport (haemoglobin (Hb)) and storage (myoglobin (Mb)), electron transport (the cytochromes) and in the elimination of toxic and unwanted compounds (cytochrome P450) [1–5]. The chemical properties of the iron in the haem are manipulated/controlled both by the porphyrin and by the nature of the axial ligands present [4–6]. The manner in which the immediate environment of the metal is influenced by electron delocalization on the macrocycle and the nature and binding properties of the axial ligands in haem complexes has aroused continuous discussion for more than 55 years [1–5, 7–14]. Many structures/molecules containing natural and synthetic haems have been studied to obtain insight into porphyrin metal-bonding interactions and how axial ligands may control and/or modify this bonding [15–20].

Over the last four decades we have carried out extensive studies on [Fe(PPIX)], using it as a model compound for haem proteins found in natural enzymes [21–46]. In our studies on [Fe(PPIX)] chemistry we have used Mössbauer and electronic spectroscopies [21–46], to both probe and monitor changes at the iron and the porphyrin. We have been able to demonstrate that by selecting the axial ligand, the spin states of both iron(II) and iron(III) and the geometry of the complexes can be varied/controlled. In complementary studies to this work we have applied the understanding gained; (1) to haem peptides derived from cytochrome c [47–49]; (2) to the role of [Fe(PPIX)] in porphyromonas gingivalis [44, 50–55] and other oral anaerobes [56, 57]; and (3) to haem-antimalarial complexes of pharmacological interest [58, 59]. In further papers, we have reported the properties of none naturally occurring iron porphyrins and compared these to [Fe(PPIX)] [60–71]. Although, all these macrocycles had the same inner core structure, we showed they manifested significantly different chemistry to [Fe(PPIX)] [60–71].

We have previously reported studies on a wide range of nitrogenous ligands binding to [Fe(II)(PPIX)] [38–40]. These studies covered the binding of [Fe(II)(PPIX)] to pyridine, substituted pyridines, imidazole, aliphatic amines, piperidine and a range of heterocyclic five- and six-membered ring ligands [38–40]. The results were compared to previous literature on binding studies of pyridines and imidazoles to haems in non-aqueous systems. In doing so we summarised the many different factors that affect such binding and were able to gain further insight to how variation in the bonding properties of such ligands can affect the [Fe(II)(PPIX)] entity. Stability constants were calculated from electronic absorption spectra (in the visible and near UV range) and Mössbauer spectra were obtained from frozen solutions of the complexes. We also studied a small range of sterically hindered nitrogenous ligands to gain understanding on how steric effects may modify bonding. Although such studies yielded much new information we realised when writing the papers that there was much more knowledge that could be extracted from comparisons of the Mössbauer parameters, both to a wide range of other low-spin[Fe(II)(PPIX)L_2_] complexes as well as to those of the equivalent complexes with other low-spin[Fe(II)(Por)L_2_] complexes with Por such as tetra(phenyl)porphyrin (TPP), tetramesitylporphyrin (TMP), and octaethylporphyrin (OEP). Such a study would allow a comparison to complexes, where in some cases the crystal structures are known, so further insights into the bonding in low-spin [Fe(II)(PPIX)L_2_] could be inferred.

Amongst the many studies on low-spin six-coordinate porphyrinato-iron(II), where the axial ligands are nitrogenous aromatic or aliphatic ligands, have been those that contain crystallographic and/or Mössbauer spectroscopic data [20]. One such study that considered ligand orientation control gave amongst the major conclusions the fact that Mössbauer spectra provide a probe for ligand orientation when structural data may not be available [20]. The same paper presents a discerning overview of the orientations of planar axial ligands in bis-ligated haems of both iron(II) and iron(III). This paper summarises and discusses a rich literature of crystal structures comparing the relative orientation of the two axial planar ligands to each other and to the four nitrogen atoms of the porphyrin core; in addition, it gives the Mössbauer parameters of the same complexes [20]. We believe this paper is a “classic” and has already formed the basis of further work.

Previous workers provided insight into the bonding properties of ligands and low-spin iron(II) compounds by analysing their Mossbauer parameters [72–75]. Such low spin iron(II) complex analysis has utilised p.q.s. values and p.c.s. values. Previously, we have used p.q.s. values to examine a range of bidentate phosphine ligands and have shown that in the main they are stronger σ donors than monodentate phosphine ligands [76]. To date only a handful of papers utilising p.c.s. and p.q.s. values on tetradentate macrocyclic ligands have appeared. The main thrust of the first two concerned synthetic non-porphyrin macrocyclic tetradentate ligands [77, 78], although some brief comments were made on low-spin iron(II)porphyrin complexes. Since 1990 three papers reporting p.q.s. values for low-spin phthalocyanato-iron(II) complexes have been published [79–81], but there have been no further reports on low-spin iron(II)porphyrin complexes. To address this notable deficiency in the literature was a major motive in undertaking this work.

Until our recent work on [Fe(PPIX)(CO)L] complexes [41] there have been few reports of Mössbauer parameters for [Fe(PPIX)(CO)L] complexes [27, 82]. It is well established that affinity between CO and Hb is around 200 times greater than that of oxygen with Hb [2, 3]. To gain further understanding of such CO binding and the mechanistic changes in the bonding around the iron atom is a major aim of this work and our recent extensive studies on [Fe(PPIX)(CO)L] complexes [41] have facilitated much of the data needed for this. These investigations were directed at examining σ- and π-bonding effects as well as steric effects in the bonding of the axial ligands. In view of our many previous papers on protoporphyrin IX and as it is the most widespread porphyrin found in natural proteins, we thought it useful to see how the synthetic porphyrins compared to it. Other reasons for its selection and its limitations have previously been discussed [35–38].

In this paper a wide range of Mössbauer spectroscopic data for [Fe(PPIX)L_2_], [Fe(PPIX)L(CO)] and [Fe(PPIX)(CO)_2_] complexes are studied along with many other low-spin[Fe(II)(Por)L’_2_] complexes (where L’ = a wide range of other axial ligands such as sulphur containing aromatic rings, NO, CN^−^ and many others). Here the aim was to discuss the results of applying an extensive p.c.s. and p.q.s. analysis to both new data (38 Mössbauer spectra) as well as a wide range of low-spin iron(II) porphyrin and iron(II) phthalocyanine complexes reported in the literature. The p.c.s. and p.q.s. values that are presented allow some significant insights to be made both in relation to the bonding interactions that all the various axial ligands, carbonyl ligands (both as mono and bis carbonyl complexes), other gaseous molecules, as well as the macrocyclic ligands themselves have on the low-spin iron(II) atom. In particular, the way CO binds to low-spin iron(II) porphyrins is shown to be a function of the nature, presence and stereochemistry of any nitrogenous base present as the second axial ligand. In the case when a CO is present as both the axial ligands new insights in the bonding are apparent that account for the positive sign in the electric field gradient for [Fe(pc)(CO)_2_], in which pc is phthalocyanine, whereas evidence is found from the *Δ*EQ for it to be negative in the [Fe(por)(CO)_2_] complexes.

Currently there is great interest in designing molecules for use in the fields of molecular electronics and molecular recognition. In both these areas it is important to understand how molecular architecture influences and controls bonding and, in this work, we have endeavoured to understand some of the electronic properties of low-spin octahedral [Fe(II)(PPIX)] complexes.

## Results and discussion

### Theory

Although the theory for deriving p.c.s. and p.q.s. values is well documented in the literature [73–75], it is worth giving brief details here, to illuminate the reader on some of the main points that support the approach. The Mössbauer chemical shift (often referred to as the isomer shift, *δ*) is a measure of the ‘s’ electron density at the nucleus. In iron(II) the nucleus is shielded by mainly ‘3d’ electrons, so the amount of ‘s’ electron density present is usually taken as a relative amount for a series of isostructural compounds (here relative would mean relative to the compound with the most ‘s’ electron density at the nucleus). Thus, for six-coordinate iron(II) in a macrocycle ligand it would be expected to be a comparable parameter where only the axial ligands vary. If the nature of the macrocycle is similar, it could also be used to compare the effect of changing the macrocycle.

If a nucleus has an angular momentum quantum number *I* > 1/2, then it has a non-spherical charge distribution. The consequence of this is a nuclear quadrupole moment. If the atom possessing this quadrupole moment is in the presence of an asymmetrical electric field (produced by an asymmetric electronic charge distribution or ligand arrangement) then this splits the nuclear energy levels. The resulting charge distribution is referred to as the Electric Field Gradient (EFG). For the case of an isotope with an *I* = 3/2 excited state, such as ^57^Fe, the excited state is split into two sub-states *m*_*I*_ =  ± 1/2 and *m*_*II*_ =  ± 3/2. This produces a Mössbauer spectrum that has two lines. The magnitude of the *ΔE*_*Q*_ is the distance between the two lines on the scale (given in mm s^−1^). The *ΔE*_*Q*_ thus refers to the charge distribution around the iron atom, (the electronic field gradient), this is due to the valence electrons and the distribution of the ligand charges and the lattice-charge effect. The later arises from other charged species in the crystal lattice or frozen solution. It should be obvious that if the *ΔE*_*Q*_ is close to or equal to zero for an ^57^Fe atom, then the electronic charge distribution around it is symmetrical or very nearly symmetrical. However, this does not mean that the atomic arrangement around the atom is symmetrical as the electronic charge distribution does not have to mirror the atomic positions. Crystal structural data shows the location of the atoms and their symmetry; it does not always tell (depending on how good the data is) us the total electronic bonding environment and the electron distribution in the bonds.

It has been demonstrated [73] that the total *δ* for a six-coordinate low-spin iron(II) complex is a simple sum of the p.c.s. values of the individual six ligating groups:

1 $$\text{c.s.}=\sum_{j=1}^{6}{ \left(\text{p.c.s.}\right)}_{j}.$$

The p.c.s. values can be found by dividing the*δ* values of appropriate octahedral complexes by six [73]. Such complexes if they have six identical ligands readily yield p.c.s. values for the ligand. These p.c.s. values thence obtained are characteristic for that ligand and can be used to derive p.c.s. values for other ligands in mixed ligand complexes.

The Mössbauer quadruple splitting (*ΔE*_*Q*_) values are useful in deriving the geometry of low-spin iron(II) complexes. For example, a 2:1 trans:cis *ΔE*_*Q*_ ratio has been found in both iron(II) and tin(II) compounds [73, 74, 83–86]. The ratio is predicted by the point charge model [72, 85, 86] a general ligand field model [87] and Bancroft et al. [73, 88] have discussed these and adopted the McClure molecular orbital approach [89].

For six-coordinate structures of tetradentate macrocycle ligands all complexes are trans, so geometry is not in doubt but p.q.s. values are still useful. They have been derived in full elsewhere [73] and so the reader is directed there. Only brief details will be summarised herein:- they showed [73] assuming true octahedral angles and an asymmetry parameter of zero for tetragonally distorted trans-FeA_4_B_2_ complexes that:

2 $$\Delta E_{Q} = \, + { 4}\left( {{\text{p.q.s.}}} \right)_{{\text{A}}} - { 4}\left( {{\text{p.q.s.}}} \right)_{{\text{B}}} .$$

As a tetragonal distortion will result from both long or short axial bonds then an interesting consequence can arise; that if the axial bonds are long the sign of the field gradient will be positive but as they get shorter and stronger the sign can switch to be negative. By choosing an appropriate reference value of − 0.30 mm s^−1^ for coordinated chloride and using the known signs of the *ΔE*_*Q*_ parameters for a range of complexes, Bancroft et al. [73] assigned p.q.s. values to a large number of ligands. Dabrowiak et al. [77] extended these by including imidazole and R_2_NH.

The *δ* and the *ΔE*_*Q*_ for the complexes reported in this work are presented in the tables along with those taken from the literature. The Mössbauer data for the [Fe(II)(Por)L_2_] and[Fe(II)(Por)LCO] complexes (or where Por is swapped for phthalocyanine macrocycle (Pc)) and those for important reference biological proteins [15–17, 21, 27, 28, 34, 37–40, 56, 66, 68, 80, 82, 89–118] appear in Tables 1, 2, 3, 4 and 5. The calculated values also appear in these tables. The derived p.c.s. and p.q.s. values appear in Tables 6 and 7. Also presented in Tables 3 and 5 are some of our recently published results on [Fe(II)(TPPS)L_2_ and [Fe(II)(TPPS)(CO)L] complexes where TPPS is meso-tetrakis(p-sulfonatophenyl)porphyrin [41]. We have previously shown from both visible spectra and frozen solution Mössbauer data at 78 K that [Fe(II)TPPS] and [Fe(II)(PPIX)] behave differently as a function of pH in aqueous solution clearly indicating that changes in the periphery of the porphyrin structure affects the chemistry at the Fe(II) centre [24, 29, 63]. We will refer to this later in this work where more evidence for the different behaviour of the two porphyrin rings is presented.

**Table 1:** ^57^Fe Mossbauer data for low-spin [Fe(TPP)L_2_] complexes

Compound	T °K	*δ*_obs_ mm s^−1^	*δ*_calc_ mm s^−1^	*ΔE*_Qobs_ mm s^−1^	*ΔE*_Qcalc_ mm s^−1^	Ref
[Fe(TPP)(py)_2_]	300	0.44	0.50^a^	1.22		[90]
	77	0.49		1.15	1.24	[90]
[Fe(TPP)(pip)_2_]	300	0.51	0.52^a^	1.52		[91]
	77	0.59		1.44	1.46^b^	[91]
[Fe(TPP)(Im)_2_]	77	0.54	0.59	1.06	1.00	[92]
[Fe(TPP)(1-VinylIm)_2_]	77	0.54	0.59	1.02	1.08	[93]
[Fe(TPP)(1-SiMe_3_Im)_2_]	77	0.55	0.59^c^	1.04	1.08	[93]
[Fe(TPP)(1-BzylIm)_2_]	77	0.54	0.59	1.02	1.08	[93]
[Fe(TPP)(1-AcIm)_2_]	77	0.54	0.59	0.97	0.96^d^	[93]
[Fe(TPP)(1-MeIm)_2_]	77	0.56	0.59	1.07	1.08^e^	[93]

TPPH_2_ = *α*, *β*, *γ*, *δ* – tetraphenylporphin; py = pyridine; pip = piperidine; Im = imidazole; 1-VinylIm = 1-vinylimidazole; 1-SiMe_3_Im = 1-(trimethylsilyl)imidazole; 1-BzylIm = 1-benzylimidazole; 1-AcIm = 1-acetylimidazole; and 1-MeIm = 1-methylimidazole.

^a^Used to calculate p.c.s. value for TPP ligand

^b^Used to calculate p.q.s. value for TPP ligand

^c^Used to calculate p.c.s. value for 1-RIm ligand

^d^Used to calculate p.q.s. value for 1-RIm ligand (R = electron donor)

^e^Used to calculate p.q.s. value for 1-R’Im ligand (R’ = electron acceptor)

**Table 2:** ^57^Fe Mossbauer data for low-spin [Fe(pc)L_2_] complexes

Compound	T °K	*δ*_obs_ mm s^−1^	*δ*_calc_ mm s^−1^	*ΔE*_Qobs_ mm s^−1^	*ΔE*_Qcalc_ mm s^−1^	Ref
[Fe(pc)(py)_2_]	295	0.34	0.34	2.02		[94]
	77	0.41		1.97	2.02	[94]
[Fe(pc)(but)_2_]	77	0.43	0.34^a^	1.94	1.90	[94]
[Fe(pc)(Im)_2_]	77	0.39	0.36^a^	1.75	1.78	[94]
[Fe(pc)(pip)_2_]	295	0.37	0.36	2.34	2.24	[82]
[Fe(pc)(but)_2_]	298	0.35	0.34	1.97	1.90	[80]
[Fe(pc)(^s^but)_2_]	298	0.35	-	2.05	1.85	[80]
[Fe(pc)(NH_3_)_2_]	298	0.35	0.35^c^	1.79	1.78^d^	[80]
[Fe(pc)(Morph)_2_]	295	0.35	0.34^e^	2.31	2.38	[80]
[Fe(pc)(prNH_2_)_2_]	295	0.34	0.34^e^	1.97	1.90^d^	[94]
[Fe(pc)(OHCH_2_CH_2_NH_2_)_2_]	295	0.36	0.36	2.01	2.05	[94]

Pc = phthalocyanine; but = n-butylamine; ^s^but = secondary butylamine; Morph = morpholine; Et_2_NH = diethylamine; PrNH_2_ = propylamine; OHCH_2_CH_2_NH_2_ = ethanolamine

^a^Used to calculate p.c.s. value for pc macrocycle

^b^Used to calculate p.q.s. value for pc macrocycle

^c^Used to calculate p.c.s. value for Et_2_NH ligand

^d^Used to calculate p.q.s. value for R_2_NH ligand

^e^Used to calculate p.c.s. value for RNH_2_ ligand

* Many other six-coordinate Fe(pc)L_2_complexes are listed in Refs. [79–81]. The p.c.s. and p.q.s. values quoted in these references agree with those derived herein

**Table 3:** ^57^Fe Mossbauer data for low-spin [Fe(Por)L_2_] complexes

Compound	T °K	*δ*_obs_ mm s^−1^	*δ*_calc_ mm s^−1^	*Δ*E_Qobs_ mm s^−1^	*Δ*E_Qcalc_ mm s^−1^	Ref
[Fe(PMXPP)(py)_2_]	298	0.46	0.47^a^	1.27	1.28^b^	[95, 96]
[Fe(PMXPP)(pip)_2_]	298	0.51	0.49^a^	1.49	1.50	[95]
[Fe(PMXPP)(Enim)_2_]	298	0.46	0.47^c^	1.05	1.06^d^	[95]
[Fe(PMXPP)(Pydn)_2_]	298	0.51	0.49^c^	1.33	1.34^d^	[95]
[Fe(PMXPP)(DMM)_2_]	298	0.52	0.50^c^	1.52	1.52^d^	[95]
[Fe(PMXPP)(Morph)_2_]	298	0.51	0.50^c^	1.54	1.54^d^	[95]
[Fe(PMXPP)(THpy)_2_]	298	0.50	0.49^c^	1.40	1.40^d^	[95]
[Fe(PMXPP)(3-Mepip)_2_]	298	0.49	0.48^c^	1.45	1.44^d^	[95]
[Fe(PMXPP)(4-NMe_2_py)_2_]	298	0.47	0.49	1.20	1.16^d^	[96]
[Fe(PMXPP)(4-Acpy)_2_]	298	0.47	0.48	1.22	1.24^d^	[96]
[Fe(PMXPP)(3-Acpy)_2_]	298	0.49	0.48	1.26	1.28^d^	[96]
[Fe(PMXPP)(4-CNpy)_2_]	298	0.48	0.48	1.27	1.24	[96]
[Fe(PMXPP)(4-Mepy)_2_]	298	0.49	0.48	1.29	1.24	[96]
[Fe(PMXPP)(3-CNpy)_2_]	298	0.49	0.48	1.29	1.28	[96]
[Fe(PMXPP)(3,5-Me_2_py)_2_]	298	0.50	0.50^c^	1.31	1.28	[96]
[Fe(TMXPP)(py)_2_]	78	0.51		1.21		[68]
[Fe(TMXPP)(pip)_2_]	78	0.56		1.48		[68]
[Fe(PMEPP)(pip)_2_]	298	0.51	0.51^e^	1.53	1.54^f^	[95]
[Fe(PMEPP)(Enim)_2_]	298	0.49	0.49	1.10	1.10	[95]
[Fe(PMEPP)(Pydn)_2_]	298	0.51	0.51	1.36	1.38	[95]
[Fe(PMEPP)(DMM)_2_]	298	0.48	0.52	1.39	1.56	[95]
[Fe(PClPP)(pip)_2_]	298	0.49	0.49^e^	1.47	1.47^f^	[95]
[Fe(PClPP)(Enim)_2_]	298	0.46	0.47^e^	1.03	1.03	[95]
[Fe(PClPP)(Pydm)_2_]	298	0.49	0.49	1,27	1.31	[95]
[Fe(PClPP)(DMM)_2_]	298	0.49	0.50	1.53	1.49	[95]
[Fe(PClPP)(Morph)_2_]	298	0.50	0.50	1.52	1.51	[95]
[Fe(PClPP)(THpy)_2_]	298	0.49	0.49	1.37	1.37	[95]
[Fe(PClPP)(3-Mepip)_2_]	298	0.49	0.48	1.42	1.41	[95]
[Fe(PClPP)(4-Mepip)_2_]	298	0.50	0.50^c^	1.46	1.45^d^	[95]
[Fe(PClPP)(py)_2_]	298	0.45	0.47	1.27	1.25	[95]
[Fe(OEP)(py)_2_]	295	0.47	0.45^e^	1.21	1.22^f^	[97]
[Fe(OEP)(3-Mepy)_2_]	295	0.49	0.46^c^	1.24	1.18	[97]
[Fe(OEP)(4-Mepy)_2_]	295	0.47	0.46^c^	1.18	1.18	[97]
[Fe(OEP)(NH_3_)_2_]	295	0.50	0.45^c^	1.18	0.98	[97]
[Fe(OEP)(2-MeHIm)_2_]	77	0.43	0.45	1.67	1.67	[98]
[Fe(OEP)(4-NMe_2_py)_2_]	77	0.54	0.54	1.02	1.10	[98]
[Fe(OEP)(4-CNpy)_2_]	77	0.41	0.53	1.10	1.18	[98]
[Fe(OEP)(1-MeIm)_2_]	77	0.55	0.54	0.96	1.06	[98]
[Fe(OMTBP)(py)_2_]	295	0.51	0.48^e^	0.73	0.75^f^	[99]
[Fe(OMTBP)(3-Mepy)_2_]	295	0.51	0.50	0.81	0.71	[99]
[Fe(OMTBP)(pip)_2_]	115	0.53	0.57^e^	0.89	0.97	[82]
[Fe(TMP)(1-MeIm)_2_]	120	0.52	0.57	1.11	1.09	[100]
[Fe(TMP)(4-CNpy)_2_]	120	0.50	0.56	1.13	1.21	[100]
[Fe(TMP)(3-Clpy)_2_]	120	0.52	0.56	1.23	1.21	[100]
[Fe(TMP)(4-Mepy)_2_]	120	0.51	0.57	1.12	1.21	[100]
[Fe(TMP)(4-NMe_2_py)_2_]	120	0.45	0.57	1.27	1.13	[100]
[Fe(TMP)(2-MeHIm)_2_]	77	0.48	0.49	1.64	1.70	[100]
[Fe(TMP)(2-MeHIm)_2_]	298	0.45	0.42	1.78	1.70	[20]
[Fe(TMP)(1,2-Me_2_Im)_2_]	77	0.48	0.49	1.73	1.73	[100]
[Fe(TPPS)(Im)_2_]	78	0.54	0.55	0.97	0.99	TW
[Fe(TPPS)(py)_2_]	78	0.53	0.53	1.13	1.23	[41]
[Fe(TPPS)(2-Mepy)_2_]	78	0.53	0.49	1.11	1.15	TW
[Fe(TPPS)(4-Mepy)_2_]	78	0.53	0.54	1.15	1.19	[41]
[Fe(TPPS)(3,4-Me_2_py)_2_]	78	0.54	0.53	1.18	1.19	[41]
[Fe(TPPS)(3-MeNHpy)_2_]	78	0.52	0.54	1.15	1.19	[41]
[Fe(TPPS)(pip)_2_]	78	0.55	0.55	1.38	1.45	[41]
[Fe(TPPS)(3-Mepip)_2_]	78	0.54	0.54	1.42	1.39	TW
[Fe(TPPS)(but)_2_]	78	0.53	0.53	1.10	1.11	TW
[Fe(TPPS)(Pydn)_2_]	78	0.56	0.55	1.22	1.29	TW
[Fe(TPPS)(4-NHTRIZ)_2_]	78	0.52	0.51	1.05	1.04	[41]
[Fe(TPPS)(GEE)_2_]	78	0.52	0.55	1.13	1.18	[66]
[Fe(TNPS)(Im)_2_]	78	0.52	0.53	1.05	0.98	TW
[Fe(TNPS)(py)_2_]	78	0.50	0.51	1.23	1.22	TW
[Fe(TNPS)(Pydn)_2_]	78	0.55	0.53	1.24	1.28	TW
[Fe(TNPS)(pip)_2_]	78	0.54	0.53	1.41	1.44	TW
[Fe((SP)_2_(SMeP)_2_P)(Im)_2_]	78	0.54	0.54	1.00	1.00	TW
[Fe((SP)_2_(SMeP)_2_P)(py)_2_]	78	0.53	0.52	1.15	1.22	TW
[Fe((SP)_2_(SMeP)_2_P)(4-Mepy)_2_]	78	0.50	0.53	1.15	1.18	TW
[Fe((SP)_2_(SMeP)_2_P)(3,4-Me_2_py)_2_]	78	0.53	0.52	1.12	1.18	TW
[Fe((SP)_2_(SMeP)_2_P)(Pydn)_2_]	78	0.56	0.54	1.21	1.28	TW
[Fe((SP)_2_(SMeP)_2_P)(pip)_2_]	78	0.55	0.54	1.44	1.44	TW
[Fe((SP)_2_(SMeP)_2_P)(3-Mepip)_2_]	78	0.54	0.54	1.43	1.38	TW
[Fe(TNP)(Im)_2_]	78	0.55	0.53	0.98	0.98	TW
[Fe(TNP)(py)_2_]	78	0.49	0.51	1.16	1.22	TW
[Fe(TNP)(4-Mepy)_2_]	78	0.52	0.52	1.15	1.18	TW
[Fe(TNP)(pip)_2_]	78	0.52	0.53	1.43	1.44	TW
[Fe(TpivPP)(1-MeIm)_2_]	298	0.44	0.45	1.05	1.07	[101]
[Fe(TpivPP)(1-EtIm)_2_]	298	0.46	0.45	1.07	1.07	[101]
[Fe(TpivPP)(1-VinylIm)_2_]	298	0.44	0.45	1.07	1.07	[101]
[Fe(MbenTpivPP)(1-MeIm)_2_]	298	0.43		0.98		[104]
[Fe(TImPP)(1-MeIm)_2_]	295	0.43		1.03		[105]
[Fe(TFPPBr_8_)(1-MeIm)_2_]	295	0.41		1.11		[106]
[Fe(PMXPP)(amp)_2_]	25	0.55*	0.55	1.04	1.04	[107]
[Fe(TFPP)(Fe(C_5_H_5_)(C_4_H_4_N))_2_]	77	0.53		1.25		[70]

PMXPPH_2_ = *α*, *β*, *γ*, *δ* – tetra(p-methoxyphenyl)porphin; TMXPPH_2_ = *α*, *β*, *γ*, *δ* – tetra(2,4,6-methoxy-phenyl)porphin; PMEPPH_2_ = *α*, *β*, *γ*, *δ* – tetra(p-tolyl)porphin; 102H_2_ = *α*, *β*, *γ*, *δ* – tetra(p-chlorophenyl)porphin; OEP = octaethylporphyrin; OMTBPH_2_ = octamethyltetrabenzoporphyrin; TMPH_2_ = tetramesitylporphin; TPPSH_2_ = *α*, *β*, *γ*, *δ* – tetra(p-sulfophenyl)porphin; TNPSH_2_ = *α*, *β*, *γ*, *δ* – tetra(p-sulfo 1-napthyl)porphin; (SP)_2_(SMeP)_2_PH_2_ = *α*, *γ* bis(p-sulfophenyl) – *β*, *δ* bis(m-sulfo-p-tolyl)porphin; TNPH_2_ = *α*, *β*, *γ*, *δ* – tetra 1-napthylporphin; TpivPPH_2_ = “Picket Fence” porphyrin, meso-tetrakis(*α*,*α*,*α*,*α*-o-pivalamidophenyl)porphyrin (TpivPP); MbenTpivPPH_2_ = benzoyl modified “Picket Fence” porphyrin TImPPH_2_ = *α*,*α*,*α*,*α*-o-(1-methylimidazole-5-carboxylaminophenyl)porphin; TFPPBr_8_H_2_ = fully halogenated(on the vinyl rings *α*, *β*, *γ*, *δ* – tetra(pentafluorophenl)porphin; TFPPH_2_ = *α*, *β*, *γ*, *δ* – tetra(pentafluorophenyl)porphin; Enim = ethylenimine; Pydn = pyrrolidine; DMM = 2,6- dimethylmorpholine; Morph = THpy = tetrahydropyridine; 3-Mepip = 3-methylpiperidine; 4-NMe_2_py = 4-dimethylaminopyridine; 4-Acpy = 4-acetylpyridine; 3-Acpy = 3-acetylpyridine; 4-CNpy = 4-cyanopyridine; 4-Mepy = 4-methylpyridine; 3-CNpy = 3- cyanopyridine; 3,5-Me_2_py = 3,5-dimethylpyridine; 4-Mepip = 4-methylpiperidine; 3-Mepy = 3-methylpyridine; 2-MeHIm = 2-methylimidazole; 3-Clpy = 3-chloropyridine; 1,2-Me_2_Im = 1,2-dimethylimidazole; 2-Mepy = 2-methylpyridine; 3.4-Me_2_py = 3,4-dimethylpyridine; 3-MeNHpy = 3-methylaminopyridine; 4-NHTRIZ = 4-amino 1,2,4 triazole; GEE = glycine ethylester; 1-EtIm = 1- ethylimidazole; amp = 4-(2aminoethyl)morpholine; Fe(C_5_H_5_)(C_4_H_4_N) = azaferrocene;

^a^Used to calculate p.c.s. value for Por = PMXPP

^b^Used to calculate p.q.s. value for Por = PMXPP

^c^Used to calculate p.c.s. value for axial ligands

^d^Used to calculate p.q.s. value for axial ligands

^e^Used to calculate p.c.s. value for (Por = PMEPP, PClPP, OEP, or OMTBP)

^f^Used to calculate p.q.s. value for (Por = PMEPP, PClPP, OEP, or OMTBP). TW = This work

*We have changed this data to be relative to stainless steel, though the authors do not say if it is relative to natural Fe foil. We note that the spectral data are not presented with errors and the fit looks a little rough due to poor data. We calculated the p.c.s. and p.q.s. values for amp using the derived values for PMXPP given in Table 6 below. We chose not to include this data in Fig. 7 as the *ΔE*_Q_ value is possibly in error.

**Table 4:** ^57^Fe Mossbauer data for low-spin [Fe(PPIX)L_2_] complexes

Compound	T °K	*δ*_obs_ mm s^−1^	*δ*_calc_ mm s^−1^	*ΔE*_Qobs_ mm s^−1^	*ΔE*_Qcalc_ mm s^−1^	Ref
[Fe(PPIX)(py)_2_]	77	0.54	0.56^a^	1.21	1.20^b^	[92]
[Fe(PPIX)(pip)_2_]	295	0.51	0.52a	1.43	–	[92]
	77	0.58	–	1.42	1.42	[92]
[Fe(PPIX)(Im)_2_]	77	0.51	0.59	0.95	0.96	[92]
[Fe(PPIX)(MeNH_2_)_2_]^c^	78	0.56	0.57	1.08	1.08	[39]
[Fe(PPIX)(EtNH_2_)_2_]^c^	78	0.56	0.57	1.08	1.08	[39]
[Fe(PPIX)(Et_2_NH)_2_]^c^	78	0.54	0.58	1.07	1.16	[39]
[Fe(PPIX)(OHCH_2_CH_2_NH_2_)_2_]^c^	78	0.56	0.59	1.09	1.08	[39]
[Fe(PPIX)(NH_2_CH_2_)_2_]^c^	78	0.61	0.60	1.15	1.08	[39]
[Fe(PPIX)(prNH_2_)_2_]^c^	78	0.58	0.57	1.09	1.08	[39]
[Fe(PPIX)(n-but)_2_]^c^	78	0.57	0.57	1.03	1.08	[39]
[Fe(PPIX)(s-but)_2_]^c^	78	0.57	0.57	1.09	1.03	[39]
[Fe(PPIX)(oct)_2_]^c^	78	0.57	0.57	1.09	1.08	[39]
[Fe(PPIX)(5-Cl,1-MeIm)_2_]^c^	78	0.52	0.54^b^	0.97	1.00	[38]
[Fe(PPIX)(4-Mepy)_2_]^c^	78	0.57	0.58	1.17	1.16	[38]
[Fe(PPIX)(3,4-Mepy)_2_]^c^	78	0.55	0.57^d^	1.15	1.16^e^	[38]
[Fe(PPIX)(4-Clpy)_2_]^c^	78	0.55	0.58	1.23	1.16	[38]
[Fe(PPIX)(3-NHMepy)_2_]^c^	78	0.54	0.58	1.14	1.20	[38]
[Fe(PPIX)(isoquin)_2_]^c^	78	0.53	0.56^d^	1.11	1.12^e^	[38]
[Fe(PPIX)(CME)_2_]^c^	80	0.60	0.58	1.22	1.18	[28]
[Fe(PPIX)(GEE)_2_]^c^	80	0.58	0.59	1.15	1.15	[28]
[Fe(PPIX)(His)_2_]^c^	80	0.51	0.53	0.88	0.88^e^	[37]
[Fe(PPIX)(NAHis)_2_]^c^	80	0.53	0.55^d^	1.04	1.04^e^	[37]
[Fe(PPIX)(pilocarpate)_2_]^c^	80	0.55	0.56	1.04	1.04^e^	[37]
[Fe(PPIX)(histamine)_2_]^c^	80	0.57	0.59	1.04	1.04^e^	[37]
[Fe(PPIX)(2-Mepip)_2_]^c^	80	0.52	0.52	1.17	1.17	[28]
[Fe(PPIX)(1-MeIm)_2_]^c^	78	0.56	0.57	1.03	1.04	[34]
[Fe(PPIX)(2-MeIm)_2_]^c, f^	78	0.60	0.60	1.26	1.26	[34]
[Fe(PPIX)(4(3H|)|pyr)_2_]^c^	78	0.51		1.20		[40]
[Fe(PPIX)(5-Mepyr)_2_]^c^	78	0.51		0.98		[40]
[Fe(PPIX)(2-Mepyra)_2_]^c^	78	0.50		1.07		[40]
[Fe(PPIX)(2-Meopyra)_2_]^c^	78	0.54		1.14		[40]
[Fe(PPIX)(3-Mepyrida)_2_]^c^	78	0.56		1.09		[40]
[Fe(PPIX)(THZ)_2_]^c^	78	0.51		1.08		[40]
[Fe(PPIX)(OXZ)_2_]^c^	78	0.55		0.94		[40]
[Fe(PPIX)(4-n-butTRIZ)_2_]^c^	78	0.54	0.54	1.00	1.01	[40]
[Fe(PPIX)(4-NHTRIZ)_2_]^c^	78	0.55	0.55	1.01	1.01	[40]
[Fe(PPIX)(1-Cl_2_phTRIZ)_2_]^c^	78	0.54		0.91		[40]
[Fe(PPIX)(3-N(TTZMeac)_2_]^c^	78	0.55	0.55	1.07	1.07	[40]
[Fe(PPIX)(2-MeIm)_2_]^c^	78	0.60	0.60	0.96	0.96	[40]
[Fe(PPIX)(2-Mepy)_2_]^c^	78	0.53	0.53	1.12	1.12	[40]
[Fe(PPIX)(^t^but)_2_]^c^	78	0.63	0.63	1.09	1.09	[40]

PPIX = protoporphyrin IX; MeNH_2_ = methylamine; EtNH_2_ = ethylamine; Et_2_NH = di-ehylamine; (NH_2_CH_2_) = 1,2-diamino-ethane; oct = octylamine; 5-Cl,1-MeIm = 5-Chloro,1-methylimidazole; 4-ClPy = 4-chloropyridine; 3-NHMepy = 3-aminomethylpyridine; isoquin = isoquinoline; CME = cysteine methylester; His = histidine; NAHis = N-α-acetyl histidine; 2-Mepip = 2-methylpiperidine; 2-MeIm = 2-methylimidazole. 4(3H)pyr = 4-hydroxypyrimidine; 5-Mepyr = 5-methylpyrimidine; 2-Mepyra = 2-methylpyrazine; 2-Meopyra = 2-methoxypyrazine; 3-Mepyrida = 3-Methylpyridazine; THZ = thiazole; OXZ = oxazole; 4-n-butTRIZ = 4-n-butyl 1,2,4 triazole; 1-Cl_2_phTRIZ = 1-(2,4-dichlorophenyl)-2-(1-N-1,2,4-triazole)-3-(hydroxy)-4-(4dimethyl)pentane; 3.N(TTZMeac) = 3-N(1,2,3,4 tetrazole)-1-methylacetate; 2-Mepy = 2-methylpyridine; ^t^but = tertiary butylamine

^a^Used to calculate p.c.s. value for PPIX

^b^Used to calculate p.q.s. value for PPIX

^c^Complex studied in frozen solution, Each solution had a pH of 12 at room temperature prior to freezing

^d^Used to calculate p.c.s. value for axial ligands

^e^Used to calculate p.q.s. value for axial ligands

^f^This was from frozen solution (pH = 10) of detergent 5% ethyltrimethyl-ammonium bromide, the five-coordinated high-spin complex was also present at 33% of the total present. In the absence of the detergent in aqueous solution at pH = 10 [Fe(PPIX)(2-MeIm)_2_] does not form

^g^This frozen solution contained both [Fe(PPIX)(2-MeIm)_2_] and five-coordinate high-spin [Fe(PPIX)(2-MeIm)]. The latter was much more abundant from an inspection of the spectrum. The parameters of both these species were different to those reported in Ref. 34b where the frozen solution was a detergent at pH = 10

**Table 5:** ^57^Fe Mossbauer data for low-spin [Fe(Por)L(CO)] complexes and [Fe(Por)(CO)_2_] complexes

Compound	T °K	*δ*_obs_ mm s^−1^	*δ*_calc_ mm s^−1^	*ΔE*_Qobs_ mm s^−1^	*ΔE*_Qcalc_ mm s^−1^	^υ^CO cm^−1^	Ref
[Fe(TPP)(1-MeIm)(CO)]	77	0.29^a^	0.36	0.35	0.42	1968	[108, 109]
[Fe(TPP)(py)(CO)]	77	0.37^a^	0.35	0.57	0.50	1980	[108, 109]
[Fe(TPP)(pip)(CO)]	295	0.27	0.29	0.53	0.61		[82]
[Fe(TPP)(1-MeIm)(CO)]^b^	293	0.25	0.28	0.35	0.42	1968	[109]
[Fe(TPP)(1,2-MeIm)(CO)]^b^	293	0.26	0.245	0.71	0.74	1953	[109]
[Fe(PMXPP)(Morph)(CO)]	298	0.32	0.295	0.55	0.57	1996	[110]
[Fe(PMXPP)(pip)(CO)]	298	0.30	0.29	0.49	0.55	1981	[110]
[Fe(PMXPP)(py)(CO)]	298	0.29	0.28	0.49	0.44	1978	[110]
[Fe(PMXPP)(Pydn)(CO)]	298	0.28	0.29	0.45	0.47	1982	[110]
[Fe(PMXPP)(Im)(CO)]	298	0.28	0.29	0.36	0.36	1966	[110]
[Fe(TpivPP)(1-MeIm)(CO)]	4.2	0.29(to 298)		0.27	0.36	1964	[111, 112]
[Fe(TPPS)(GEE)(CO)]	78	0.30(to 298)	0.29	0.37	0.48		[66]
[Fe(TPPS)(py)(CO)]	78	0.37	0.36	0.56	0.51		[41]
[Fe(TPPS)(4-Mepy)(CO)]	78	0.36	0.37	0.49	0.49		[41]
[Fe(TPPS)(3,4-Me_2_py)(CO)]	78	0.37		0.57	0.49		[41]
[Fe(TPPS)(3-MeNHpy)(CO)]	78	0.39		0.43	0.49		[41]
[Fe(TPPS)(Pydn)(CO)]	78	0.40		0.42	0.54		[41]
[Fe(TPPS)(pip)(CO)]	78	0.41	0.37	0.57	0.62		[41]
[Fe(TNPS)(Im)(CO)]	78	0.32		0.40			TW
[Fe((SP)_2_(SMeP)_2_P)(Im)(CO)]	78	0.32		0.25			TW
[Fe((SP)_2_(SMeP)_2_P)(py)(CO)]	78	0.37		0.44			TW
[Fe((SP)_2_(SMeP)_2_P)(Pydn)(CO)]	78	0.36		0.46			TW
[Fe(OMTBP)(pip)(CO)]	295	0.32	0.31	0.20	0.19		[82]
[Fe(OMTBP)(1-MeIm)(CO)]	295	0.29	0.30	0.0	0.0		[82, 102]
[Fe(OEP)(1-MeIm)(CO)]^b^	293	0.27	0.27	0.40	0.40	1965	[109]
[Fe(OEP)(CO)_2_]	298	0.27	(0.27)	0.18	-0.26	2021	[113]
[Fe(PPIX)(GEE)(CO)]	80	0.29(to 298)	0.29	0.42	0.48		[27]
[Fe(PPIX)(CME)(CO)]	80	0.28(to 298	0.285	0.49	0.49		[27]
[Fe(PPIX)(pip)(CO)]	295	0.27	0.29	0.62	0.61		[82]
[Fe(PPIX)(Im)(CO)]^c^	78	0.35	0.36	0.27	0.36		[40]
[Fe(PPIX)(EtNH_2_)(CO)]^c^	78	0.35	0.35	0.33	0.42		[40]
[Fe(PPIX)(NH_2_CH_2_)(CO)]^c^	78	0.42	0.365	0.41	0.45		[40]
[Fe(PPIX)(but)(CO)]^c^	78	0.35	0.35	0.38	0.42		[40]
[Fe(PPIX)(s-but)(CO)]^c^	78	0.32	0.35	0.39	0.40		[40]
[Fe(PPIX)(4-Mepy)(CO)]^c^	78	0.42	0.355	0.58	0.44		[40]
[Fe(PPIX)(4-n-butTRIZ)(CO)]^c^	78	0.38	0.355	0.43	0.39		[40]
[Fe(PPIX)(4-NHTRIZ)(CO)]^c^	78	0.37	0.34	0.43	0.42		[40]
[Fe(PPIX)(3.N(TTZMeac))(CO)]^c^	78	0.37	0.33	0.61	0.42		[40]
[Fe(PPIX)(pip)(CO)]^c^	78	0.37	0.36	0.57	0.61		[40]
[Fe(PPIX)(2-MeIm)(CO)]^c^	78	0.31	0.365	0.36	0.36		[40]
[Fe(PPIX)(2-Mepy)(CO)]^c^	78	0.35	0.33	0.59	0.44		[40]
[Fe(PPIX)(t-but)(CO)]^c^	78	0.45	0.38	0.57	0.43		[40]
MbCO	4.2	0.29		0.35	0.36	1933, 1945	[15, 16, 114]
HbCO	4.2	0.28		0.36	0.36	1951	[17, 115]
CytochromeP450_cam_CO	200	0.25	0.34	0.34	0.36		[116]
	4.2	0.29	0.32				
[Fe(TPP)(CO)_2_]	295	0.28	(0.28)	0.27	− 0.24	2030	[117]
[Fe(OMTBP)(CO)_2_]	295	0.39	(0.39)	− 0.49	− 0.56	2025	[117]
[Fe(PPIX)(CO)_2_]^c^	78	0.33	(0.28)	0.53	− 0.20		TW
[Fe(pc)(py)(CO)]	295	0.20	0.20	1.19	1.14	2000	[118]
[Fe(pc)(pip)(CO)]	295	0.20	0.21	1.27	1.25	1992	[82, 118]
[Fe(pc)(NH_3_)(CO)]	295	0.21	0.20	1.02	1.02	2006	[118]
[Fe(pc)(n-prNH_2_)(CO)]	295	0.19	0.20	1.11	108	1992	[118]
[Fe(pc)(CO)_2_]	295	0.18	(0.18)	0.82	0.26	2057	[118]

^υ^CO = infrared CO-stretching frequencies

^a^*δ* value not given in ref 108 but estimated from spectra accuracy not high

^b^Crystal structure contains a solvent molecule

^c^*δ*_obs_ value calculated to be relative to stainless steel by adding 0.9 mm s^−1^

**Table 6:** Partial centre shifts and partial quadrupole splittings for ligands bound to octahedral low-spin iron(II)^a^ in [Fe(Por)L_2_] complexes

Monodentate N ligands, CN or CS ligands	p.c.s (mm s^−1^)	p.q.s (mm s^−1^)	Macrocyclic ligand	p.c.s (mm s^−1^)	p.q.s (mm s^−1^
Im	0.08^b^ (0.10)^c^	(− 0.46)^c^ − 0.52^d^	0.25 Pc	0.05^d^	− 0.965^d^
Pip	0.08^b^	− 0.405^d^	0.25PMEPP	0.0875^d^	− 0.79^d,e^
Py	0.07^d^ (0.05)^b^	− 0.46^d^	0.25PMXPP	0.0825^d^	− 0.78^d,e^
NH_3_	0.07^d^	− 0.52^d^	0.25PClPP	0.0825^d^	− 0.775^d,e^
n-but	0.07^d^	− 0.49^d^	0.25TMP	0.0875^d^	− 0.7725^d,e^
Et_2_NH	0.075^d^	− 0.492^d^	0.25TPP	0.09^d^	− 0.77^d^
PrNH_2_	0.07^d^	− 0.49^d^	0.25TpivPP	0.0725^d^	− 0.7675^d^
			0.25TPPS	0.08^d^	− 0.7675^d^
RNH_2_^f^	0.07^d^	− 0.49^d^	0.25TNPS	0.075^d^	− 0.765^d,e^
(CH_2_NH_2_)_2_	0.085^d^	− 0.472^d^	0.25TNP	0.075^d^	− 0.765^d^
OHCH_2_CH_2_NH_2_	0.08^d^	− 0.4525^d^	0.25(SP)_2_(SMeP)_2_P	0.0775^d^	− 0.765^d,e^
			0.25OEP	0.0775^d^	− 0.765^d^
s-but	0.07^d^	− 0.5025^d^	0.25PPIX	0.09^d^	− 0.76
t-but	0.10^d^	− 0.4875^d^	0.25OMTBP	0.0825	− 0.64
CME	0.075^d^	− 0.465^d^			
GEE	0.08^d^	− 0.472^d^			
THpy	0.08^d^	− 0.433^d,e^	Pc	0.20^d^	− 3.86^d^
4-R^*^py^g^	0.075	− 0.47^d,e^			
3-R^**^py^h^	0.075^d^	− 0.47^d,e^	PMEPP	0.35^d^	− 3.16^d,e^
2-Mepy	0.05^d^	− 0.48^d^	PMXPP	0.33^d^	− 3.12^d,e^
3,5-Me_2_py	0.085^d^	− 0.465^d^	PClPP	0.33^d^	− 3.10^d,e^
3,4-Me_2_py	0.07^d^	− 0.47^d^	TMP	0.35^d^	− 3.09^d,e^
4-NMe_2_py	0.08	− 0.49	TPP	0.36^d^	− 3.08
Isoquin	0.065^d^	− 0.49^d^	TpivPP	0.29^d^	− 3.07^d^
			TPPS	0.32^d^	− 3.07^d^
			TNPS	0.30^d^	− 3.06^d^
1-R’Im^i^	0.08^d^	− 0.50^d^	TNP	0.30^d^	− 3.06^d^
1R”Im^j^	0.08^d^	− 0.53^d^	(SP)_2_(SMeP)_2_P	0.31^d^	− 3.06^d,^
5-Cl,1-MeIm	0.055^d^	− 0.51^d^	OEP	0.31^d^	− 3.06^d,e^
2-MeIm	0.085^d^	− 0.52^d^	PPIX	0.36^d^	− 3.04^d^
2-MeHIm	0.035^d^	− 0.3475^d^	OMTBP	0.33^d^	− 2.59^d,e^
1,2-Me_2_Im	0.035^d^	− 0.34^d^			
His	0.05^d^	− 0.50^d^			
Histamine	0.08^d^	− 0.50^d^			
Philocarpate	0.065^d^	− 0.50^d^			
4-n-butTRIZ	0.055^d^	− 0.51^d^			
4-NHTRIZ	0.06^d^	− 0.5075^d^			
3N(TTZMeac)	0.06^d^	− 0.4925^d^			
4-Mepip	0.085^d^	− 0.41^d,e^			
3-Mepip	0.075^d^	− 0.42^d,e^			
2-Mepip	0.045^d^	− 0.4675^d^			
Enim	0.07^d^	− 0.515^d,e^			
Pydn	0.08^d^	− 0.445^d,e^			
DMM	0.085^d^	− 0.40^d,e^			
Morph	0.085^d^	− 0.395^d,e^			
NO_2_	0.05	− 0.57			
NO	− 0.1	− 0.665			
CN(TPP)	0.02	− 0.73			
CN(PPIX)	0.02	− 0.76			
CN(TPPS)	0.02	− 0.77			
CS	− 0.12	− 0.80			

^a^All values calculated from *δ* relative to stainless steel at 298°K

^b^Value according to Bancroft et al., (Ref. [73])

^c^Value according to Dabrowiak et al., (Ref. [77]). Value in parenthesis not used in this work

^d^Value according to this work

^e^Calculated from room temperature data

^f^Value for RNH_2_ ligands where R is an aliphatic substituent

^g^R* = any substituent on the 4 position of py

^h^ R** = any substituent on the 3 position of py

^i^R’ = electron donor

^j^R” = electron acceptor

**Table 7:** Partial centre shifts and partial quadrupole splittings for CO and macrocyclic ligands in octahedral low-spin iron(II)^a^ in [Fe(Por)(CO)L] complexes

Monodentate CO Ligand for listed macrocycle	p.c.s (mm s^−1^)	p.q.s (mm s^−1^)	Macrocycle	p.c.s (mm s^−1^)	p.q.s (mm s^−1^)
CO(Pc)	0.0	− 0.90	0.25 Pc	0.0333	− 0.965
CO(PMXPP)	0.0	− 0.88	0.25PMXPP	0.0525	− 0.78
CO(TPP)	0.0	− 0.83	0.25TPP	0.0525	− 0.77
CO(TPPS)	0.0	− 0.83	0.25TPPS	0.055	− 0.7675
CO(OEP)	0.0	− 0.83	0.25OEP	0.05	− 0.765
CO(PPIX	0.0	− 0.81	0.25PPIX	0.0525	− 0.76
CO(OMTBP)	0.0	− 0.78	0.25OMTBP	0.0575	− 0.6475
			Pc	0.13	− 3.86
			PMXPP	0.21	− 3.12
			TPP	0.21	− 3.08
			TPPS	0.22	− 3.07
			OEP	0.20	− 3.06
			PPIX	0.21	− 3.04
			OMTBP	0.23	− 0.259

### p.c.s. data

To calculate the p.c.s. values, starting values were taken from the work of Bancroft et al. [73], where appropriate and modified where necessary to best fit the data used in this work (Table 6). All *δ* data were corrected relative to stainless steel at 293 °K where possible or 77 °K so that the derived p.c.s. data can be readily compared to previously derived values.

To discuss the variation in bonding properties of the various ligands it is necessary to assume differences in*δ* are due solely to differences in *δ* as did Bancroft et al. [73]

3 $${\text{As }}\delta \, = \, \delta . \, + {\text{ s.o.d.}},$$

where s.o.d. is the second-order Doppler shift and includes the zero-point motion contribution. The i.s. is dependent on the s-electron density at the nucleus and is thus sensitive to the strength of the chemical bonds formed by the Mössbauer atom. The s.o.d. is dependent on the structure of the solid and will vary from one solid to another [73]. It has been pointed out that the temperature dependence of the *δ* of octahedral low-spin iron(II) complexes are all similar [73] (0.07 ± 0.02 mm s^−1^) suggesting that the temperature dependent part of the s.o.d. shift and any possible temperature dependence of the i.s. are nearly constant.^73^ Thus when only 77 °K *δ* data are available, they are used in the present calculations by subtracting 0.07 mm s^−1^ from the values. When the calculated value of i.s. is then shown for the compound (for comparison to 77 °K data), 0.07 mm s^−1^ is added to it. Many of the data are room temperature and thus the latter addition is not necessary. (It should be noted that all the *δ* values given in Tables 1, 2, 3, 4 and 5 are corrected to stainless steel as a reference. This keeps them in agreement with previous work).

Several p.c.s. values were taken from refs 73 and 77 (see Table 6) as a starting set and all other p.c.s. values were found by using these with the series of compounds in Tables 1, 2, 3, 4 and 5. We estimate that the errors on the p.c.s. values are less than ± 0.01 mm s^−1^ as most of the complexes only have an i.s. error value of ± 0.01 mm s^−1^.

It is assumed from inspection of the data in Table 3 that the 1-RIm ligands (where R = Vin, SiMe_3_, Bzl, Ac or Me) all have the same p.c.s. value. Similarly, all aliphatic RNH_2_ complexes are assumed to have the same value (0.07 mm s^−1^). After Bancroft et al. [73] we assume agreement between observed and calculated data within ± 0.05 mm s^−1^ is satisfactory and we have no results outside this range that we do not discuss in detail.

Bancroft et al. [73] have demonstrated that the p.c.s. values give an estimate of the σ and π bonding properties of ligands. Whilst these properties are difficult to extract from the p.c.s. data, the values have been found to decrease with an increase in σ-bonding and π-back bonding [73].

**Fig. 1:**
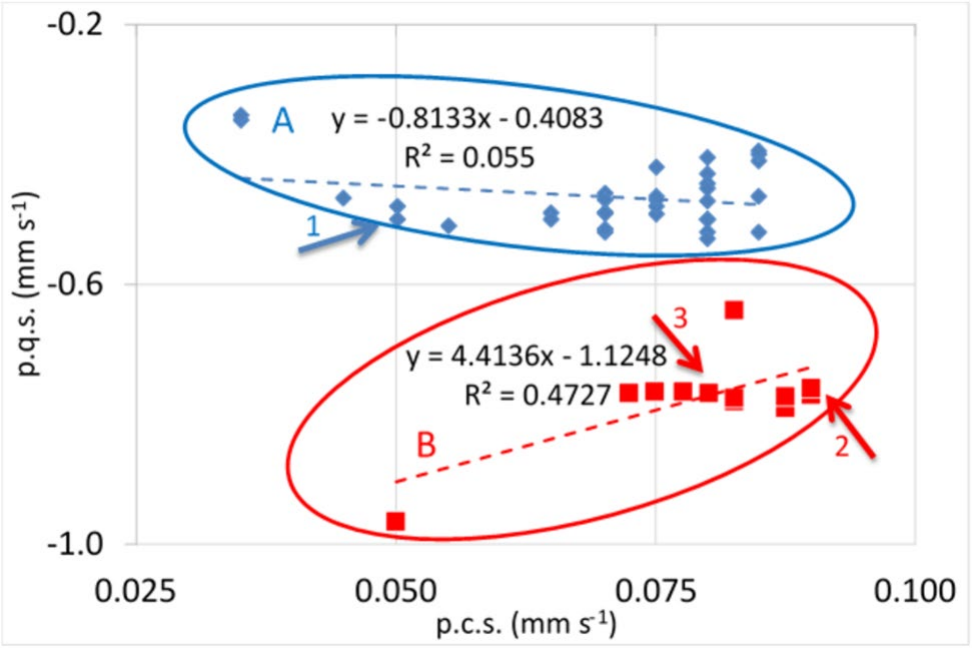
Plot of calculated p.c.s. values against calculated p.q.s. values for ligands bound to the octahedral low-spin iron(II)centre in [Fe(Macrocycle)L_2_] complexes, where L = a nitrogen bonding ligand. Two classes of ligands can be distinguished: **A** ligands bound to the Fe(PPIX) group and **B** various macrocycle nitrogen ligands (for the macrocycle ligands only the value of one of the four equivalent central N atoms is used). Arrow 1 points to histidine. Arrow 2 points to PPIX. Arrow 3 shows the position of TPPS. This figure has been constructed from the data presented in Table 6. Trend lines for A and B complexes are indicated; the correlation coefficient for A is very low, indicating that the negative slope of the trend line is not particularly reliable

Figure 1 presents a plot of calculated p.c.s. values against calculated p.q.s. values for ligands bound to the octahedral low-spin iron(II) centre in [Fe(Macrocycle)L_2_] complexes, where L = a nitrogen bonding ligand. (The p.q.s. values are considered in full in the next section in the paper). The first significant observation is that there are two different groups of values viz:- those in blue which are values for axial ligands, whereas the second group are those of the macrocyclic ligands. This figure then illustrates how the properties of the two types of nitrogen ligands vary. The two trend lines presented have slopes of opposite sign, that for the axial ligands (the blue line) has a poor R^2^ value and is not therefore reliable in indicating a significant trend and thus does not give us further insight into the properties of the axial ligands; we will discuss their properties later. The slope of the red line is more significant and indicates a graduation of the properties of the macrocyclic ligands going from left to right indicating a continuous change in their bonding properties. Thus, from Tables 1, 2, 3 and 4 (and Fig. 1) the following points can be made: phthalocyanine (pc) has the smallest p.c.s. value. This ligand is usually thought of as being a very strong σ donor and hence the result is in keeping with the accepted understanding [94, 102]. As will be seen, this ligand is the poorest π-acceptor of the macrocycles examined. The low-p.c.s. value arises predominantly from its good σ-donating properties. It is well known that the central hole in the pc ring is smaller than that of the porphyrins, so the nitrogen atoms on the inside of the pc are closer to the iron at the centre (see for example Ref. [102]) consistent with the properties described above.

The porphyrins all have p.c.s. values larger than the monodentate nitrogen ligands (when all four N_por_ ligands are added together (see values in Tables 6 and 7), probably in part due to the fact that they are held in a ring at an almost fixed distance from the iron centre [19]. If the iron(II) is high spin and in the porphyrin plane, core expansion can take place [19, 119]. The fixed distance suggests that σ-donating and π-acceptor properties of the porphyrins do not vary very much and only octa-methyl tetra-benzo porphyrin (OMTBP) differs from the others significantly. It is apparent from Fig. 1 that OMBTP is the weakest σ-donor ligand in bonding though it is said to be a good π-donor [99]. This suggests that the other porphyrins are better σ-donors and better π-acceptors than OMTBP. From Fig. 1 it is apparent that TPPS is the best π-donor of the porphyrins.

All the other monodentate ligands N-ligands have p.c.s. values ranging from between ~ 0.05 and 0.1 mm s^−1^. The aliphatic ligands are pure σ-donors and have values for the most part around 0.07 mm s^−1^. The other ligands such as the imidazoles and pyridines have values depending on how substituents change the bonding properties of the bonding nitrogen atom or cause steric hindrance to the bonding. The most significantly sterically hindered σ-bonding ligands such as tertiary-butylamine pip, morph and 2, 6- dimethyl-morphine have the largest p.c.s. values of the monodentate nitrogen ligands. In the case of the piperidine complex, the question may be asked “is it really sterically hindered?” as it still is on the trend line presented in Fig. 3 of reference 40 for the non-sterically hindered aliphatic ligands. The answer is yes as though it is aliphatic its steric hindrance leads to it being a much weaker ligand than the other aliphatic ligands and thus it causes a larger *ΔE*_*Q*_.

It appears therefore that by linking four pyrrole rings together into a porphyrin, the resulting p.c.s. values are larger than single nitrogen ligands and the σ-bonding and π-back bonding properties taken together are weaker. However, we will return to this point in detail later in this paper.

It is worth noting that p.c.s, values for other macrocycles have been reported [77, 78]. Only one tetraan-hydroaminobenzaldehyde (TAAB) Ref. [77] is structurally even remotely related and of close enough symmetry. The p.c.s. value for TAAB is 4 × 0.07 mm s^−1^ placing it between pc and the porphyrins in its bonding properties. We note that the p.q.s. value reported for TAAB of 4×− 0.62 mm s^−1^ (Ref. [77]) makes it smaller than OMBTP, see later in this work.

The [Fe(Por)(CO)L] complexes all represent a problem as all of these have smaller i.s. values by around 0.2 mm s^−1^ compared to the corresponding [Fe(Por)L_2_] complexes. This means that deriving p.c.s. values for the porphyrins in these CO complexes will not be simple; and they will result in different values to those of the [Fe(Por)L_2_] complexes. Obviously any p.c.s. values derived will be very different as the addition of the CO completely changes the bonding not only on the other axial ligand but obviously on the porphyrin itself. To derive a new set of p.c.s. values for the [Fe(Por)(CO)L] complexes a first approximation may be made that the remaining axial nitrogenous ligands, which are only σ-bonding, will not change their bonding to the Fe when the CO is the other ligand. This would generate the value for the porphyrin and the CO combined. However, if the p.c.s. value of CO is taken from the literature [73] (given therein as 0.0 mm s^−1^) then the p.c.s. value for the porphyrin in the complex can be estimated. When this is applied to the data in Table 5, it is apparent that the resulting values for the porphyrins are much lower than their values found in the [Fe(Por)L_2_] complexes: this is evidence that there is a redistribution of electron density on the porphyrins when CO displaces one or indeed both axial ligands. It is significant that the four [Fe(Por)(CO)_2_] complexes in Table 5 yield p.c.s. values for the porphyrins that are greater than those of the [Fe(TPP)(CO)L] complexes, and in the case of [Fe(OMBTP)(CO)_2_] the value is very close to that derived for the [Fe(OMBTP)L_2_] complexes. The other three [Fe(Por)(CO)_2_] complexes yield porphyrin p.c.s. values between those derived from the [Fe(Por)L_2_] and those from the [Fe(Por)(CO)L] complexes. This is evidence that the p.c.s. value of 0.0 mm s^−1^ for CO assumed from previous work [73] is verified herein. The *δ* values for the [Fe(Por)(CO)_2_] complexes (where Por = OEP, TPP, OMTBP or Pc) are similar too or slightly larger than those of their related [Fe(Por)(CO)L] complexes. From this and the fact that the p.c.s. value of CO is 0.0 mm s^−1^ led to initial calculations herein for *δ*_Calc._ values based on the Por p.c.s. values (used for the [Fe(Por)(CO)L] compounds) being too small for the [Fe(Por)(CO)_2_]. This indicated that they were all anomalous and require different p.c.s. values for the Por ligands in the [Fe(Por)(CO)L] compounds. We will return to and discuss this further in the section on p.q.s. values.

Connor et al. [110] presented an explanation for the decrease in the *δ* in the [Fe(TPP)(CO)L] compounds. However, Sams and Tsin [102] questioned the argument as stronger π-bonding between Fe and CO will decrease the population of the electron density on the Fe 3d_xz_, 3d_yz_, orbitals, which would increase *δ* as observed, but should increase rather than decrease *ΔE*_*Q*_. In addition, weaker axial ligand to Fe σ-bonding should decrease the proportion of Fe electron character in the bonding orbitals arising from the Fe 3d_z_^2^, 3p_z_, and 4 s orbitals; we are not sure of the effect this would have on *δ*, however, it would be. expected to increase *ΔE*_*Q*_. Sams and Tsin [102] correctly state that these types of argument tacitly assume that the makeup of the Fe-porphyrin bonds are not affected by changes in the axial ligands. Our observations on the changing p.c.s. values for the porphyrins in the [Fe(Por)L_2_] and [Fe(Por)(CO)L] complexes clearly support their argument. Sams and Tsin [102] further suggest that it seems necessary to assume that porphyrins have a pronounced “electron sink” capability enabling them to modify their σ and π bonding to meet the needs of the axial ligands; explaining that this behaviour/ability may in part be responsible for the diverse functions in which metallo-porphyrins take part in biological systems. Obviously form the finding that the p.c.s values of the porphyrin can change when the axial ligands make demands on the Fe(II) atom for additional π bonding, means that we would expect to have to modify the p.c.s. value for the porphyrin in the presence of any axial ligand that has such requirements.

### Quadrupole splittings

Before turning to the p.q.s. data, we note that the sign of V_zz_ (where V_zz_ is the principal direction of the electronic field gradient and has been derived in Ref. [102]) is positive and the asymmetry parameter η is nearly or exactly zero, for all the low-spin porphyrin [Fe(II)(Por)(L)_2_] and pc iron complexes [Fe(II)(pc)(L)_2_] where L = a nitrogenous (aliphatic or aromatic) ligand considered in this work [90, 97, 99, 102]. Such small η values are in keeping with *D*_4h_ symmetry at the iron site [120]. In these diamagnetic low-spin [Fe(II)(Por)(L)_2_] complexes the major contribution to V_zz_ arises from an imbalance in electron densities in the *d*_x_^2^_-y_^2^ and *d*_z_^2^ orbitals [102, 121]. Thus the observed positive signs shows the covalent bonding from the porphyrin to the Fe atom is stronger than that of the axial ligands (In other words, the charge is concentrated in the xy plane). The reverse is true when the sign is negative (as will be discussed later herein).

### p.q.s. data

All the p.q.s. data in Tables 6 and 7 were calculated by first assuming the value for imidazole (− 0.46 mm s^−1^) from Dabrowiak et al. [77] and then amending this value (to − 0.52 mm s^−1^) from the data used in this work in Tables 1, 2, 3, 4 and 5.

The first important observation of the data derived in this work is that all the monodentate nitrogen ligands have p.q.s. values in the range − 0.395 to − 0.53 mm s^−1^. The exact value a given nitrogen ligand has will depend on its inherent properties such as:-

- whether it is only a σ-bonding ligand;
- whether it is sterically hindered;
- whether it is both σ- and π-bonding.

Thus, purely σ-bonding ligands that are non-sterically hindered have p.q.s. values around − 0.49 to − 0.52 mm s^−1^. For those that are very sterically hindered such as piperidine, the value drops to -0.40 mm s^−1^.

For ligands that are σ-donors and poor π-acceptors (which are not sterically hindered), such as imidazole p.q.s. values around − 0.50 to − 0.53 mm s^−1^ are found. The pyridines which are σ-donors and better π-acceptors than the imidazoles have p.q.s. values around − 0.46 to − 0.48 mm s^−1^.

All the tetradentate macrocycles have p.q.s. values that are significantly more negative than the monodentate ligands. The p.q.s. values increase from pc (single metal-bonding nitrogen − 0.965 mm s^−1^). This is in keeping with the p.c.s. data in that pc is the best σ-donor ligand and the porphyrins (especially OMTBP) are better π-back-bonding ligands. As there are four nitrogen atoms bonding the iron from the macrocycle, we have chosen to consider the sum of all four as the macrocyclic contribution; hence, for pc the total p.q.s. value becomes − 3.86 mm s^−1^. As the pc ring houses a smaller central hole than the porphyrins (see bond length section below) the central nitrogen atoms will be closer to the low-spin iron(II) atom than in similar porphyrin compounds. Thus, by forming a macrocycle it is possible to force N atoms close to low-spin iron(II) and the result is strong σ-bonding. All the porphyrins are less strong σ-bonding ligands than pc, but they are better π-acceptors. To understand the bonding in these porphyrin ligands, it is necessary to try to separate σ and π bonding effects. This has been shown to be possible by Bancroft et al. [73, 75] remembering the proportionalities that [73]:

$$\begin{gathered} {\text{p.q.s.}}\,\infty-q_{\text{lattice +}} (\pi\text{'}-\sigma\text{'}) \hfill \\ {\text{andp.c.s}} \infty-(\sigma_{+}\pi), \hfill \\ \end{gathered}$$

where *q*_lattice_ is the field gradient due to external charges, π’ represents the π-bonding affecting the p.q.s. value, σ’ is the σ-bonding affecting the p.q.s. value, π is the π-bonding affecting the p.c.s. value and σ is σ-bonding affecting the p.c.s. value. (π’–σ’) is then a measure of the field gradient acting on the Fe(II) for a given ligand when q_lattice_ can be ignored, and (σ _+_ π) for a given ligand is a measure of the “s” electron density at the nucleus due to σ donation (s electron density) and π-donation (p and d electron shielding the nucleus from s electrons) caused by the ligand.

Bancroft et al. [73] have shown that p.q.s. values become more positive with increasing π-back-bonding and more negative with increasing σ-bonding and increasing *q*_lattice_. A plot of p.c.s. values against p.q.s. values can be prepared [75, 77] for neutral ligands. As a plot of—(σ _+_ π) against (π–σ) (as *q*_lattice_ can be neglected for neutral ligands), then a slope in the positive sense indicates σ-dominance in the bonding, whilst a negative slope means π-dominance [75]. Bancroft et al. [74] further point out that there is no a priori reason to expect linear correlations so there is no reason to expect correlations in Fig. 1, however, inspection of the figure shows that a line through pc and OMTBP would pass close to the porphyrins and be of positive slope. So, all the macrocycles manifest a dominance of σ-bonding.

First, it is important to realise that the Por macrocycles are all 2^−^ (charged) ligands (not neutral). Thus, for these ligands there will be a lattice effect, however, as such an effect is usually smaller than 10% of the quadruple splitting, we chose to ignore it. In addition, all the compounds for the same macrocycles should be affected to the same extent.

From the Tables 1, 2, 3, 4 and 5 and Fig. 1 of the p.q.s. values several statements can be made:

- The absolute p.q.s. values of the porphyrin macrocycles are all generally more negative than the monodentate nitrogen ligands showing perhaps only slightly more π-bonding dominance in their bonding. In the plot (Fig. 1) the porphyrin macrocycles (all within the bottom ellipse) are plotted as single ligating entities, in fact four of them surround the iron atom in the porphyrin plain. This fact is important and discussed in (c) below. The Pc ligand is below the porphyrins to the left. OMTBP is at the top right of the circle. The picket fence porphyrin lies on the left and has less effect on the *δ* compared to the central group of seven porphyrins. All the mono-nitrogen bonding ligands have p.q.s. values less than − 0.55. The two most sterically hindered ligands have the lowest p.q.s. values and the lowest p.c.s. values.
- Histidine (indicated by the arrow in Fig. 1), the ligand most similar to the common axial ligand found in many haem proteins, is significantly different from the other monodentate nitrogenous ligands. Its closest neighbours in Fig. 1 are the sterically hindered ligands 2-methyl piperidine and 5-chloro,1-methylimidazole.
- The differences between the porphyrin macrocycles can be best appreciated when the p.q.s. values are taken as a sum for all four macrocyclic bonding nitrogen atoms as this is the field the macrocycle as an entity is subjecting/imposing on the iron atom (see Fig. 1). Clearly when this is done, the differences between the different porphyrins can be appreciated. It is apparent that none of the non-naturally occurring porphyrins are applying the same field as PPIX (see arrow 2 pointing to PPIX in Fig. 1). This emphasises the uniqueness in the chemistry of PPIX and helps us realise that it is this unique field that is ubiquitous in haem proteins. Interestingly, in support of this finding others have noticed that in a range of [Fe(III)(Por)(Im)_2_]Cl (where Por = PPIX, PPD, TPP, PMXPP or PClPP) the *Δ*E_Q_ appears to be dependent on the nature of the porphyrin substituents [102]. It is apparent that the contribution of the porphyrin to the EFG will dominate most of these low-spin six-co-ordinate Fe(II) complexes where the sign of the field gradient is positive; this will only change in the presence of very strongly bonding axial ligands and then the sign of the field gradient will reverse.
- The porphyrins are all mainly to the right of the monodentate nitrogen ligands (in Fig. 1) indicating that they are better σ-donating ligands, and from the comments referring to line slope above, this is the dominating force in the bonding.
- Also, in Fig. 1 ¼pc is lower than the monodentate nitrogen ligands showing it is a much worse π-bonding ligand (than the aromatic ligands), but it is a much better σ-donor than either the aliphatic or aromatic ligands. As discussed earlier, presumably, this is because its N atoms are forced very close to the iron atom. [It would have been thought that this will also help π-bonding, since the *d*_xz_ and *d*_yz_ orbitals of Fe are (substantially) overlapping the 2pz orbital of the macrocyclic N atoms, however for pc the extended delocalisation of this macrocycle into its benzene rings must weaken the π-bonding to the Fe.
- Apart from OMTBP the other porphyrins are in a close group showing that their bonding properties are similar but not the same. For the OEP complexes there are few data to base the value of OEP on and for one of these the *Δ*_calc_ is 0.20 mm s^−1^ different to *Δ*_obs_ (see Table 3). TPPS has the closest p.q.s. value to TPP and is thus better π-donating than PPIX and this is likely to be why its’ chemistry when bound to Fe(II) is different to the latter in aqueous solution [24, 29, 41, 63].

**Fig. 2:**
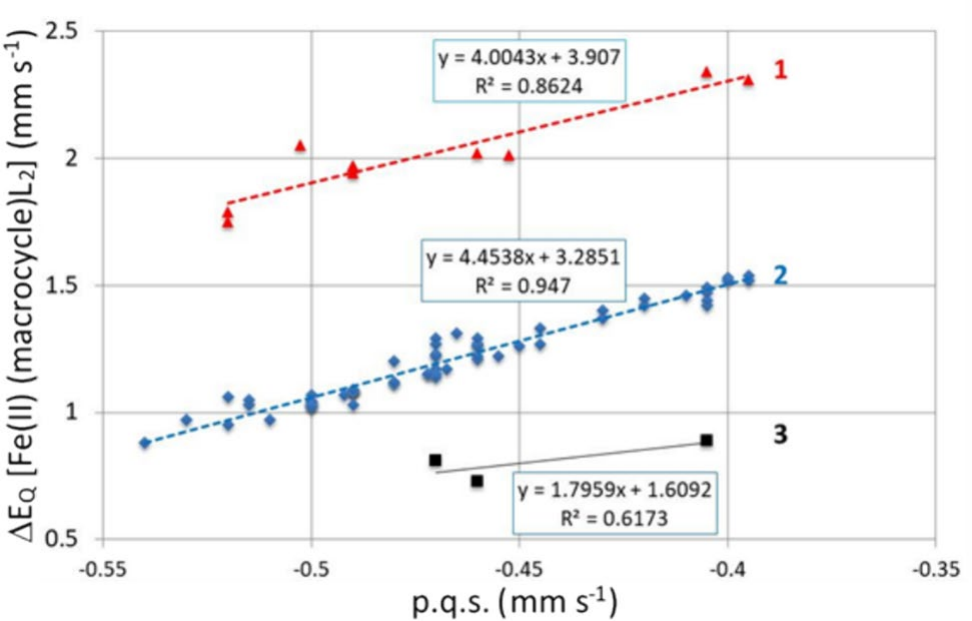
Plot of *Δ*E_Q_ versus calculated p.q.s. for the [Fe(II(Por))L_2_] complexes (where L represents a nitrogenous ligand). Trend lines:- 1 passes through the [Fe(II(Pc))L_2_] complexes; 2 passes through the [Fe(II)(Por)L_2_] complexes; 3 passes through the [Fe(II)(OMBTP))L_2_] complexes

Figure 2 presents a plot of *ΔE*_*Q*_ versus calculated p.q.s. for many of the [Fe(II)(Por)L_2_] complexes (where L represents a nitrogenous ligand) that appear in Tables 1, 2, 3 and 4.

We presented Eq. 2 in the theory section on the point charge model. For calculating *Δ*E_Q_ from p.q.s. values of the various ligands, we reproduce it here in a generalised form to aid in discussing its’ implications:

4 $$\Delta {E}_{Q}= a \text{ p.q.s. }\left(L\right)+ b \text{ p.q.s. }\left(\text{macrocycle}\right),$$

where the coefficients a and b are 4 and − 4 in Eq. (2), whilst L is a nitrogenous ligand.

If the macrocycle is constant, say a porphyrin for a series of [Fe(II)(Por)L_2_] complexes where L can vary, then the second term in Eq. (4) is a Constant and Eq. (4) becomes:

5 $$\Delta {E}_{Q}=a \text{ p.q.s. }\left(L\right)+\text{Constant}.$$

From this equation it follows that *ΔE*_*Q*_ of a series of porphyrin complexes with varying L depends only on the value of p.q.s.(L). This is apparent in Fig. 2. For other macrocycles, the Constant will be different, establishing parallel lines, because the coefficient *a* is the same for a set of L ligands with a given macrocycle. The fact that the correlation coefficients are close to one vindicates our finding that the EFG the Fe(II) atom experiences from a given porphyrin is invariant and the changes in the field gradient that cause changes in the *ΔE*_*Q*_ values are due to the changes (in bonding) in the axial ligands. If the macrocycle is changing for a given ligand L, we get corresponding behaviour, however, the slope of the line is *b*. According to Eq. (2) b is − 4, which means that *ΔE*_*Q*_ decreases when the p.q.s. value of the macrocycle increases.

From this simple analysis of Eq. (2) it would be expected that lines with a slope of 4 will be generated. From Fig. 2 it is apparent that the complexes divide into three groups.

The group referred to as 1 is that of [Fe(II)(Pc)L_2_] complexes and their data give a reasonable fit to a straight line. The group labelled 2 is that of most of the [Por(II)L_2_] complexes other than those of the [Fe(II)(OMBTP)L_2_] complexes (which are labelled 3 in the plot). This plot nicely illustrates how our findings from the p.c.s. discussion on the porphyrins hold true for the p.q.s. values as well in that it shows how the nearly fixed distances of the four porphyrin nitrogen atoms to the Fe(II) centre suggests that σ-donating and π-acceptor properties of the porphyrins do not vary very much and only OMTBP differs from the others significantly. It is also apparent that the bonding in the [Fe(II)(Pc)L_2_] due to the Pc macrocycle is very different to the porphyrins.

**Fig. 3:**
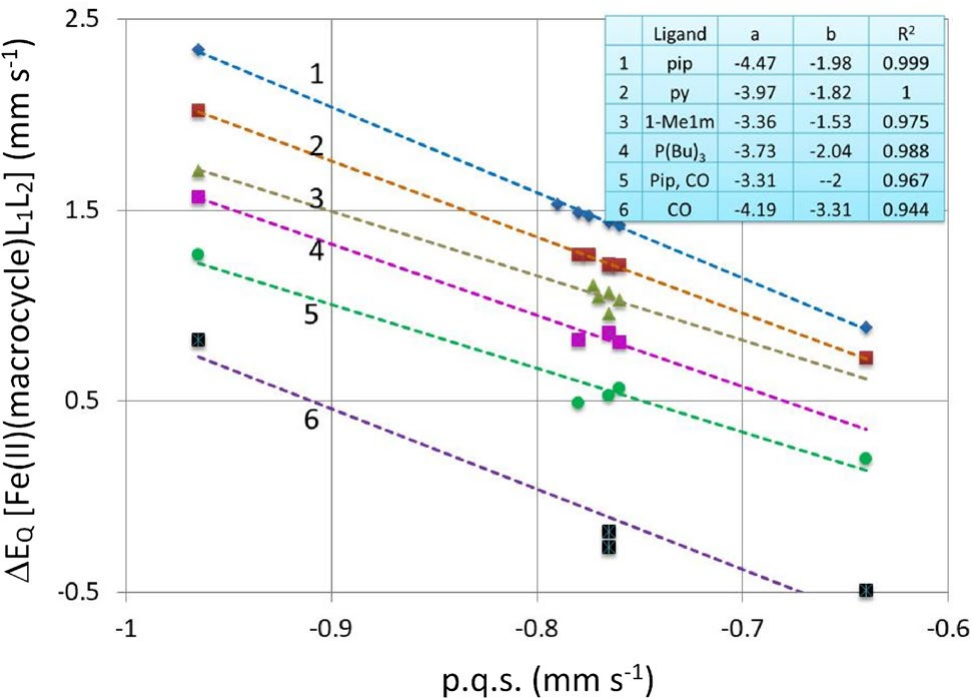
Plot of *ΔE*_Q_ versus calculated p.q.s. for the [Fe(II)(Por)L1L2] complexes (where L1 and L2 represent axial nitrogenous ligands, which are in most cases identical). Trend lines for the various ligands are indicated by numbers. 1: L1 = L2 = pip; 2: L1 = L2 = py; 3: L1 = L2 = 1-Me1m; 4: L1 = L2 = P(Bu)_3_; 5: L1 = pip, L2 = CO; 6: L1 = L2 = CO. The insert shows the coefficients of the trend lines for the various ligand groups, the correlation coefficients are > 0.95

In Fig. 3 we have plotted *ΔE*_*Q*_ versus p.q.s for Fe(II) porphyrin complexes with constant axial ligands and varying macrocycles. According to Eq. (2) we expect a negative slope of the trend lines and this is what we see in Fig. 3. We were restricted in the plot by the number of known complexes of each axial ligand with these macrocycles. For pyridine there are seven-known complexes and therefore seven points on the pyridine line. Likewise, there are seven points on the piperidine line, whereas for the other four lines there are less points as there are fewer known complexes.

Figure 3 shows the slope of the trend lines of the various ligands (whilst the macrocycles are considered to manifest the same values in the presence of all the ligands) is about − 4, as indicated in Eq. (2). We think that the results presented in Figs. 2 and 3 are a nice illustration of the algorithm indicated by Eq. (2). As in Fig. 2, the results displayed in Fig. 3 vindicate the approach.

**Fig. 4:**
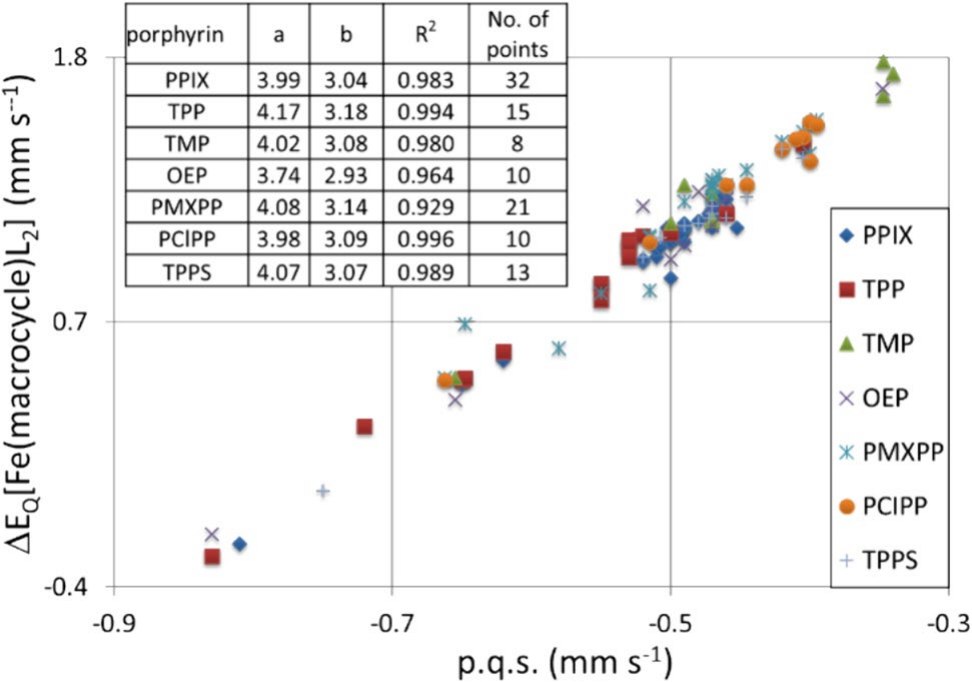
Plot of *ΔE*_Q_ versus calculated p.q.s. for the [Fe(II)(Por)L_2_] complexes (where L represents a nitrogenous ligand, a P-bonding ligand or CO; Por = PPIX, TPP, TMP, OEP, PMXPP, PClPP and TPPS. Trend lines are not presented for reasons of clarity. The formula for the trend lines is *ΔE*_Q_ = a p.q.s. + b. The correlation coefficients R^2^ (very close to 1) for the various trend lines are also presented in the inserted Table

To enable further information to be extracted from group 2 (in Fig. 2) the [Fe(II)(Por)L_2_] complexes and to see any differences between the different porphyrins Fig. 4 has been constructed. This figure presents a further plot of *ΔE*_*Q*_ versus calculated p.q.s. for the [Fe(II)(Por)L_2_] complexes, but this is extended, so L now represents a nitrogenous ligand, a P-bonding ligand or CO, and Por = PPIX, TPP, TMP, OEP, PMXPP, PClPP and TPPS. In the figure-trend lines for these seven porphyrins are not shown for reasons of clarity but the values of a, b and R^2^ are given for each porphyrin in the Table within the figure. The formula for the trend lines is *ΔE*_*Q*_ = a p.q.s. + b. The correlation coefficients R^2^ for each of the different [Fe(II)(Por)L_2_] complexes trend lines are very close to 1 for each of the various porphyrins. As each line is different and nearly parallel, this indicates that the bonding properties of each porphyrin are only subtly different.

Unfortunately, we could not plot the [Fe(II)(Por)(CO)L] complexes in Fig. 4 as the two axial ligands are different. Nevertheless, we choose to discuss them at this point before returning to Fig. 4. In contrast to the finding that the porphyrin p.c.s. values change when CO replaces an axial ligand that we discussed earlier there appears to be no associated change in the porphyrin p.q.s. values. However, in fitting the observed *ΔE*_*Q*_ values for the [Fe(Por)(CO)L] and [Fe(Por)(CO)_2_] it is apparent that the CO ligand bonding depends primarily on the bonding of the other axial ligand and if/how this is modified when the CO is present. Some further pertinent points are now discussed:-

- The p.q.s. value of − 0.80 mm s^−1^ used as a first approximation for CO was not good for all the [Fe(II)(porphyrin)] complexes containing one or more CO-axial ligands. Indeed, values between − 0.74 mm s^−1^ and − 0.88 mm s^−1^ needed to be used depending on the nature of the macrocycle (all have different p.q.s. values) and also importantly of the 5th ligand (see Table 7). This is perhaps not surprising as CO is well known to be a strong π-back bonding ligand. For [Fe(II)(Por)(CO)L] complexes such π-back-bonding may cause the other axial ligand bonding to strengthen if it has a π-bonding orbital involved in its bonding (however this does not mean its p.q.s. value will change). Now that we have explained the derivation of the p.q.s. values for the CO ligands with the different porphyrins, we can return to a statement we made earlier. We stated that we could not plot the [Fe(II)(Por)(CO)L] complexes in Fig. 4 as they have two different axial ligands and hence two different p.q.s. values, though this is true if we take the average p.q.s. value of the two ligands we can plot this and then we can see that the complexes do indeed fall on the same lines. This again shows the axial ligands dominate the changes in the *ΔE*_*Q*_ values and that it is the combined effect of the two axial ligands that dominates the change in the field gradient in the six-co-ordinate low-spin [Fe(II)(Por)L_2_] and [Fe(II)(Por)(CO)L] complexes.
- For the [Fe(II)(por)(CO)_2_] complexes all four complexes listed in Table 5 are shown to have negative *ΔE*_*Q*calc_ values using these calculations. Indeed, the only macrocycle bis-carbonyl complex that has a positive *ΔE*_*Q*calc_ is [Fe(II)(pc)(CO)_2_] (see Table 5). In the case of the latter complex though the *ΔE*_*Q*calc_ is positive in agreement with the experimental finding, its value is very much less than the experimental [118]. It should be noted that the *ΔE*_*Q*calc_ values of all the [Fe(Pc)(CO)L] complexes fit the experimental values quite well so for these complexes a p.q.s. value of − 0.90 mm s^−1^ works well. It may be that p.q.s. value generated by the CO in this complex is closer to those of the porphyrins as Pc cannot give enough π-bonding to the Fe(II) to fully offset the requirements of two axial CO ligands. The finding that all three [Fe(II)(Por)(CO)_2_] manifest negative *ΔE*_*Q*calc_ values yet only the OMBTP complex has a measured negative *ΔE*_*Q*obs_ is an indication of the power of these simple point charge calculations. Clearly assigning positive values for the TPP and PPIX complexes would cause these points to be off the trend lines for these porphyrins.
- Returning now to Fig. 4, in several cases the ligand p.q.s. values given in Table 7 are used for two or more different [Fe(II)(Por)L_2_] complexes in Fig. 4. This is clear evidence that a simple point charge model works well when the two axial ligands are the same. As the complexes in Fig. 4 contain data for three classes of ligand (N-type, P-type and CO) and are in all seven cases good straight lines then a further deduction can be made. The fact that three of the [Fe(II)(Por)L_2_] complexes where L = CO (Por = PPIX, TPP and OEP) fit onto their trend lines well is evidence that the negative value calculated for their *ΔE*_*Q*_ values is correct as (individual) positive values would not have fitted the trend lines well.

**Fig. 5:**
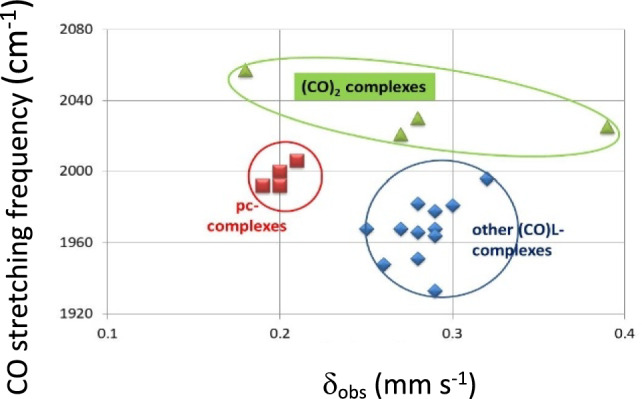
CO-stretching frequency of [Fe(P)(CO)L] and [Fe(P)(CO)_2_] -complexes versus the observed isomeric shifts *δ*. Three classes of complexes can be distinguished: the complexes with two CO-groups, the pc-complexes with one CO and one nitrogenous ligand and the other (CO)L complexes listed in Table 5

In Table 5, the carbonyl stretching frequencies are listed. It is apparent that even for carbonyls bonding to the same iron porphyrin with different opposite axial ligands there is significant variation in the asymmetric stretching frequencies; this indicates differences in the cooperative bonding across the iron atom depending on the nature of the axial ligand. This agrees with the observed variability of the p.c.s. values for the CO molecule and in addition the values satisfying the bis-carbonyl derivatives again vary. Of course, the contribution to the value of the carbonyl p.q.s. value also differs from one macrocycle to the next although this appears to have a smaller effect than the axial ligand. In Fig. 5 the carbonyl-stretching frequencies are plotted against the *δ*_obs_ mm s^−1^. The first striking point is the rather large difference between the carbonyl-stretching frequencies of the (CO)_2_ complexes and the (CO)L complexes.

Three of the four [Fe(Por)(CO)_2_] complexes clearly lie directly above the related [Fe(II)(Por)(CO)L] complexes indicating that the macrocycle directly contributes to the observed frequencies. The fact that all the [Fe(II)(Por)(CO)L] complexes occur in the same area in the figure and are well separated from the [Fe(Pc)(II)(CO)L] complexes is in keeping with the discussion herein about the porphyrin complexes having better π-bonding to the Fe atom. Unfortunately, there are no carbonyl-stretching frequencies available in the literature for the two [Fe(II)(OMTBP)(CO)L] complexes listed in Table 5, however from their known *δ*_obs_ values these complexes would lie to the right of most of the other [Fe(II)(Por)(CO)L] complexes though they might not be far enough to the right to lie directly under the Fe(II)(OMTBP)(CO)_2_] complex. This is a surprising result and as the Mössbauer data for the [Fe(II)(OMTBP)(CO)L] and the [Fe(II)(OMTBP)(CO)_2_] complexes come from the same laboratory, it would be expected that there is nothing wrong with the data [82, 102, 117]. Hence, the explanation for the larger *δ*_obs_ for the latter complex must be something to do with the bonding orbitals. In fact, as we discussed earlier in the paper, the results of our calculations on the p.c.s. values for all the [Fe(Por)(CO)_2_] compounds suggest that they are all surprising large not just that of the OMBTP complex. An explanation suggested by Reimer et al. [117]:- “For the [Fe(II)(Pc)L_2_] and [Fe(II)(TPP)L_2_] complexes as CO replaces the other axial ligands the changes reduce the electronic anisotropy of the iron valence orbitals whilst maintaining an oblate ( +) charge whereas for [Fe(II)(OMBTP)L_2_] the charge switches to prolate (-). This reflects the weaker ligand field provided by OMTBP than by TPP and Pc.” must be questioned in the light of our findings on the p.c.s. values that indicate that the other [Fe(II)(Por)(CO)_2_] complexes also have prolate charge distributions except for Pc.

A further part of the discussion of results provided by Reimer et al. [117] relates to the low υ_CO_ cm^−1^ value observed for [Fe(OMTBP)(CO)_2_]. They suggest this indicates a greater degree of Fe to CO π-electron transfer, which to be consistent with the large negative shift in V_zz_ (they observe) would require stronger (i.e. shorter) Fe–C(O) bonds and a higher degree of localization of π-density. Though this may well be true from Fig. 5 it is apparent that the υ_CO_ cm^−1^ values observed for all the [Fe(II)(Por)(CO)_2_] complexes are equally raised from those of their related [Fe(II)(Por)(CO)L] complexes.

We do however fully agree with Reimer et al. [117] that OMTBP appears to be the most effective “electron sink”. The data for [Fe(II)(OEP)(CO)_2_] and for [Fe(II)(PPIX)(CO)_2_] (see Table 5), supports these arguments. The calculations of the *ΔE*_*Q*calc_ presented herein would suggest that the porphyrins TPP, OEP and PPIX form [Fe(II)(Por)(CO)_2_] complexes with prolate charge distributions like that of [Fe(II)(OMTBP)(CO)_2_]. We note that Reimer et al. [117] found [Fe(II)(TPP)(CO)_2_] to have a prolate charge distribution, but others have stated that it is very difficult to measure the sign of V_zz_ accurately when the *ΔE*_*Q*_ value is small [12, 123] hence the graphical method presented herein appears to be a better way of assigning the sign of V_zz_.

At this point it should be remembered that we have not yet explained the large *ΔE*_*Q*_ values that appear in Table 3 for some of the porphyrin complexes containing 2-methylimidazole. In addition, we have been able to produce [Fe(II)(PPIX)(2-MeIm)_2_] complexes containing this sterically impeded ligand that show more normal *ΔE*_*Q*_ values see Table 3. However, in our complexes the H^+^ on the second N of the imidazole ring was partially or completely removed. In the complexes containing 2-MeHIm where the larger *ΔE*_*Q*_ values are observed the H^+^ is present on the ligand and this makes it a much weaker bonding ligand, its p.q.s. value is − 0.348 mm s^−**1**^. The [Fe(II)(TPP)(1,2-Me_2_Im)_2_] and [Fe(II)(TPP)(1,2-Me_2_Im)(CO)] complexes both show larger *ΔE*_*Q*_ values than those with less sterically hindered ligands: the p.q.s. value for 1,2-Me_2_Im is − 0.34 mm s^−1^.

It is worth digressing to Table 8 at this point. The complexes presented in Table 8 were originally used to model P-450_Cam_ [27, 66, 124]. Herein we used these data to derive p.c.s. and p.q.s. values for the sulfonated ligands bound to [Fe(II)(PPIX)]. In frozen solution in the absence of CO they all form five co-ordinate high-spin [Fe(II)(PPIX)L] complexes, but bubbling CO into the solutions and refreezing generates the corresponding low-spin [Fe(II)(PPIX)(CO)L’’] six-coordinate complex [27, 66] (where L’’ = a sulfonated ligand). These six-coordinate complexes all manifest *δ* values in the range 0.30 to 0.37 mm s^−1^ at 78 K (those are smaller than those in tables 4 for the [Fe(II)(PPIX)(CO)L] complexes with nitrogenous ligand L and *ΔE*_*Q*_ values in the range 0.6 to 0.95 mm s^−1^. The *ΔE*_*Q*_ values yield p.q.s. values in the range − 0.335 to − 0.23 mm s^−1^ [27, 66] except for that of ethyl 2-mercaptoacetate which has a calculated p.q.s. of − 0.40 mm s^−1^ which is high (we note without the CO ligand only the high-spin five-coordinate [Fe(II)(PPIX)] complex forms). For the two [Fe(II)(TPPS)(CO)L] complexes the calculated p.q.s. values also are high (− 0.465 mm s^−1^ and − 0.475 mm s^−1^) and are different from those of the equivalent PPIX complexes. Clearly something is happening in the bonding on the [Fe(TPPS)(CO)L] complexes. We note that a similar *Δ*E_Q_ value was reported by others [124] for a sulfonated ligand that also only forms a six-co-ordinated low-spin complex with CO (see data in Table 8 where we have calculated a p.q.s. value of − 0.43 mm s^−1^). Thus, for the four complexes of the type [Fe(II)(Por)(CO)L] [27, 66, 124] which apparently have p.q.s. values that are more like nitrogenous ligands the environment around the S-bonding ligand is affecting the contribution the ligand is making to the electric field across the Fe(II) atom. It should be noted that all four complexes have typical hyperporphyrin spectra with the Soret band between 336.5 and 451 nm. However, in all cases the four ligands do not form low-spin [Fe(Por)L_2_] complexes [27, 66, 124]. For the four cases where the p.q.s. values are between − 0.335 and − 0.23 mm s^−1^ low-spin complexes of the type [Fe(II)(PPIX)L_2_] also do not form [27, 66]. However, if they did, then if we take the value range of − 0.335 to − 0.23 mm s^−1^ with [Fe(II)(PPIX)] we would expect *ΔE*_*Q*_ values in the range ~ 1.70 to 2.12 mm s^−1^. Such values are larger than anything yet found, hence we would rule out the possibility of finding six-co-ordinate low-spin [Fe(II)(PPIX)L_2_] complexes with such values. This we believe is a very significant finding as this sets a limit to the *ΔE*_*Q*_ value range for low-spin complexes of the type [Fe(II)(PPIX)L_2_] and we will return to this latter in this paper. Thus, these sulfonated ligands cannot form low-spin [Fe(II)(PPIX)L_2_] complexes, but when CO is present as the other axial ligand, they can form [Fe(II)(PPIX)(CO)L] complexes [27, 66, 124]. It is noteworthy that [Fe(TMP)(1,2-Me_2_Im)_2_] and [Fe(TMP)(2-MeHIm)_2_] have *ΔE*_*Q*_ values in the range 1.67 to 1.73 mm s^−1^, whilst their ligands have p.q.s. values of − 0.35 to − 0.34 mm s^−1^. The data for these two complexes are plotted in Fig. 4 and fall on the respective trend lines for the two porphyrin complexes at the top of the figure on the right. As will become apparent further on in this work these are the smallest absolute p.q.s. values for N bonding ligands and for the Fe(II) atom to bind with them in the TMP complexes requires the macrocycle to play a significant role. We will return to this later when we consider the bond lengths of these complexes.

**Table 8:** ^57^Fe Mossbauer data for high-spin [Fe(PPIX)L’’] complexes, low-spin [Fe(PPIX)(CO)L’’] complexes where L’’ = a sulphur bonding ligand and reference data

Compound	T °K	*δ*_obs_ mm s^−1^	*δ*_calc_ mm s^−1^	p.c.s. mm s^−1^	*Δ*E_Qobs_ mm s^−1^	*Δ*E_Qcalc_ mm s^−1^	p.q.s mm s^−1^	Ref
[Fe(PPIX)L’’]^a^								
L’’								
ethylmercaptoacetate	80	0.94			2.41			[27]
2-Mercaptoethanol	80	0.95			2.39			[27]
Thiophenol	80	0.97			2.43			[27]
Thiocresol	80	0.99			2.45			[27]
Ethanthiol	80	0.97			2.31			[27]
[Fe(PPIX)(CO)L’’]^b^								
Ethylmercaptoacetate	80	0.31	0.31	0.03	0.60	0.60	− 0.40	[27]
2-Mercaptoethanol	80	0.30	0.31	0.03	0.80	0.80	− 0.30	[27, 66]
Thiophenol	80	0.35	0.38	0.10	0.94	0.94	− 0.23	[27, 66]
Thiocresol	80	0.34	0.34	0.06	0.73	0.73	− 0.335	[27]
Ethandiol	80	0.37	0.37	0.09	0.85	0.85	− 0.275	[27]
[Fe(TPPS)L’’]^a^				`				
thiophenol	78	0.94			2.36			[66]
2-mercaptoethanol	78	0.54			1.07			[66]
[Fe(TPPS)(CO)L]^b^								
L = thiophenol	78	0.42	0.39	0.10	0.48	0.48	− 0.465	[66]
2-Mercaptoethanol	78	0.36	0.32	0.03	0.46	0.46	− 0.475	[66]
Reference data								
[Fe(TPpivP)(SC_6_HF_4_)][KC222]^a^	85	0.91			2.37			[124]
[Fe(TPpivP)(SC_6_HF_4_)][NaC222]^a^	77	0.89			2.36			[124]
[Fe(TPpivP)(SC_6_HF_4_)(CO)][NaC_12_H_24_O_6_]^b^	4.2	0.39	0.39	0.10	0.56	0.56	− 0.43	[124]
	4.2	0.92			2.45			[124
P-450_cam_ + camphor, reduced^a^	173	0.86			2.35			[117]
P-450_cam_ + camphor, reduced + CO	4.2	0.38			0.32			[116]
	200	0.34			0.34			[116]
Cytochrome c (reduced form)	100	0.60			1.34			[126]
Cytochrome c (reduced form)		0.58			1.20			[127]

^a^High-spin [Fe(II)(Por)L’’] complexes with typical Mössbauer parameters

^b^Low-spin six-coordinate [Fe(II)(Por)(CO)L] complexes

As discussed in the paragraph above two S-bonding ligands will not form a six-co-ordinated complex with [Fe(II)(PPIX)]. However, in the c-type cytochromes six-coordinate-low-spin Fe(II) haem complexes form the prosthetic groups. In these cytochromes the vinyl groups of PPIX have been involved in forming thioether bonds with cystine residues donated by the protein, thereby covalently linking the haem to the protein [5]. The Fe(II) atom in the resulting modified [Fe(PPIX)] is said to have a different affinity for binding ligands and the binding constant of the ferrous form for sulphur ligands is enhanced [125]. Hence c-type Fe(II) haems bind their methionine ligands very tightly [125]. The surprisingly high p.q.s. values for some of the S-ligands we found from four of the complexes above (where we commented that other other effects must be tightening the bonding of the S-ligands may be relevant to the bonding in c-type-Fe(II) haems. All c-type cytochromes have histidine (N) and methionine (S) as the axial ligands bonding to the Fe(II) in the haem [5]. The Mössbauer parameters of several c-type cytochromes have been reported, for example:- Cytochrome c from Methilococcus capsulatus was enriched with ^57^Fe in vivo [126] and was studied to understand the dynamics of the protein; whilst the first reported study was about reduced and oxidised horse cytochrome c [127]. The Mössbauer parameters reported for the reduced cytochromes c given in these studies are reported in Table 8. If the p.q.s. values for histidine and ethyl 2-mercaptoacetate, (EMCA), derived in this work are used with that for [Fe(II)(PPIX)] to calculate a *ΔE*_*Q*_ value for [Fe(II)(PPIX)(His)(EMCA)] the result is 1.24 mm s^−1^. This *ΔE*_*Q*_ value is close to those reported for the cytochromes c and shows the benefit/validity of the approach manifested in Figs. 2, 3 and 4. It may also indicate that the covalent links to the protein from the haem do not have much effect on the bonding at the Fe(II) atom and are more important to its position during the oxidation/reduction cycle.

It should be noted that if the p.q.s. value for thiophenol (which is not similar to methionine) had been used with that of histidine, the resulting *ΔE*_*Q*calc_ value would have been 1.58 mm s^−1^ which as discussed above is towards the extreme end of stable low-spin Fe(II) complexes. Obviously the p.q.s. value for histidine is amongst the largest of the nitrogenous ligands and other such ligands with smaller p.q.s. values would not be able to form six-co-ordinated complexes with [Fe(II)(PPIX)] and a S-bonding ligand. This shows the important choice that natural processes have arrived at with evolving histidine as the fifth ligand for [Fe(II)(PPIX)]. Clearly histidine residues have the advantages of size, shape, available electron density and orbitals for bonding, which together must satisfy the requirements of the haem proteins. Presumably, the kinetic and thermodynamic properties of the histidine residues are the main principles/drives in the evolution of these important molecules for oxygen transport and storage.

Table 9 presents the Mössbauer parameters and some cone angle data for [Fe(II)(Por)L_2_] complexes for phosphine and phosphite ligands taken from the literature [98, 128–131] and some of our unpublished results on [Fe(II)(PPIX)L_2_] frozen solutions at 78 °K. The p.q.s. values derived from these complexes are comparable to previously derived values for non-haem complexes [73–75, 98, 128, 129]. The first point of interest is that the *δ* values are in the range between those of the [Fe(II)(Por)L_2_] and those of the [Fe(II)(Por)(CO)L] complexes (where L = a nitrogenous ligand). We would expect the p.c.s. value for the porphyrins to be likewise between those two sets of complexes and this is found in the calculations, the p.c.s. values for the porphyrins found and used to calculate the p.c.s. values for the P ligands are given in a footnote to Table 9. The p.c.s. values for the P-ligands are also presented in Table 9. They compare well with those given by Bancroft and Libbey [75]. The only poor fit between observed *δ*_obs_ and *δ*_calc_ was for the compound [Fe(II)(OEP)(PMe_3_)(2-MeImH)], we note the paper presents this datum without a comment and we cannot at this time explain the discrepancy. The p.q.s. value for P(OMe)_3_ in our previous work [76] and that of Bancroft et al. [74, 75] was − 0.63 mm s^−1^, whereas in these compounds a better value is − 0.65 mm s^−1^. This value and others derived from the [Fe(II)(TPP)L_2_] complexes in Table 9 were then used to fit all the complexes in the Table. The p.q.s. values for these ligands are also interesting, in that the values need to differ for the P ligands when they bond to [Fe(II)(Pc)] in comparison to when they bond to [Fe(II)(Por)] in that for the former they lead to higher calculated values than observed. We interpret this to mean that the Pc macrocycle cannot amend its π-bonding to the Fe(II) very well as also observed for the bis-carbonyl complex discussed earlier herein.

**Table 9:** ^57^Fe Mossbauer data low-spin [Fe(por)L_2_] complexes (where L = phosphorus ligands) and reference data

Compound	T °K	*δ*_obs_ mm s^−1 a^	*δ*_calc_ mm s^−1^	p.c.s. mm s^−1^ (value of P-axial ligand)	*Δ*E_Qobs_ mm s^−1^	*Δ*E_Qcalc_ mm s^−1^	p.q.s mm s^−1^ (value of P-axial ligand)	Cone Angle ( ˚)^b^	Ref
[Fe(PMXPP)(TMPPE)_2_]	298	0.32	0.33	0.025	0.47*	0.47	*− *0.6625		[128]
[Fe(PMXPP)((EtO)_3_P)_2_]	298	0.37	0.34	0.03	0.69	0.51	*− *0.6525	109	[128]
[Fe(PMXPP)((BuO)_2_PhP)_2_]	298	0.34	0.35	0.035	0.59	0.64	*− *0.58	116^c^	[128]
[Fe(PMXPP)((Bu)_3_P)_2_]	298	0.38	0.36	0.04	0.82	0.90	*− *0.555	132	[128]
[Fe(PMXPP)((BuO)Ph_2_P)_2_]	298	0.38	0.38	0.05	0.83	1.06	*− *0.515	132^c^	[128]
[Fe(PMXPP)((MeO)Ph_2_P)_2_]	298	0.40	0.40	0.06	1.06*	1.06	*− *0.515	132	[128]
[Fe(PClPP)(TMPPE)_2_]	298	0.35	0.34	0.025	0.46	0.45	*− *0.6625		[128]
[Fe(TPP)((MeO)_3_P)_2_]	298	0.35	0.35	0.03	0.46*	0.46	*− *0.655	107	[129]
[Fe(TPP)((EtO)_3_P)_2_]	298	0.34	0.35	0.03	0.47*	0.47	*− *0.6525	109	[129]
[Fe(TPP)((BuO)_3_P)_2_]	298	0.35	0.36	0.035	0.48*	0.48	*− *0.65	112	[129]
[Fe(TPP)((EtO)_2_PhP)_2_]	78	0.33^d^	0.33	0.02	0.58*	0.58	*− *0.625	116	TW
[Fe(TPP)((Et)_3_P)_2_]	298	0.39	0.39	0.05	0.86*	0.86	*− *0.555	132	[129]
[Fe(TPP)((Bu)_3_P)_2_]	298	0.37	0.37	0.04	0.86*	0.86	*− *0.555	132	[129]
[Fe(TPP)((Bu)_3_P)_2_]	78	0.41^d^			0.79			132	TW
[Fe(PPIX)((MeO)_3_P)_2_]	78	0.37^d^	0.35	0.03	0.44	0.42	*− *0.655	107	TW
[Fe(PPIX)((BuO)_3_P)_2_]	78	0.39^d^	0.36	0.035	0.64	0.46	*− *0.6475	112^c^	TW
[Fe(PPIX)((EtO)_2_PhP)_2_]	78	0.37^d^	0.33	0.02	0.54	0.54	*− *0.625	116	TW
[Fe(PPIX)((Bu)_3_P)_2_]	78	0.43^d^	0.37	0.04	0.81	0.82	*− *0.555	132	TW
[Fe(OEP)(PMe_3_)_2_]	77	0.38^d^	0.39	0.06	+ 0.38^e^	0.47	*− *0.6555	116	[98, 131]
[Fe(OEP)(PMe_3_)(2-MeImH)]	77	0.46^d^	0.365		+ 1.05	1.015			[131]
[Fe(TMP)(PMe_3_)_2_]	77	0.38^d^	0.39	0.06	+ 0.47	0.47	*− *0.655	116	[98]
[Fe(TMP)(PMe_3_)(4-NMe_2_Py)]	77	0.40^d^	0.405		+ 0.85	0.82			[98]
[Fe(TMP)(PMe_3_)(4-CNPy)]	77	0.41^d^	0.405		+ 0.88	0.84			[98]
[Fe(TMP)(PMe_3_)(N-MeIm)]	77	0.40^d^	0.41		+ 0.75	0.78			[98]
[Fe(Pc)((EtO)_3_P)_2_]	291	0.22	0.22	0.03	1.07	1.25	*− *0.6525	109	[129]
[Fe(Pc)((BuO)_3_P)_2_]	78	0.20^d^	0.23	0.035	1.03	1.26	*− *0.65	112^c^	TW
[Fe(Pc)((EtO)_2_PhP)_2_]	78	0.21^d^	0.20	0.02	1.04	1.36	*− *0.625	116	TW
[Fe(Pc)((Et)_3_P)_2_]	298	0.25	0.26	0.05	1.54	1.64	*− *0.555	132	[129]
[Fe(Pc)((Bu)_3_P)_2_]	291	0.24	0.24	0.04	1.57	1.64	*− *0.555	132	[129]
[Fe(Pc)((Bu)_3_P)_2_]	78	0.27^d^			1.55			132	TW

^a^The chemical shifts were used to derive p.c.s. values for both the macrocyclic ligands and the P ligands. The p.c.s. values for the macrocyclic ligands derived and used here are:- PMXPP = 0.28 mm s^−1^, PClPP = 0.28 mm s^−1^, TPP = 0.29 mm s^−1^, PPIX = 0.29 mm s^−1^, OEP = 0.27 mm s^−1^, TMP = 0.27 mm s^−1^, Pc = 0.16 mm s^−1^

^b^Cone angle data taken from ref. [130]. ^*^Used to calculate the p.q.s. values of the P-ligands

^c^Estimated from similar ligands in Ref. [130]

^d^Calculated from the 78 °K value to 298 °K and adjusted to be relative to stainless steel. Tw = this work

^e^Value taken from 4.2 °K data ref 131 before fluxional/rotation onset. We note in ref 131 the calculated value was 0.43 mm s^−1^

It is important to consider the cone angles [130] of the P-ligands which are relevant to facilitate further understanding of the factors that influence/control their bonding We previously reported an inverse relationship between the cone angle of the P ligand and the size of its p.q.s. value so that the larger cone angles were associated with the smaller p.q.s. values [76]. This finding holds true in Table 9 both in the [Fe(II)(Pc)L_2_] and [Fe(II)(Por)L_2_] complexes. Finally, the complexes containing P(Me_3_) [98, 131] are worth discussing, the p.q.s. value we derived herein for this ligand is − 0.655 mm s^−1^ which is very close to the value of − 0.66 mm s^−1^ found in non-haem complexes by Bancroft et al. [74, 75]. In the cases where the complexes containing P(Me_3_) [98, 131] are bound opposite to nitrogenous ligands the calculated *ΔE*_*Q*_ values are very close to the observed experimental ones; the only exception is for the complex [Fe(II)(OEP)(PMe_3_)_2_]. In the latter complex evidence for motion or at least fluxional movement in the two axial P(Me_3_) [98, 131] ligands was reported, leading to smaller than expected *ΔE*_*Q*_ values. This may perhaps explain why our point charge calculations, which do not consider such effects, are not good for this complex, whereas they work for the [Fe(II)(TMP)(PMe_3_)_2_ and other complexes containing this ligand where fluxional motion does not occur (see Table 9). When the phenomenon of motion occurs, it causes the collapse of *ΔE*_*Q*_ values as a function of temperature, usually this is seen by a noticeable decrease as a function of increasing temperature. To be observed it must occur within the time frame of the Mossbauer event. Only some molecules do it and not that many in Fe(por) complexes, though oxygen is thought to do this when it is bound to the Fe atoms in Hb. The other point of interest for the P(Me_3_) ligand relative to this work is that the cone angle reported by Tolman [130] of 116˚ would have been expected to be associated with a smaller p.q.s. value than − 0.655 mm s^−1^ (which is larger than would have been expected form the relationship between cone angles and p.q.s. values reported herein). A possible explanation for this is that the P-bonding ligands unlike the aliphatic nitrogenous ligands are all able to contribute both *σ*- and *π*-bonding to the Fe(II). This is of course because the P ligands have both 3p and 3d orbitals available for bonding and this may strengthen the bonding of this less sterically hindered more electron donating phosphine ligand [128].

In Fig. 3 we presented a plot of *ΔE*_*Q*_ versus calculated p.q.s. for the [Fe(II)(Por)L1L2] complexes (where L1 and L2 represented a range of axial ligands). Trend lines for the various ligands are indicated by numbers. 1: L1 = L2 = pip; 2: L1 = L2 = py; 3: L1 = L2 = 1-Me1m; 4: L1 = L2 = P(Bu)_3_; 5: L1 = pip, L2 = CO; 6: L1 = L2 = CO. Trend line 4 for the P(Bu)_3_ ligands lies below the nitrogenous ligands but above Trend line 5 as expected for the phosphorus ligands which we have shown in Table 9 to have p.q.s. values indicating stronger bonding than the nitrogenous ligands but not as strong as the carbonyl ligands.

Much of the discussion above on the low-spin [Fe(II)(Por)(CO)L] complexes is targeted at understanding how CO binds to the Fe(II) atom [1]. This as stated is important as it is a notorious inhibitor of respiration by forming stable low-spin haemoprotein carbonyls. Cyanide (CN^−^) also inhibits respiration but its modus operandi is to inhibit O_2_ reduction [131]. When CN^−^ binds to low-spin [Fe(II)(Por)] positively charged ions are required for charge balance, though unlike O_2_ and CO, it can also readily bind to [Fe(III)(Por)].

There have been relatively few studies on CN^−^ binding to [Fe(II)(Por)] probably due to such complexes being unstable even at alkali pH [132]. Scheidt et al. have reported the structures of several low-spin [Fe(II)(TPP)] cyanide complexes [122, 133, 134] including three six-co-ordinate complexes; two forms of [K(222)]_2_[Fe(II)(TPP)(CN)_2_] and [K(222)][Fe(II)(TPP)(CN)(1-MeIm)] see Table 10 for their Mössbauer parameters. As Scheidt et al. [122] discuss the two bis(cyano) complexes have *ΔE*_*Q*_ values that show a temperature dependence change that is opposite to that seen for [Fe(II)(OEP)(CO)_2_] [122]. They state that such temperature dependence is usually considered to be the result of low-lying excited states and that this must be true for both classes of complexes. They suggest that the differing direction of change is most likely to be due to the complexes having *ΔE*_*Q*_ values that are opposite [122]. They state that it is difficult to obtain the sign of *ΔE*_*Q*_ when the magnitude is small [122, 123] and that the signs are not currently determined.

**Table 10:** ^57^Fe Mossbauer data low-spin [(Fe(TPP)(CN)_2_]^2−^ and related complexes

Compound	T °K	*δ*_obs_ mm s^−1^	*δ*_calc_ mm s^−1^	*ΔE*_Qobs_ mm s^−1^	*ΔE*_Qcalc_ mm s^−1^	Ref
[K(222)][Fe(TPP)(CN)(1-MeIm)]	298 100 16	0.35^a^ 0.46 0.48	0.34	0.62^b^ 0.61 0.60	0.62	[122]
[K(222)]_2_[Fe(TPP)(CN)_2_]	296 100 20	0.31 0.40 0.40	0.31	0.13 0.24 0.27	0.16	[122]
[K(222)]_2_ [Fe(TPP)(CN)_2_]	298 100 15	0.31 0.38 0.38	0.31	0.07 0.13 0.15	0.16	[122]
[Fe(PPIX)(CN)_2_]^2−^	77	0.34	0.33	0.0	0.0	TW
[Fe(TPPS)(CN)_2_]^2−^	77	0.33	0.34	0.0	0.01	TW
[Fe(TNPS)(CN)_2_]^2−^	77	0.33		0.0		TW
[Fe(P)_2_(MeP)_2_PS)(CN)_2_]^2−^	77	0.36		0.0		TW

^a^Used to calculate p.c.s. value for CN^−^

^b^Used to calculate p.q.s. value for CN^−^

In this paper we have presented a case for the sign of the *ΔE*_*Q*_ value being negative for [Fe(II)(OEP)(CO)_2_] and we now present the results of our calculations on the three cyanide complexes. The first step was to derive a value for the p.q.s. value for CN^−^. A value is given in the literature [60, 63] of − 0.84 mm s^−1^, however this value was not found in a porphyrin complex and if used to fit [K(222)][Fe(II)(TPP)(CN)(1-MeIm)], it generates a p.q.s. value for 1-MeIm of − 0.38 mm s^−1^ compared to the value of − 0.50 mm s^−1^ found and used in this work for several different complexes. Using − 0.50 mm s^−1^ for 1-MeIm in [K(222)]^+^ [Fe(II)(TPP)(CN)(1-MeIm)]^−^ gives a p.q.s. value for CN^−^ of − 0.73 mm s^−1^ and this gives a calculated *ΔE*_*Q*_ value of 0.16 mm s^−1^ which is close to the observed values for the two complexes (see Table 10). Moreover, it has the opposite sign to that of the [Fe(II)(OEP)Fe(CO)_2_] complex. If this value for the p.q.s. is plotted in Fig. 4 against a *ΔE*_*Q*_ value of 0.18 mm s^−1^, then it fits well on the trend lines, which again in our opinion is good evidence for the assignment. The *δ*_obs_ values for the CN complexes in Table 10 are all low and close to those of the CO complexes discussed earlier. So, the p.c.s. value for TPP in Table 7 was used in deriving the p.c.s. value for CN^−^. The differences found for the p.c.s. values of CN^−^ (− 0.02 mm s^−1^) and CO (0.0 mm s^−1^) are in accord with lowered π-acceptance by the former ligand as discussed by others [122]. The differences found for the p.q.s. values of CN^−^ and CO are also consistent with greater σ-donation to Fe by the former ligand again in agreement with previous discussion [122]. Table 10 also includes two of our previously unpublished results for [Fe(II)(PPIX)(CN)_2_] and [Fe(II)(TPPS)(CN)_2_] complexes in frozen solution. Both these complexes manifest no observable *Δ*E_Q_ values. This suggests that there is a symmetrical electric field around the Fe(II) atoms in these two complexes. The calculated p.q.s. values based on this for the CN^−^ ligands are − 0.75 and − 0.76, respectively for the [Fe(II)(TPPS)(CN)_2_] and[Fe(II)(PPIX)(CN)_2_] complexes. These p.q.s. values are lower than those of the CO ligands (range − 0.78 to 0.88 mm s^−1^) (see Tables 5 and 7) in the corresponding bis(CO) porphyrin complexes again supporting the change in sign of V_zz_ in the latter complexes.

The *δ*_obs_ values for the CN complexes in Table 10 are all low and close to those of the CO complexes_._

### Implications for oxygen binding to [Fe(II)(Por)] in biological molecules

It should be stated that all, or nearly all (except for some of the picket fence porphyrins) of the model compounds considered in the point charge calculations above have had axial ligands that were free to bond to the Fe(II) in the haems impaired only by their own steric bonding limitations. In natural systems the geometry of binding environment around the haem site in the protein can also affect how the second axial ligand binds An example of this is in Mb; in the hydrophobic pocket surrounding the haem and close to the oxygen binding site a second histidine residue (the distal histidine) forms a hydrogen bond to the bound oxygen that constrains the Fe–O–O angle to 121˚ and is said to ensure that the Fe(II) atom is not oxidised to the ferric form and causes the protein to lose its function [5]. This hydrogen bonding can also aid the binding of the oxygen molecule (and is also present in Hb); this is important as there has been and still is an argument over whether in Hb the iron is present as low-spin Fe(II) bound to an O_2_ molecule, or as low-spin Fe(III) bound to an O_2_^−^ (the superoxide ion) to give a net zero spin (this argument has continued for nearly 60 years) [135, 136].

Another property of the oxygen binding haem proteins is the fact that the axial histidine is bound to the backbone of the protein so that the protein moves when the PPIX(FeII) moieties bind to and unbind oxygen molecules. When the oxygen is not bound, the Fe(II) atom moves out of the porphyrin plane towards the histidine (going into a five-coordinate geometry): it is then in a high-spin state. As the histidine is linked to the backbone of the protein this causes the entire backbone to move. The most obvious conclusion to be drawn is that these axial histidine residues have restrictions on their bonding properties compared to a free unrestricted histidine molecule. Presumably, their bonding is at least partially controlled/varied by the movements in the backbone of the protein**.** This would also account for the larger *ΔE*_*Q*_ values for Hb and Mb and in some picket fence haems where such restrictions have been “modelled in”. We found it extremely difficult to bind oxygen to “naked” [Fe(II)(PPIX)] even in the presence of reducing agents and axial ligands. However, we were able to achieve *Δ*E_Q_ values for [Fe(II)(PPIX)] bound to oxygen in the range 2.20–2.02 mm s^−1^ in the presence of 2-methylpiperidine and mercaptoethanol [28] at liquid nitrogen temperature in frozen solution. We suggested that the periphery vinyl groups on the PPIX may have provided some steric hindrance to the oxygen molecule preventing the oxidation of the Fe(II). However, in the absence of the axial ligands some evidence for the *μ*-oxo-bridged PPIX iron(III) oligomer was always found [24, 28].

The *ΔE*_*Q*_ values for Hb, Mb and some picket fence haems bound to oxygen are in the range 2.32–2.10 mm s^−1^ [102]. These values as well as those for the chemical shifts are very like those of low-spin Fe(III) porphyrin complexes. The *ΔE*_*Q*_ values that we have found for the [Fe(II)(Por)L_2_] and [Fe(II)(Por)L_1_L_2_] complexes in this work range from around − 0.5 to 1.76 mm s^−1^. If the iron in Hb and the model compounds was in fact present as low-spin Fe(II) then using the p.q.s. value of − 3.04 mm s^−1^ for PPIX and a p.q.s. value of − 0.50 mm s^−1^ for histidine, the p.q.s. value for the oxygen molecule will be close to zero or in fact slightly positive (in the range 0.03–0.14 mm s^−1^) suggesting little or no bonding. This would mean that it is either primarily held by the hydrogen bond (to the histidine residue) and its electronic interaction with the low-spin Fe(II) atom is minimal or alternatively a second possibility must be considered which we now discuss. As it is known that the oxygen molecule binds side on to the porphyrin plane, albeit at an angle rather than being parallel, it may well have a bonding interaction via its double bond with the porphyrin π-electron cloud. A much more likely explanation is that our findings that the point charge model indicates such weak bonding from oxygen to low-spin Fe(II) is in fact definitive evidence for the iron being low-spin Fe(III) bound to a superoxide ion [135, 136].

### Comparison to other macrocycles p.q.s. values

Only TAAB [77] of the other known macrocycle nitrogen ligands has a symmetrical centre. This ligand lies well away from the porphyrins in Fig. 1. The π-bonding is similar to that of the monodentate π-ligands and its σ-bonding is only slightly better. This finding indicates that it is the conjugation of the double bonds of the atoms in the porphyrin plane that control and focus the four porphyrin N atoms to bond to the iron imposing a D_4h_ symmetry that appears vital for its role in natural systems. We will return to this point later in the paper.

### Bond lengths from known octahedral low-spin (porphyrinato)iron(II) structures

Of the porphyrins studied in this work low-spin octahedral (porphyrinato)iron(II) crystal structures are known for OEP, TPP, TMP, TpivPP and PMXPP. The Fe–N distance (N of porphyrin ring) is in the range 1.961–2.001 Å for the known structures [19–21, 56, 93, 100, 101, 103, 107, 111, 112, 120, 137–141]. Moreover, these Fe–N distances show little change from porphyrin to porphyrin for any spin state or valence state though the bond lengths change with spin state and valence state [18, 19]. The Fe–N distance in low-spin pc compounds are significantly smaller 1.89(2) Å in [Fe(pc)(CO)DMF] [103] and 1.937(3) Å in [Fe(pc)(4-Mepy)_2_] [142] than the equivalent distances in the iron porphyrins, and as demonstrated vide infra the p.q.s. values of pc show it is a stronger σ-donor. It should be noticed that the smallest Fe–N distance (N of porphyrin ring) given above is 1.961 Å, this is for the complex [Fe(TMP)(2-MeHIm)_2_] [21] which will be discussed at the end of this section, and without this complex, for the other complexes in Table 11 the Fe–N distances (N of porphyrin ring) range from 1.988 to 2.001 Å.

**Table 11:** Crystal Structure data, ^57^Fe Mossbauer data and p.q.s. values for low-spin [Fe(Por)L(CO)] complexes and [Fe(Por)L_2_] complexes

Compound	N_ax_-Fe Å	N_Por_-Fe	*ΔE*_Qobs_ mm s^−1^	p.q.s mm ^s−1^	Refs
[Fe(TPP)(pip)_2_]	2.127(3)	2.004(6)	1.52	− 0.405	[91, 120]
[Fe(TPP)(py)_2_]	2.037(1)	2.001(2)	1.22	*− *0.46	[90, 108, 137, 138]
[Fe(TPP)(1-VinylIm)_2_]	2.004(2)	2.001(2)	1.02	*− *0.50	[93]
[Fe(TPP)(1-BzylIm)_2_]	2.017(4)	1.993(9)	1.02	*− *0.50	[93]
[Fe(TPP)(1-MeIm)_2_]	2.014(5)	1.997(6)	1.07	*− *0.50	[93]
[Fe(TPP)(py)(CO)]	2.10(1)	2.02(3)	0.57	*− *0.46	[108, 139]
[Fe(TPP)(1-MeIm)(CO)]	2.071(2)	2.003(5)	0.35	*− *0.50	[140]
[Fe(TFPP)(Fe(C_5_H_5_)(C_4_H_4_N))_2_]	2.05(2)	2,01(2)	1.25		[70]
[Fe(TpivPP)(1-MeIm)_2_]	1.9958(19)	1.992(3)	1.05	*− *0.50	[101]
[Fe(TpivPP)(1-EtIm)_2_]	2.0244(18)	1.993(6)	1.07	*− *0.50	[101]
[Fe(TpivPP)(1-VinylIm)_2_]	1.9979(19)	1.998(5)	1.07	*− *0.50	[101]
[Fe(Tpiv_2_C_12_P)(1-MeIm)(CO)]^a^	2.062(5)	1.999(3)	0.27	*− *0.50	[111, 112, 143]
[Fe(OEP)(1-MeIm)(CO)]	2.077(3)	2.000(3)	0.40	*− *0.50	[109, 144]
[Fe(TMP)(4-CNpy)_2_]	1996(2)	1.993(2)	1.13	*− *0.47	[100]
[Fe(TMP)(4-Mepy)_2_]	2.010(2)	1.988(2)	1.12	*− *0.47	[100]
[Fe(TMP)(2-MeHIm)_2_]	2.047(3) 2.030(3)	1.964(5)	1.78	*− *0.3475	[20]
[Fe(TMP)(2-MeHIm)_2_]	2.032(3) 2.028(3)	1.961(7)	1.78	*− *0.3475	[20]
[Fe(PMXPP)(amp)_2_]	2.037(2)	1.988(2)	1.04	*− *0.52	[107]

^a^Crystal structural data is for this complex, but Mossbauer data is for the [Fe(Tpiv_P_P)(1-MeIm)(CO)] complex

**Fig. 6:**
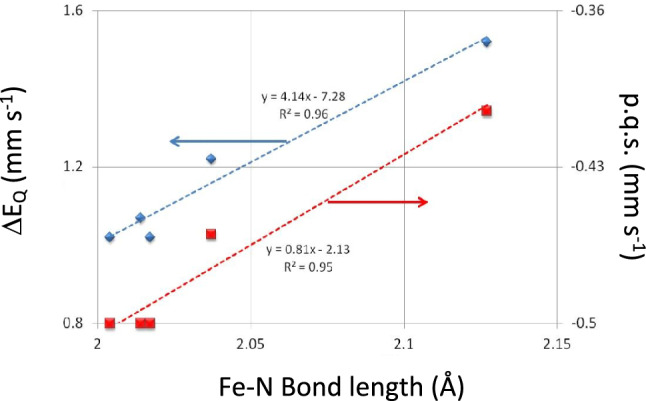
Plot 1. (Blue diamonds) of Axial nitrogenous base to Fe(II) distances against the *ΔE*_Q_ values of the [Fe(II)(TPP)L_2_] complexes. Plot 2. (Red squares)of Axial nitrogenous base to Fe(II) distances against the p.q.s. values of the [Fe(II)(TPP)L_2_] complexes. (Data for both plots presented in Table 11)

Typical Fe–N (where N is an axial nitrogen ligand,) bond lengths depend on the nature of the nitrogen ligand (See bond lengths in Table 11). For aromatic ligands such as py Fe–N = 2.10(1) Å in [Fe(TPP)(py)(CO)] [138], 2.037(1) Å and 2.039(1) Å in [Fe(TPP)(py)_2_] [137, 138]. In a substituted TPP the (5,15-[2,2′-(dodecanediamido) diphenyl]: a,cx-l0,20-bis(o-pivaloylaminophenyl)porphyrin = Tpiv_2_C_12_P) complex [Fe(Tpiv_2_C_12_P)(1-MeIm)(CO)] [143] the Fe–N _Im_ distance is 2.062(5) Å is long as it is in [Fe(II)(OEP)(1-MeIm)(CO)] [144] (Fe–N _Im_ distance is 2.077(3) Å), whereas for 1-MeIm in [Fe(TPP)(1-MeIm)_2_] [93] Fe–N _Im_ = 2.014(5) Å. Shorter axial bonds are also apparent in [Fe(TPP)(1-VinylIm)_2_] [93] Fe–N _Im_ = 2.004(2) Å and in [Fe(TPP)(1-BzLIm)_2_] [93] Fe–N = 2.017(4). Even shorter Fe–N _Im_ axial bonds are found in [Fe(TpivPp)(1-MeIm)_2_] [101] Fe–N _Im_ = 1.9958(19) Å and 1.9921(18) Å and in [Fe(TpivPp)(1-VinylIm)_2_] [101] the Fe–N _Im_ distances are 1.9979(19) Å and 1.9866(19) Å. There is only one example of a saturated axial ligand piperidine. For piperidine the analogous distance in [Fe(TPP)(pip)_2_] [120] is 2.127(3) Å. The bond lengths to iron in [Fe(TPP)(L_2_)] (L = nitrogen ligand) order as follows: 1R-Im < pyridine < piperidine, whereas for the p.q.s. values the inverse order is found. This is as expected as imidazole is the best σ-donor of the first two whilst piperidine which is only able to σ-donate is sterically hindered. It is therefore apparent to this point that the known crystal structures are in line with the bonding implications of the p.q.s. values derived in this work. This can be appreciated by considering the data presented in Table 11, though there are not many low-spin [(Por)Fe(II)L_2_] where L = the same porphyrin, in fact there are five [Fe(II)(TPP)L_2_)] complexes and plotting their axial bond lengths against their *ΔE*_*Q*_ values (see Fig. 6 blue diamonds) gives a trend line that gives a good linear correlation between these parameters. Thus, the asymmetry of the electric field around the Fe(II) atom is directly related to the axial bond length of the metal–ligand bond. Clearly their axial bond lengths can also be plotted against the p.q.s. values of the axial ligand (see Fig. 6 red squares). Again, there is a good correlation, here it is apparent that the relationship is now to the ligand contribution to the asymmetry of the electric field. From the trend line in Fig. 6 (red squares) it is apparent that as the p.q.s. value becomes larger (the axial ligand bonding is stronger), the axial bond length decreases. The correlation is not quite as good as the p.q.s. values arise from a compromise of many data for different complexes. Unfortunately, it is not possible to do this exercise for other individual porphyrins in Table 11 as there is not enough data in the literature.

**Fig. 7:**
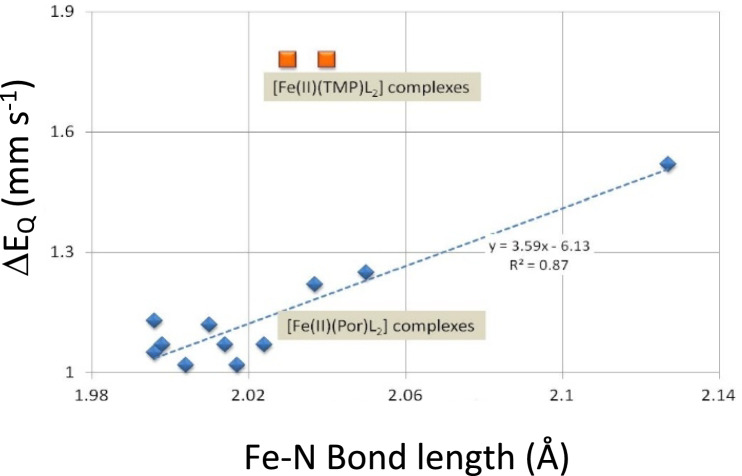
Plot of Axial nitrogenous base to Fe(II) distances against the *ΔE*_Q_ values of 11 [Fe(II)(Por)L_2_] complexes on the trend line and two [Fe(II)(TMP)L_2_] complexes (in orange) far off the line (data presented in Table 11)

However, the data we have listed in Table 11 are also plotted in Fig. 7 as the axial ligand bond length against the *ΔE*_*Q*_ values; there is a good correlation for eleven of the data in the Table even though different porphyrins are involved.

The [Fe(II)(TFPP)(Fe(C_5_H_5_)(C_4_H_4_N))_2_] [70] complex is the only compound of this porphyrin type studied by Mössbauer spectroscopy to date, so we cannot yet derive p.q.s data for it; hence the plot uses its *ΔE*_*Q*_ values. The plot indicates that as the nitrogenous ligand becomes just σ-bonding the bond length gets longer, so pip is the longest and the next longest is for [Fe(II)(TFPP)(Fe(C_5_H_5_)(C_4_H_4_N))_2_], we previously discussed that the axial ligands in this complex were predominantly good σ-donors with little or no π-bonding to or from the porphyrin Fe(II); evidence for this was derived from the (Fe(C_5_H_5_)(C_4_H_4_N)) Mossbauer parameters [70]. All the other complexes in Fig. 7 are both σ- and π-bonding to their porphyrin Fe(II) centres. It is worth noting that only two of the known [Fe(II)(TMP)L_2_] complexes given in Table 11 fit the trend line, whereas those with the two with large *ΔE*_*Q*_ values do not. This is evidence that TMP complexes are very different; although we could fit p.q.s. values, the fact that they do not fit on the trend line in Fig. 7 suggests that they are different to the other porphyrins. We believe this is due to their extended structures affecting/restricting the way that axial ligands can bond.

The complex [Fe(II)(PMXPP)(amp)_2_] [107] is the only complex in Table 11 that has a Fe–N _Axial_ bonded ligand where the N is an aminoethyl group that then bonds to morph. Thus, this ligand is saturated at the N bond and would be expected to be σ-bonding to the Fe(II) atom in keeping with this the bond length is 2.037(2) Å, unfortunately the Mössbauer data for this complex are fitted without errors and the data looks poor (see footnote under Table 3). Although we have derived a p.q.s. value (which is close to the other aliphatic amines) for the ligand we have not included it in Fig. 7 as we do not know the error on the *ΔE*_*Q*_ value.

We will now consider the [Fe(II)(TMP)L_2_] complexes that manifest larger *ΔE*_*Q*_ values in greater detail. Only the crystal structure of one such complex [Fe(II)(TMP)(2-MeHIm)_2_] [20] is known. The asymmetric unit in the unit cell of the structure was found to contain four porphyrin complexes: two of these are high-spin iron(II) five-coordinate complexes; the other two are the low-spin six-coordinate complexes (referred to as mol 1 and mol 2) [20]. The five-coordinate complexes are only indirectly relevant to this work in that we have found previously that only very weakly binding nitrogenous ligands (usually sterically hindered) form such high-spin complexes and most nitrogenous ligands exhibit cooperative binding forming six-coordinate complexes [38–41]. This is evidence that 2-MeHIm is a very weakly bonding ligand. This is in keeping with the p.q.s. value of − 0.348 mm s^−1^ that we derived for the ligand. The six-coordinate complexes in the asymmetric unit manifest two 2-MeHIm ligands with nearly perpendicular orientation. The Fe–N distances (N of porphyrin ring) average 1.964(5) Å in the first molecule (mol 1) and 1.961(7) Å in mol 2 [20]. As stated at the beginning of this section these values are much smaller than those of the other low-spin six-coordinate iron(II) porphyrins (see Table 11); there are other similar distances in other known similar low-spin [Fe(II)(Por)L_2_] complexes, but unfortunately their Mössbauer parameters have not been reported [100, 120, 140, 141]. The axial bond lengths for mols 1 and 2 of [Fe(II)(TMP)(2-MeHIm)_2_] [20] are 2.030(3) Å and 2.047(3) Å (in mol 1) and 2.032(3) and 2.028(3) Å (in mol 2). These axial distances are similar to those found for the other [Fe(II)(TMP)L_2_] complexes listed in Table 11 illustrated above, but as discussed by others, are slightly longer in keeping with the steric hinderance caused by the presence of the methyl group in the 2 position [20]. However, they are not as long as some of the axial ligands in the [Fe(II)(TPP)L_2_] complexes which is a little surprising if the p.q.s. value derived is correct. This value as discussed assumes the p.q.s. value of the TMP ligand does not vary from complex to complex. In Table 11 it is mainly the Fe–N distances (N of porphyrin ring) that are different and smaller in [Fe(II)(TMP)(2-MeHIm)_2_] [20, 100]. The other interesting feature of the [Fe(II)(TMP)(2-MeHIm)_2_] molecules is that their porphyrin planes are ruffled [21, 100], i.e., not planar. This is a very important point as the theory behind this work assumes that the [Fe(II)(Por)L_2_] complexes have D_4h_ symmetry, however, when ruffling occurs, the ring loses its D_4h_ symmetry and hence, Eq. (2) is no longer (strictly) valid. This means that the calculation of *ΔE*_*Q*_ from p.q.s. values for ruffled structures is not accurate. In the case of the two [Fe(II)(TMP)(2-MeHIm)_2_] molecules found in the asymmetric unit of the structure both have 4 Fe–N _Por_ bonds that are short but not of equal lengths thus breaking the symmetry around the Fe(II) atoms. The question then arises: is the short unequal Fe- N bonds in the ruffled porphyrin plane the driving force behind the higher observed *ΔE*_*Q*_ value of 1.78 mm s^−1^ or is there more to it? Taking these facts into consideration we can now return to the [Fe(II)(Por)(2-MeHIm)_2_]complexes in Tables 3 and 5 that have one or more 2-MeHIm ligands. As the 2-MeHIm ligand p.q.s. value is derived to be − 0.348 mm s^−1^ from [Fe(II)(TMP)(2-MeHIm)_2_] in Table 3 and a value of − 0.52 mm s ^−1^ for 2-MeIm in [Fe(II)(PPIX)(2-MeIm)_2_] can be derived from Table 4 (It should be noted that these complexes form under different pH and solution conditions where the 2-methylimidazole ligand is likely to be deprotonated in the PPIX complex), we have an inconsistency that needs to be resolved. If we assume this is because in one case the H^+^ is present and in the other the ligand is deprotonated, then we have an explanation as discussed earlier in this work. The value of − 0.348 mm s ^−1^ for p.q.s. of the protonated 2-MeHIm in for [Fe(II)(TMP)(2-MeHIm)_2_] and the other [Fe(II)(Por)L_2_] complexes manifesting large *ΔE*_*Q*_’s around 1.7 mm s^−1^ can thus be explained without referring to core ruffling arguments.

However, from this work it is apparent that these large *ΔE*_*Q*_ values are at the extreme end of the stable low-spin [Fe(II)(Por)L_2_] complexes. Thus, complexes where the axial ligands have such low p.q.s. values would not be expected to form easily. This can be understood for weak binding ligands (see section on sulphur containing axial ligands below). Of course, one possibility is that the porphyrin nitrogen atoms change their bonding properties to the Fe(II) atom because of their porphyrin cores ruffling as discussed above; and it is this that leads to the stability of the [Fe(II)(TMP)(2-MeHIm)_2_] complexes. This would lead to a change in the p.q.s. value for the TMP macrocycle itself. If we calculate the p.q.s. value for TMP based on the p.q.s. value for the deprotonated ligand, then 4 x − 0.52 + 1.67 mm s^−1^ (the observed *ΔE*_*Q*_) then a values of − 3.71 mm s^−1^ results. This p.q.s. value is different to the value found for the TMP complexes manifesting smaller *ΔE*_*Q*_s. A value of the TMP p.q.s. of − 3.71 mm s^−1^ is much closer to the p.q.s. value for pc of − 3.86 mm s^−^1, these are much larger absolute values than the other ligands shown in Figs. 1, 2, 3 and 4. This would then be evidence that the ruffled TMP has N to Fe bonding which is much more σ-donating with a much smaller π-bonding component. This might at first sight seem surprising when compared to the smaller Fe–N distances (N of porphyrin ring), but a ruffled core may be an indication of a change in the π-bonding component of the ring. It would be expected that if the porphyrin cannot donate as much π-electron density to the Fe atom, then the latter will need to compromise by donating more π-electron density to the porphyrin nitrogen atoms; this of course would be a main factor in causing the observed smaller Fe–N bond lengths. This explanation is in keeping with arguments put forward by others that have discussed these ruffled porphyrin complexes [20, 106]. It is worth mentioning that in the early 1970’s Hoard [145, 146] first suggested that there should be a quantitative relationship between the bond shortening seen in the Fe–N distances (N of porphyrin ring) and the degree of core ruffling. Evidence in support of this was reported in 2005 [20].

It seemed at first therefore to us probable that as the Fe–N_por_ distance changes and then so would the p.q.s. value for the porphyrin ligand. However, except for the TMP complexes the calculations and findings herein indicate that the p.q.s. values of the porphyrins are almost invariant irrespective of the axial ligands. However, we do have evidence for change in the p.c.s. values of the porphyrins when there is substantive change in the axial ligands (as for the CO containing complexes). The question is why is this? A possible answer is that the axial ligand bonding requirements dictate their needs to the Fe(II) and that the porphyrin changes are of secondary importance. We believe it is more likely that the porphyrin bonding to the iron compromises and tries to satisfy the demands of the iron but can only do so in a limited range of parameters, and of course all such differences are seen in the p.c.s. data. In addition, the p.q.s. data are derived from the *ΔE*_*Q*_ values which are generated by the asymmetry in the electric field experienced by the iron atom. The fact that the p.q.s values for a given porphyrin manifest little change, though clearly the Fe–N (where N = porphyrin N) distances change, suggests that if the porphyrin plane does not ruffle, then the model used herein works well. However, if the porphyrin plane manifests ruffling, then the *ΔE*_*Q*_ value is an indication of this as the ruffling causes a change in the EFG. Thus, Mössbauer spectroscopy is a good probe for porphyrin core ruffling as this is manifest as a change in the EFG arising by change in the porphyrin nitrogen bonding. Again, the simple point charge calculations reported herein are thus able to offer an insight into the bonding in all the low-spin six-coordinate iron(II) porphyrins complexes considered in this work including those in which the porphyrins bonding changes.

Low-spin [Fe(II)(OEP)(2-MeHIm)_2_] [98] also has a large *ΔE*_*Q*_ value of 1.67 mm s^−1^, unfortunately the structure of this complex has not been established, but we believe it is likely that the porphyrin plane ruffles as the OEP ethylene groups form a low picket fence or two half picket fences (one up and one down) that will interact with the axial ligands and affect the bonding. Such alignment of the ethylene groups is apparent in [Fe(II)(OEP)(1-MeIm)(CO)] [113, 144].

We have discussed situations above where the symmetry is no longer exactly D_4h_, yet it appears that the model works so long as the distortion from this symmetry is not great. It is worth further consideration to understand limitations to this tetragonal model. If ruffling of the ring occurs, we lose the fourfold axis, so, we go back to a C_2_, D_2_ or D_2h_ kind of symmetry. However, it is the electronic environment that is important and small changes distortions in the crystallographic environment may only have small effects on the electronic environment and not manifest as large changes in the EFG and therefore not upset the correlations we have presented herein. We have no evidence for where an extreme change has occurred amongst the complexes we have so far studied. In the case of different axial ligands being present without ring-ruffling we get D_4_ symmetry since we lose the ring-symmetry plane. It appears that this is acceptable if the electron density at the central Fe-atom is not changing due to this ligand difference as appears to be true for the [Fe(II)(Por)(CO)L] where the calculations work well.

**Fig. 8:**
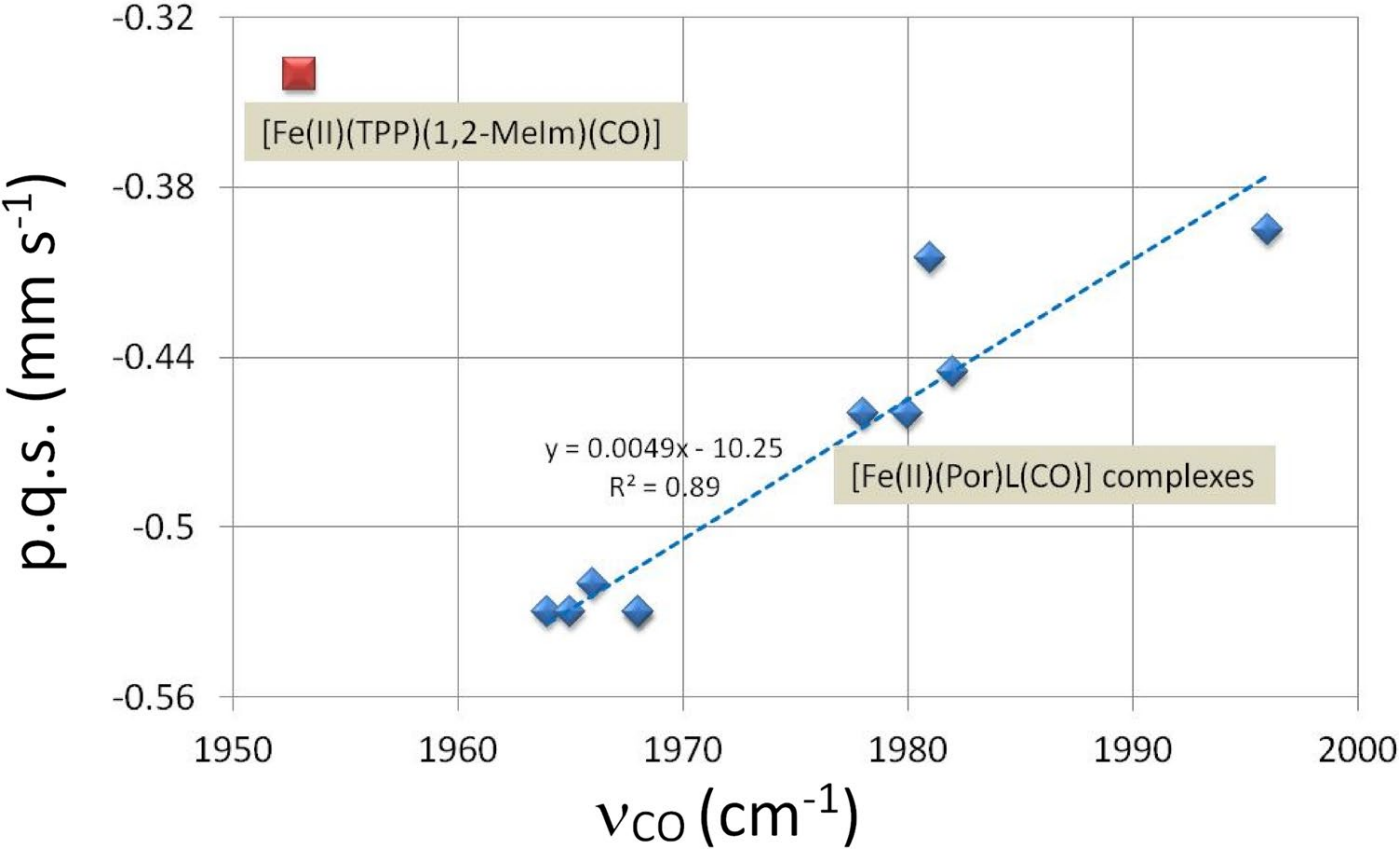
Plot of *ʋ*_CO_ cm^−1^ (carbonyl-stretching frequency) against the nitrogenous base to Fe(II) p.q.s. values of ten [Fe(II)(Por)L(CO)] complexes on the trend line and one [Fe(II)(TPP)(1,2-MeIm)(CO)] complex in red far off the line (Data presented in Tables 5 and 11). Note two points on the trend line are in the same position

Data presented in Tables 5 and 11 on the [Fe(II)(Por)L(CO)] complexes allow the relationship between the p.q.s. values of the opposite axial ligand and the CO-stretching frequency to be studied (see Fig. 8).

It is apparent that there is a good correlation between the CO stretch and the p.q.s value of the other axial ligand with the strongest bonding ligands being associated with the highest CO-stretching frequencies. The correlation is good although data from complexes of four different porphyrins were used. We note that a similar relationship between the *ΔE*_*Q*_ values and the CO-stretching frequencies was reported for five of these complexes [111]. Clearly the origins of the two relationships are linked. The strength of the nitrogenous axial ligand bond to the Fe(II) atom affects the strength of the Fe(II) bond to the CO molecule which in turn affects the C–O bond and this is seen in the variation of the stretching frequency. It is apparent in the plot that there is a noticeable exception in the complex that contains the most sterically inhibited axial ligand (1,2-MeIm), the few known crystal structures of this complex show unusually long N-Fe(II) axial bonds; such weaker axial bonding will result in the Fe(II) bonding to CO to become much stronger with concomitant weakening of the C–O bonding and then explain the much lower stretching frequency of the C–O bond. This is apparent in the observed smaller Fe(II)-CO bond [108].

Scheidt et al. [147–152] have stressed the importance of the binding of diatomic molecules such as O_2_, CO and NO to haem proteins for mammalian physiology. In addition, they have pointed out that the binding of nitric oxide (NO), nitrite (NO_2_^−^) and nitrate (NO_3_^−^) to haems is involved in important denitrification processes. They have reported the crystal structures and Mössbauer parameters of several (nitro)iron(II) porphyrin complexes. (See Tables 12 and 13) [147–152]. Our p.q.s. calculations on these complexes can give further insight into the bonding interaction between these biologically significant small molecules and haems.

**Table 12:** Crystal structure data for low spin [Fe(II)(Por)(NO_2_)L] complexes and [Fe(II)(Por)(NO)L] complexes

Compound	N(NO_2_)-Fe or N(NO)-Fe Å	N_Por_-Fe Å	Fe-L_Ax_ Å	Δ^*^	Refs
[Fe(II)(TpivPP)(NO_2_)(CO)]^−^ molecule(1)	2.006(4)	1.992(7)	1.782(4)	0.08^a^	[147]
[Fe(II)(TpivPP)(NO_2_)(CO)]^−^ molecule(2)	2.009(4)	1.997(6)	1.789(5)	0.05^a^	[147]
[Fe(II)(TpivPP)(NO_2_)(PMS)]^−^	1.937(3)	1.990(6)	2.380(2)	0.04^b^	[148]
[Fe(II)(TpivPP)(NO_2_)(py)]^−^	1.951(5)	1.990(15)	2.032(5)	0.01^c^	[148]
[Fe(II)(TpivPP)(NO_2_)(NO)]^−d^	2.086(8)	1.988(6)	1.792(8)	0.10^e^	[149]
[Fe(II)(TpivPP)(NO_2_)(NO)]^−d^	2.080(8)	1.992(6)	1.774(8)	0.08^e^	[149]
[Fe(II)(TpivPP)(NO_2_)(NO)]^−f^	2.060(7)	1.986(6)	1.840(6)	0.09^e^	[149]
[Fe(II)(TPP)(1-MeIm)(NO)]^g^	1.743(4)	2.008(12)	2.180(4)	0.07^h^	[150, 151]
[Fe(II)(TPP)(1-MeIm)(NO)]^i^	1.750(2)	2.008(13)	2.173(2)	0.04^h^	[151]
[Fe(II)(TPP)(4-MePip)(NO)]^g^	1.721(10)	2.004(9)	2.328(10)	0.09^j^	[151, 152]
[Fe(II)(TPP)(4-MePip)(NO)]^i^	1.7517(19)	2.008(8)	2.2851(19)	0.06^j^	[151]

*Displacement of the metal atom from the 24 atom porphyrin plane towards the axial ligand specified in each case

^a^Towards CO. PMS = pentamethylene sulphide

^b^Towards PMS

^c^Towards py

^d^Orientation of axial ligands perpendicular

^e^Towards nitrosyl

^f^Orientation of ligands parallel

^g^Structural data at 293 °K

^h^Towards 1-MeIm

^i^Structural data at 100 °K

^j^Towards 4-MePip

**Table 13:** ^57^Fe Mossbauer data and p.q.s. values for low-spin [Fe(II)(Por)(NO)_2_L] complexes and [Fe(II)(Por)(NO)L] complexes

Compound	*δ*_obs_ mm s^−1^	*δ*_calc_ mm s^−1^	*ΔE*_Qobs_ mm s^−1^	*ΔE*_Qcalc_ mm s^−1^	T °K	Refs
[Fe(II)(TpivPP)(NO_2_)(CO)]^−^	0.27	0.32	0.32		298	[147]
[Fe(II)(TpivPP)(NO_2_)(CO)]^−^	0.37	0.39	0.28	0.26	4.2	[147]
[Fe(II)(TpivPP)(NO_2_)(PMS)]^−^	0.51		1.18	1.18	4.2	[148]
[Fe(II)(TpivPP)(NO_2_)(py)]^−^	0.50	0.49	0.93	1.00	4.2	[148]
[Fe(II)(TpivPP)(NO_2_)(NO)]^−a^	0.31	0.31	1.78		100	[149]
[Fe(II)(TpivPP)(NO_2_)(NO)]^−b^	0.44		1,20		4.2	[149]
[Fe(II)(TPP)(1-MeIm)(NO)]	0.33	0.36	0.796	0.80	293	[151]
[Fe(II)(TPP)(1-MeIm)(NO)]	0.44	0.43	0.73		4.2	[151]
[Fe(II)(TPP)(4-MePip)(NO)]	0.35	0.36	0.94	0.98	293	[151, 152]
[Fe(II)(TPP)(4-MePip)(NO)]	0.46	0.43	0.91		4.2	[152]

^a^Orientation of axial ligands perpendicular

^b^Orientation of axial ligands parallel

The first six complexes presented in Tables 12 and 13 are all picket fence compounds of the general formula [Fe(II))(TpivPP)(NO_2_)L]^−^ (where L = CO, PMS, py or NO). For the first four complexes p.q.s. values for CO of − 0.83 mm s^−1^; py of − 0.46 mm s^−1^, and TpivPP = − 3.07 (as in Tables 6 and 7) yield p.q.s. values of − 0.57 mm s^−1^ (a value of 0.52 mm s^−1^ was reported previously [63]) for NO_2_^−^ and − 0.37 mm s^−1^ for the PMS ligand (a value close to those of the sulphide ligands we discussed earlier in this paper). This p.q.s. value of − 0.57 mm s^−1^ is more negative than those of the aliphatic amines and as we will see below is very dependent on the second axial ligand present.

For the two [Fe(II)(TPP)(NO)L] complexes (where L = 1-MeIm or 4-Mepip) the p.q.s. value for NO is − 0.665 mm s^−1^. This p.q.s. value is more negative than those of all the N bonding ligands we have discussed earlier in this work. We suggest this value is reflecting the presence of the unpaired electron present on the nitrosyl ligand. We also note that the low-spin [Fe(II)(Por)(NO)L] the Fe(II) atom is displaced towards the NO from the porphyrin plane by around 0.1 Å [147, 148]. This movement may be caused by the attraction of the unpaired electron for the Fe(II) cation.

When the p.q.s. values derived above for NO_2_^−^ and NO are substituted into [Fe(II)(TpivPP)(NO_2_)L]^−^ (where L = NO), then the calculated values match the observed values poorly for both structures. This is a problem and is worthy of further discussion. It is apparent from the structural data presented in Table 12a that the greatest variation in the bond lengths is in the length of the Fe–N (NO_2_) bond. For [Fe(II)(TpivPP)(NO_2_)(NO)]^−^ the Fe–N (NO_2_) bond lengths are 2.086(8) Å and 2.060(7) Å in the two different molecules in the structure (Molecules (1) and (2) in Table 12a [148]. These distances are about 0.12 Å longer than in the [Fe(II)(TpivPP)(NO_2_)L]^−^ (where L = PMS and py)[147] and 0.06 Å longer than in the L = CO [146] complexes. Scheidt et al. [147] suggested that the NO_2_^−^ ligand can vary its bonding more easily than the NO ligand. It is noteworthy that the Fe(II) cation in each case is displaced towards:- the PMS by 0.04 Å, the py by 0.01 Å and the CO by 0.07 Å (average of two forms). These values again suggest that the Fe–N (NO_2_) bonding is weaker than that of Fe–N O when present as an axial ligand. If we assume the greatest change in the bonding around the Fe(II) atom in [Fe(II)(TpivPP)(NO_2_)(NO)]^−^ is in the Fe–N (NO_2_)^−^ bond, then we can keep the previous p.q.s. values for NO and (TpivPP) and deduce a new p.q.s. value for the NO_2_^−^ ligand in the complex with the parallel ligands manifesting a *ΔE*_*Q*_ value of 1.20 mm s^−1^ to be − 0.305 mm s^−1^. But on closer examination the Fe–N (NO) bond is longer than in the two [Fe(II)(TPP)(NO)L] complexes (where L = 1-MeIm or 4-Mepip) by around 0.10 Å, so it is likely that the p.q.s. value for this ligand should also be reduced a little with the consequence that the p.q.s. value for the NO_2_^−^ ligand may be closer to about − 0.36 mm s^−1^ and that for the modified NO to around − 0.58 mm s^−1^. In contrast the form that has the perpendicular arrangement of the axial ligands also has the longer Fe–N (NO_2_)^−^ bond of 2.086(8) Å but a shorter Fe–N (NO) bond and its *ΔE*_*Q*_ value is 1.78 mm s^−1^, this would then be expected to have different p.q.s. values again, in fact the p.q.s. for the NO ligand in this complex might have been expected to be larger than that in the parallel complex and the p.q.s. of the NO_2_^−^ ligand would be expected to be smaller. The problem is that as the observed *ΔE*_*Q*_ value of 1.78 mm s^−1^ is large and at the maximum size for a low-spin Fe(II) complex “vide infra”. The calculations show that the combined p.q.s. values of the two axial ligands can only be − 0.605 mm s^−1^; so for the p.q.s. the value for the NO to be − 0.59 then the p.q.s. value for the NO_2_^−^ ligand needs to be closer to about − 0.01 mm s^−1^ or less, which is not very satisfactory. This clearly indicates that there is much more going on in the bonding in the perpendicular complex.

In the treatment of the low-spin octahedral [Fe(II)(Por)L_2_] and related complexes studied in this work we have chosen to neglect the effect of charges on the ligands or indeed the presence of extra electrons close to the bonding ligands. Such electrons or charges will clearly affect the EFG and hence the value of *ΔE*_*Q*_._._The unpaired electron on NO and the negative charge on the NO_2_^−^ ligand may be expected to have a significant effect on the Fe(II) in these complexes depending on their location/geometrical position relative to the Fe(II). Scheidt and co-workers [147, 148, 150] have studied the Mössbauer spectra of these NO complexes in detail but have not been able to fully explain the spectra in regard to both their structure and bonding. They state that it is the nitrosyl ligand that dominates the bonding in the [Fe(II)(TpivPP)(NO_2_)(NO)]^−^ complexes [147] and our p.q.s. values/findings agree with this interpretation. They do find evidence of tilting in the axial bonds in these ring ruffled structures, and we have discussed situations above where the symmetry is no longer exactly D_4h_, yet it appears that the model works so long as the distortion from this symmetry is not great. Perhaps in the case of the [(Fe(II)TpivPP)(NO_2_)(NO)]^−^ complex where the ligands are perpendicular the model is no longer valid. However, as we discussed above, it is the electronic environment that is important and small changes/distortions in the crystallographic environment may only have small effects on the electronic environment and not manifest as large changes in the EFG and thus not affect the correlations we are presenting. We note that Scheidt et al. [148] suggest that the large *ΔE*_*Q*_ value of 1.78 mm s^−1^ is halfway between that of the other form and the five-co-ordinate complex.

The p.c.s. values for the two [Fe(II)(TPP)(NO)L] complexes (where L = 1-MeIm or 4-Mepip) for both the NO ligand and TPP macrocycle in the complexes need to be deduced. NO is a good π-accepting ligand, but of course it can also donate to the Fe(II) and in some cases this donation can include an entire electron. As a starting point if we just look at NO as a good π-accepting ligand like or better than CO (it is to be noted that Bancroft et al. [73] found a p.c.s. value of − 0.20 mm s^−1^ compared to a value of 0.0 mm s^−1^ for CO), then we would expect it to have a negative value in these TPP complexes and the TPP ligand also to have a value close to the value it has in the presence of CO in low-spin six-coordinate [Fe(II)(Por)(CO)L] complexes. So, if TPP has a p.c.s. value of 0.18 mm s^−1^ then the calculated *δ* value for these complexes in Table 13 would be too small. The alternative is to use a p.c.s. value of 0.36 mm s^−1^ (the value it has in the presence of more normal nitrogenous ligands), then a value of − 0.1 mm s^−1^ for the p.c.s. value of NO results. This value can be verified by considering the values resulting from [Fe(II)(TpivPP)(NO_2_)(NO)]^−^; to do this a p.c.s. value for NO_2_^−^ needs to be calculated. Using the [Fe(II)(TpivPP)(NO_2_)L]^−^ (where L = CO, PMS, py or NO) complexes the p.c.s. value can be deduced. The first of the complexes is [Fe(II)(TpivPP)(NO_2_)CO]^−^; using a p.c.s. value of 0.29 mm s^−1^ for TpivPP then NO_2_^−^ has a p.c.s. value of 0.05 mm s^−1^. This value also fits the [Fe(II)(TpivPP)(NO_2_)py]^−^ complex. Substituting the derived p.c.s. values for NO and NO_2_^−^ and a p.c.s. value of 0.29 mm s^−1^ for TpivPP into [Fe(II)(TpivPP)(NO_2_)(NO)]^−^ gives a *δ*_calc_ value of 0.31 mm s^−1^_,_ which is a good fit to the observed *δ* value at 100°K, again showing the versatility of this approach.

Finally, there are five-reported [Fe(II)(OEP)(L)CS] complexes that have been studied using Mössbauer spectroscopy [152]. These are listed along with their known crystal structure details [152] in Table 14. We have calculated the *ΔE*_*Q*_ values for these complexes, three show excellent agreement with the measured values, the pyridine complex is not too bad but the calculated value for the 4-CNpy complex is the worst match of all that we have presented in this work. This we find surprising in the light of the match that we have had using the p.q.s. value of − 0.47 mm s^−1^ that we used for this ligand and indeed all the 4-substituted pyridines. We feel that this merits further discussion; in their work on these complexes Scheidt et al. [152]. have presented plots of the *ΔE*_*Q*_ values against the L ligand pKa’s and against the infrared stretching frequencies of the CS ligand, (both the correlations were reasonable), however, we note that if 4-CNpy was bonding to the iron via the N of the CN rather than that of the pyridine ring, the correlations would not be affected much. Others [74, 77] have given a p.q.s. value for MeCN of − 0.43 mm s^−1^; such a value would in our calculations generate a calculated *ΔE*_*Q*_ value of 0.64 mm s^−1^ which is a move in the right direction. It should be noted that in the porphyrin complexes we found the value for CN^−^ is around 15% lower than in the complexes the previous workers used [74, 77], then a p.q.s value of 0.37 mm s^−1^ would generate a *Δ*E_Q_ value of 0.74 mm s^−1^. Of course, this is based on the value for MeCN, which is more electron rich than 4-CNpy, so a p.q.s. value closer to 0.30 mm s^−1^ would give a *Δ*E_Q_ value of 0.88 mm s^−1^ which would be a good match for the observed value for [Fe(II)(OEP)(4-CNpy)CS] in Table 14. If the bonding is shown in the future to be via the N of the CN then the power of this approach is again illustrated. Finally, in agreement with the findings of Scheidt et al. [152] all the *ΔE*_*Q*_ values we calculated are positive for the [Fe(II)(OEP)(L)CS] complexes in Table 14.

**Table 14:** Crystal Structure data, ^57^Fe Mossbauer data and for low-spin [(Por)Fe(II)(CS)_2_L] complexes (where L = nitrogenous ligand)^a^

Compound	C(CS)-Fe Å	N_Por_-Fe Å	Fe-L_Av_ Å	*δ*_obs_ mm s^−1^	*δ*_calc_ mm s^−1^	*Δ*E_Q obs_ mm s^−1^	*Δ*E_Qcalc_ mm s^−1^	T°K
[(OEP)Fe(II)(4-CNPy)(CS)]				0.12	0.10^b^	0.90	0.52^c^	293
[(OEP)Fe(II)(Py)(CS)]	1.707(2)	2.005(2)	2.1469(18)	0.13	0.13	0.67	0.54	293
[(OEP)Fe(II)(Pip)(CS)]				0.14	0.14	0.65	0.65	293
[(OEP)Fe(II)(4-NMe_2_Py)(CS)]				0.15	0.14	0.497	0.50	293
[(OEP)Fe(II)(1-MeIm)(CS)]	1.703(4)	2.001(4)	2.112(3)	0.12	0.14	0.47	0.46	293

^a^All data taken from ref. [153]. The *δ*_obs_ mm s^−1^ data are relative to stainless steel at room temperature

^b^Calculated and discussed in text using a p.c.s. value of 0.04 mm s^−1^ for 4CN-py

^c^Discussed in text and changed to 0.88 mm s^−1^ if bonding through N atom of the CN substitute

The *δ*_obs_ mm s^−1^ values of all five-reported [Fe(II)(OEP)(L)CS] complexes are very small, in fact the smallest yet recorded for low-spin [Fe(II)(Por)L_2_] complexes as reported by Scheidt and co-workers [152]. They suggest that *δ*_obs_ mm s^−1^ values are comparable to values found for [Fe(Por)L_2_] complexes where the Fe atom is in either the formal oxidation state of 3 or of 4, especially those with good π-accepting ligands [122]. From this they state “it is tempting to suggest that the extremely low value of the *δ* is due solely to the π-accepting character of the axial thiocarbonyl ligand. However, decreases in the *δ* in a similar series of complexes can be related to both increased σ-donating and π-accepting character of the ligands” [153]. They also state “that Mössbauer spectra for the six-coordinate thiocarbonyl complexes provide evidence for the importance of σ-bonding as well as π-bonding for contributions to the extremely low *δ*” [152]. We tend to go along with their first suggestion as π-accepting character of the axial ligands is a major factor. Scheidt and co-workers [152] also state that the *δ*_obs_ mm s^−1^ values that increase by ~ 0.15 mm/s between members of the series ((CS),L) > ((CO),L) > two neutral nitrogen donor ligands are dominated by decreasing Fe → L(Axial) π-bonding in the order CS > CO > nitrogen donor. We will now show that this is indeed the case by calculating the p.c.s. value for CS. To calculate p.c.s. values for all the ligands in these complexes is not simple and three assumptions are necessary:- firstly we assume that the p.c.s. values of the other axial ligand does not change; secondly we assume that the p.c.s. value for OEP in these complexes will be smaller than it is for the [Fe(II)(OEP)(CO)L] complexes; thirdly we must assume that the p.c.s value for CS is smaller (more negative) than that for CO. If we assume a p.c.s. value for OEP in the complexes of 0.14 mm s^−1^ then the p.c.s. value for CS is − 0.08 mm s^−1^. Obviously the p.c.s. value for CS would be smaller if that for OEP were larger, so we can only get a feel for the p.c.s. value. So, if OEP p.c.s. value was 0.18 mm s^−1^ then CS would have a p.c.s. value of − 0.12 mm s^−1^. (We used the latter values in the calculations in Table 14). In our calculations we used a p.c.s. value for 4CN-py of 0.04 mm s^−1^ in agreement with the value of 0.03 mm s^−1^ for MeCN we derived from Refs. [154–156].

Earlier herein we demonstrated that the p.c.s. values were different for porphyrins in [Fe(II)(Por)L_2_] complexes and porphyrins in [Fe(II)(Por)(CO)L] complexes and discussed why this was the case. This finding is in keeping with the idea that a porphyrin can behave as an “electron sink” allowing it to vary its bonding to the Fe depending on the bonding needs of some axial ligands. Moreover, this is the first quantitative evidence for this property of macrocyclic ligands. It is now obvious that axial ligands that have very good π-accepting character should affect the p.c.s. value of the porphyrin. The problem is that many data are necessary to allow good evaluation of the porphyrin p.c.s. values and this is not always available in the literature. It should be noted that there is no previous report of different p.c.s. values for the same ligand depending on what other ligands were present. This is due to the fact that previous studies were all based on complexes wherein all the ligands could move independently to take up their most preferred position and it is only in macrocyclic ligands where this is not possible. In the limited cases where macrocyclic ligands were reported for example for [Fe(II)(pc)] complexes there was only one compound [83] or where there were many[79–81] the investigators did not allow the p.c.s. value to vary.

If we rank the p.c.s. values of the axial ligands when bound in low-spin six-coordinate [Fe(II)(Por)L_2_] complexes derived in this work we find CS < NO < CO < CN^−^ < PPh(EtO_2_) < P(MeO)_3_ < PBu_3_ < PEt_3_ < PMe_3_ < py = NH_3_ < pip = Im < Por/4. This is in the inverse order to their position in the Fajans-Tsuchida spectrochemical series [157–161]. A correlation between the p.c.s. values for some of these ligands and the spectrochemical series was previously found by Bancroft et al. [73, 83]. They explained that just as the spectrochemical series ranking should increase with an increase in σ-bonding from ligand to metal and π-bonding from metal to ligand(M → L) so the p.c.s. values should decrease [73, 83]. They had difficulties with the correlation as they could not use the crystal-field splittings from Fe(II) complexes as the spectra were often masked by large charge transfer bands. To get over the problem they made use of data from Co(III) complexes. The fact that many of the p.c.s. values deduced in this work are in close agreement to those found by Bancroft et al. [73–75, 83] is instructive as our values arose where there was always a porphyrin ligand in the complex. The later as discussed herein, both imposes a geometry and restricts/manipulates the chemistry of the Fe(II) atom; although the p.c.s. value of the Por always contributing to the total *δ*_calc._ value in most cases it does not cause the p.c.s. values of the axial ligands to change with the main exception being for axial CO ligands as we discussed earlier. In the case of axial CO ligands. the p.c.s. values of the porphyrins did change and their position (for Por/4) in the above series would be close to that of PEt_3._

If we order the p.q.s. values of the axial ligands when bound in low-spin six-coordinate [Fe(II)(Por)L_2_] complexes we also find CS < CO < CN^−^ < Por/4 < PPh(EtO_2_) < P(MeO)_3_ < PMe_3_ < NO < NO_2_^−^ < PEt_3_ < PBu_3_ < Im = NH_3_ < py < pip, although there are some differences to the positions found for the p.c.s. values this series is also in the inverse order to their position in the spectrochemical series.

**Fig. 9:**
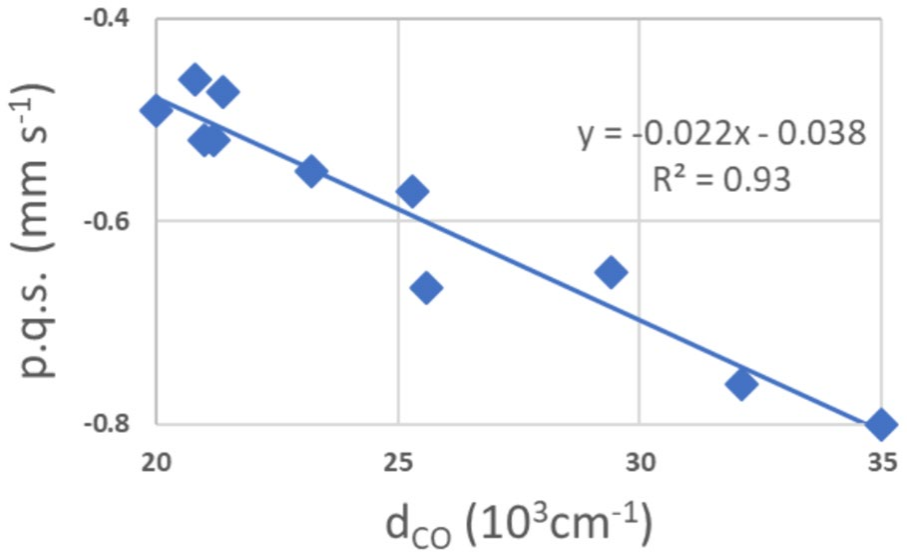
Plot of *d*_Co_/10^3^ cm^−1^ (where *d*_Co_ is the *d* value of each ligand for different complexes of a given metal and can be evaluated as in ref. [163] from where the *d*_Co_ values are taken). *d*_Co_ can be correlated to Jorgenson’s *f* (ligand) value [162] against the p.q.s. values of eleven axial ligands(derived in this work (see ligand list and data in Table 15)

Herein we make use of the *d* parameter proposed for the spectrochemical series of ligands in the mixed ligand complexes of d^6^ metals that allows the prediction of low energy d-d absorption bands in a d^6^ complex [162]. Shimura [162] deduced a series of *d* (in 10^3^ cm^−1^) values for over 100 monodentate ligands and many bidentate ligands for Co(III) and explained how they could be adapted for any d^6^ metal. We have used some of these values shown in Table 15 and plotted them in Fig. 9 against p.q.s. values derived for the same ligands in this work (unfortunately only eleven ligands were common to both lists). However, a very reasonable correlation is observed.

**Table 15:** Ligand p.q.s. values from Tables 6 and 7 and *d* (10^3^ cm^−1^) values from Ref. [162]

Ligand	p.q.s. mm s^−1^	*d*(10^3^ cm^−1^)	Comments
CO	− 0.80	35.0	
CN^−^	− 0.75	32.1	
P(OMe)_3_	*− *0.65	29.4	
PMe_3_	*− *0.655	25.6	
NO_2_	*− *0.57	25.3	
PPh_3_	*− *0.55	23.2	p.q.s. value taken from PBu_3_ (see Table 9)
(CH_2_NH_2_)_2_	*− *0.472	21.4	
Im	*− *0.52	21.2	
NH_3_	*− *0.52	21.0	
Py	*− *0.46	20.8	
MeNH_2_	*− *0.49	20.0	p.q.s. value taken from PrNH_2_ (see Table 9)

This correlation has not been previously reported and is in our view expected as the p.q.s. values are deduced from the *ΔE*_*Q*_ values of the [Fe(II)(Por)L_2_] complexes. The *ΔE*_*Q*_ value is a measure of the EFG around the Fe(II) atom and this results from the valence electrons. As stated earlier in these diamagnetic low-spin [Fe(II)(Por)(L)_2_] complexes the major contribution to V_zz_ arises from an imbalance in electron densities in the *d*_x_^2^_-y_^2^ and *d*_z_^2^ orbitals[101, 120]. Thus each axial ligand will have a different effect on these two orbitals depending on its σ- and π-bonding electron contribution to these orbitals and from the correlation shown in Fig. 9 (though not perfect) it appears that the p.q.s. values reflect the contribution each ligand makes to the crystal-field splitting.

Earlier in the discussion to obtain p.q.s. values for CO in the [Fe(II)(Por)L(CO)] complexes we assumed that the p.q.s. value for the other axial ligand did not change on binding CO. This of course implies that any trans effects between the axial ligands do not affect the field gradients.

**Fig. 10:**
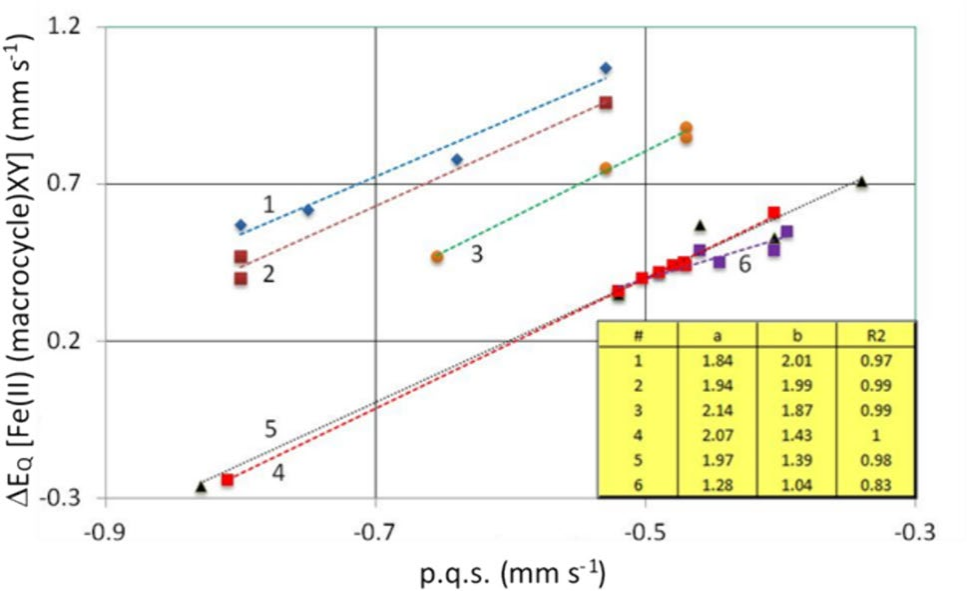
Plot of the *ΔE*_Q_ values of the [Fe(II)(Por)XY] complexes (where the X axial ligand does not change and only the Y axial ligand changes) against the Y ligand’s p.q.s. value. Figure 10 differs from Figs. 2 and 4 (which plotted [Fe(II)(Por)L_2_] complexes against the p.q.s values as it plots [Fe(II)(Por)XY] complexes against the p.q.s. value of the Y ligand. Trend lines for the various Fe(II)porphyrin complexes are indicated by numbers. 1 [Fe(II)(TPP)XY]; 2 [Fe(II)(OEP)XY]; 3 [Fe(II)(TMP)XY]; 4 [Fe(II)(TPP)XY]; 5 [Fe(II)(PMXPP)XY] and 6 [Fe(II)(PPIX)XY]. The insert shows the coefficients of the trend lines for the various ligand groups. Apart from trend line 6 the correlation coefficients are > 0.95

Figure 10 displays a plot of the *ΔE*_*Q*_ values of the [Fe(II))(Por)XY] complexes against the p.q.s. values of the Y axial ligands. Figure 10 differs from Figs. 2 and 4 (which plotted [Fe(II)(Por)L_2_] complexes (where L is the axial ligand) against the p.q.s values as it plots [Fe(II)(Por)XY] complexes (where X and Y are different axial ligands) against the p.q.s. value of the Y ligand. In Fig. 10 only the p.q.s. value of the Y ligand is used in the plot and the result is a series of lines where the slope is only half that seen for the plots in Figs. 2 and 4. The reason for this is that for the [Fe(II)(Por)XY] complexes Eq. (4) can be rewritten as:

6 $$\Delta {E}_{Q}=a \text{p}.\text{q}.\text{s}.\left(X\right)+ a \text{p}.\text{q}.\text{s}.\left(Y\right)+ b \text{p}.\text{q}.\text{s}.\left(\text{macrocycle}\right),$$

where the coefficients *a* and *b* are 2 and -4 respectively in Eq. (6), and *Y* is the axial ligand that varies.

If the macrocycle is constant, say a porphyrin for a series of [Fe(II)(Por)XY] complexes where *X* is also constant then only *Y* can vary, then the second and third terms in Eq. (6) can be combined into the same Constant and Eq. (6) becomes:

7 $$\Delta {E}_{Q}=a \text{ p.q.s. }\left(Y\right)+\text{Constant}.$$

**Fig. 11:**
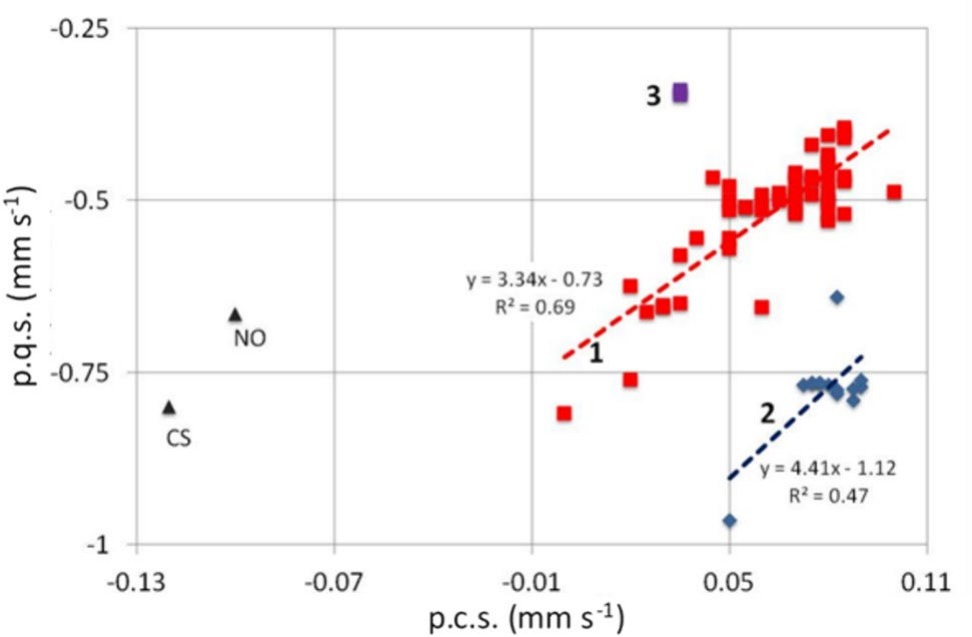
Plot of p.q.s. values of the axial ligands derived from [Fe(II)(Por)L_2_] and [Fe(II)(Por)XY] complexes against the p.c.s. values for these ligands. All the red squares indicate monodentate ligands (fitted by trend line 1), whereas the blue diamonds present the values for ¼ of the values for the pc and Por ligands (fitted by trend line 2). The p.c.s. values used for pc and the Por ligands are those derived when the axial ligands are the L = nitrogenous bases. In the presence of stronger bonding ligands the p.c.s. values of the pc and Por ligands would move these blue points to the left. The two most sterically hindered nitrogenous ligands (2-MeHIm and 1,2-Me_2_Im) are shown as purple squares labelled as 3. The positions of the ligands CS and NO are shown in grey

In Eq. 7$a$ has a value of 2, whereas in Eq. 5 it has a value of 4 so the slope in Fig. 10 will be only half that in Figs. 2 and 4. However, just as before, but in this case from Eq. 7 it follows that *ΔE*_*Q*_ for a series of porphyrin complexes of the type [Fe(II)(Por)XY] with varying *Y* depends only on the value of p.q.s. (*Y*). This is apparent in Fig. 11. For other macrocycles, the Constant will be different, establishing parallel lines, because the coefficient *a* is the same for a set of L ligands with a given macrocycle. The fact that the correlation coefficients are close to one vindicates our finding that the EFG the Fe(II) atom experiences from a given porphyrin is invariant [or largely constant] and the changes in the field gradient that cause changes in the *ΔE*_*Q*_ values are due to the changes (in bonding) in the axial ligands. Moreover, as the p.q.s. of the *X* is also constant this shows that from the point of view of the EFG the axial ligands act independently of each other.

In Fig. 10 we provide evidence to back this assumption, the figure presents plots *ΔE*_*Q*_ values of the [Fe(II)(Por)*XY*] complexes where the *X* axial ligand does not change and only the *Y* axial ligand changes. In the figure data are presented for six series of complexes:-

1. [Fe(II)(TPP)XY] where *X* = 1-MeIm and *Y* = 1-MeIm, CO, CN or NO;
2. [Fe(II)(OEP)XY] where *X* = 1-MeIm and *Y* = 1-MeIm, CO or CS;
3. [Fe(II)(TMP)XY] where *X* = PMe_3_ and *Y* = PMe_3_, 4-NMe_2_py, 4-CNpy or 1-MeIm;
4. [Fe(II)(TPP)XY] where *X* = CO, *Y* = CO, py, pip, 1-MeIm or 1,2 -MeIm;
5. [Fe(II)(PMXPP)XY] where *X* = CO, *Y* = Morph, pip, py, Pydn or Im;
6. [Fe(II)(PPIX)XY] where *X* = CO, *Y* = CO, pip, Im, EtNH_2_, (NH_2_CH_2_)_2_, but, s-but, 4-Mepy, 2-MeIm or 2-Mepy.

In each case a trend line has been fitted and it is apparent that linear relationship *R*^2^ values are very reasonable. The *X* ligands are a mix of purely σ-donor ligands and ligands that are both σ-donors and π-donors or σ-donors and π-acceptors. Clearly as these ligands all lie on the same-trend lines for a given [Fe(II)(Por)XL] complex this is evidence that there are no additive bonding affects between the axial ligands that affect the EFGs. This therefore is evidence that in the low-spin six-coordinate [Fe(II)(Por)XY] each axial ligand affects the field gradient independently. In contrast the p.c.s. values which are a measure of the “s” electron density at the nucleus due both from σ-donation (s-electron density) and π-donator/acceptor properties (p- and d-electron shielding) of the ligands at the nucleus do vary for the Por ligands when ligands such as CO are present. This variation in the p.c.s. values (depending on change of axial ligand) is often directly apparent in physical properties such as bond lengths.

Figure 11 displays a plot of the p.q.s. values against the p,c.s. values of all the axial ligands considered herein. This plot is an extension of Fig.e 1 and contains the values for all the axial ligands in addition to the nitrogenous ligands considered in the earlier figure. It is apparent in Fig. 11 that all the axial ligands shown as red squares lie around the trend line 1; this trend line only becomes apparent as non-nitrogenous ligands are added and is obviously different than the line in Fig. 1 (moreover the correlation is of course better when the two sterically hindered ligands are not included). All these axial ligands have p.q.s. values in the range − 0.8 to − 0.4 mm s^−1^ and p.c.s. values in the range 0.0 to 0.1 mm s^−1^. The two very sterically hindered axial ligands (2-MeHIm and 1,2-Me_2_Im) have p.c.s. values of − 0.035 mm s^−1^ and p.q.s. values of 0.3475 mm s^−1^ and 0.34 mm s^−1^ respectively. Only two of these axial ligands that have p.q.s. values of around − 0.8 and p.c.s. values of 0.0 to − 0.05 mm s^−1^ are strong enough ligands to change the sign of V_zz_ in the low-spin [Fe(II)(porphyrin)L_2_] complexes. This is apparent in Fig. 11 where their positions are below those of the porphyrin ligands (but not below Pc where we did not find a change in the sign of V_zz_). This is obvious as 4 x − 0.8 mm s^−1^ is larger (in absolute value) than each of the porphyrins 4 x (¼Por) p.q.s. The p.q.s. values of the pc/4 and the porphyrins/4 (blue diamonds) in Fig. 11 are all on trend and all but OMBTP have values that are close to -0.08 or lower as discussed earlier. Pc, which has the most negative p.q.s. value, is lower than all the axial ligands so all its 6-co-ordinate low-spin Fe(II) complexes will have positive V_zz_ values. It follows that if a monodentate ligand has a p.q.s. value below that of a porphyrin (as laid out in Fig. 11), then it will be strong enough to change the sign of V_zz_. An example of this can be seen for OMBTP from its position as indicated by the highest blue diamond in Fig. 11: any axial ligand with a p.q.s. value larger than − 0.65 mm s^−1^ should be able to change the sign of its’ V_zz_. Unfortunately, there is no complex of [Fe(II)(OMTBP)L_2_] known where the L ligand has this p.q.s. value; however, the complex [Fe(II)(OMTBP)(1-MeIm)(CO)] has two axial ligands where the average p.q.s. value is − 0.66 mm s^−1^ and a reported *ΔE*_*Q*_ value of 0.0 mm s^−1^ similarly the complex [Fe(II)(OMTBP)(pip)(CO)] has an average axial ligand value of − 0.6 mm s^−1^ and a reported *ΔE*_*Q*_ value of 0.2 mm s^−1^ [82].

From their positions in Fig. 11 it is apparent that the properties of the nitrogen atoms of the porphyrins are very different to the monodentate axial ligands. As stated earlier, there was no a-priori reason for the p.q.s. values and the p.c.s. values of the ligands to fall on a common trend line: however, it is worth noting that Bancroft et al. [73] also found such a relationship. The main reason we see differences for both the p.q.s. value and p.c.s. values compared to those reported by Bancroft et al. [73] is that in these [Fe(II)(Por)L_2_] and [Fe(II)(Por)XY] complexes the Por ligand dominates/dictates the Fe(II) chemistry and by imposing a field around the iron atom, which in the case of the p.q.s. values does not differ from one complex to the next. This imposed field restricts/manipulates the bonding properties of the Fe(II) in the haem. No equivalent field was present/imposed on the Fe(II) complexes considered by Bancroft et al. [73], so all their ligands could bind to Fe(II) independently and freely subject only to steric restrictions. In contrast our values are derived specifically for these [Fe(II)(Por)L_2_] and [Fe(II)(Por)XY] complexes and provide insight into what controls the chemistry in these complexes.

The ligands CS and NO lie a distance to the left of the other monodentate ligands. We considered the complexes that contained these ligands in the earlier discussion. In the case of NO this has the second most negative p.c.s. value, though the value we found is less negative than that reported by Bancroft et al. [73]. We believe this is due to both the presence of the porphyrin ligand and also to the location of the unpaired electron that before the complexes form was confined to the NO molecule. The nitrite ligand also was shown to vary its’ chemistry herein and by previous workers from structural considerations [147, 148]. Scheidt et al. [147, 148] have suggested that the variation in π-bonding from the Fe(II) to the N-atom of the nitrite ligand is dependent on the other axial ligand present; they state that the nitrite ligand is a very strong π-acceptor ligand [147]. We do not disagree with this, but we found a maximum p.q.s. value of 0.58 mm s^−1^ for this ligand and a p.c.s. value of 0.05 mm s^−1^ and it is plotted with these values in Fig. 11 and thus does not stand out from the crowd.

### Consequences of nitrogen tetradentate ring formation

A nitrogen tetradentate macrocyclic ring has four nitrogen atoms at its’ centre capable of bonding to a metal cation. The macrocyclic ring can be aliphatic, aromatic or partially aromatic. Changing the bonding in the ring will affect the nitrogen to central cation distance. This distance and the electrons available for bonding on the nitrogens will give rise to the p.q.s. and p.c.s. values of the nitrogen tetradentate ligand. The obvious question that arises from this is- Is it possible to quantitatively understand the effects of systematically varying the nitrogen to iron bond distances in tetradentate ligands by making appropriate iron complexes and deriving p.q.s. values and p.c.s. values? From the work of Busch et al. [77, 78, 163, 164], on less-conjugated macrocycles bonded to Fe(II) we can gain further insight into the properties of natural haems. Busch et al. [77, 78] have reported studies on both aliphatic and partially aromatic [Fe(II)(macrocycle)L_2_] complexes. From their work and the observation that “the lower the p.c.s. value the better the σ-donor” an interesting concept arises for ligands incapable of π-bonding. This is the possibility of making σ-donating aliphatic nitrogen macrocycles that, though tetradentate could be weak or strong ligands depending on the size of the hole between the four central nitrogen atoms. In the case of weakly bonding macrocyclic ligands, the oxidation state, oxidation potential and spin state of an iron atom for such a weak ligand would then be preferentially “controlled” by the choice of the axial ligand. This concept was partially explored by Busch et al. [77, 78, 162, 163] but more from the point of view of the macrocyclic ligand.

There is evidence that this concept is correct from the Mössbauer parameters of the [13] ane N_4_, [14] ane N_4_, [15] ane N_4_ and [16] ane N_4_ aliphatic macrocycles [78], where [13] ane N4 refers to a macrocycle containing 4 N atoms and 9 C atoms. The *Δ*EQ for the octahedral low-spin [[X](ane N_4_)Fe(II)(CN)_2_] complexes (where X = 13, 14, 15, or 16) increase as the size of the central hole increases in the order [13]ane N_4_ <[14] ane N_4_ <[15] ane N_4._ Using the p.q.s. value of − 0.84 mm s^−1^ for CN^−^ (given in Ref. [78]) then it is possible to calculate approximate p.q.s. values for these ligands which are − 0.64 mm s^−1^, − 0.605 mm s^=1^ and − 0.48 mm s^−1^ for ¼[13] ane N_4_, ¼[14] ane N_4_ and ¼[15] ane N_4_ respectively showing that as the hole size increases, the p.q.s. value decreases. These values are approximated as these macrocycles possess four central nitrogen atoms that are not in a square but are tetragonally distorted and the model used in this work assumes close to perfect octahedral coordination [73–75, 83]. It is noteworthy that they found that for [(16]ane N_4_)Fe(II)(CN)_2_] the complex is high spin [78] not low spin. This fact suggests that the p.q.s. value for ¼[16] ane data for N_4_ would be substantially less than − 0.48 mm s^−1^. It is to be noted that if the p.q.s. values for CN^−^ derived in this work in the presence of porphyrin macrocyclic ligands were used, the p.q.s. values for the [Z] ane 4 macrocyclic ligands would be less negative. This is evidence for the change of the p.q.s. value of an axial ligand when a substantially different macrocyclic ligand is restricting the bonding properties of the Fe(II) atom. Similar affects were discussed for Pc compared to PPIX in the presence of CO in this work. The p.c.s. data for ¼[13] ane N_4_, ¼[14] ane N_4_ and ¼[15] ane N_4_ are 0.05 mm s^−1^, 0.06 mm s^−1^ and 0.07 mm s^−1^ respectively, thus as the σ-donor power decreases, so does the p.c.s. value. If the p.c.s. and p.q.s. values of the [(16]ane N_4_)Fe(II)(CN)_2_] complexes for *Z* = 13, 14. and 15 were plotted in Fig. 11, they would be close to those of the monodentate nitrogen bases and not close to the porphyrins. This shows that natural processes exploited the extended conjugation of the porphyrins to modify the properties of the Fe(II) in the low-spin six coordinate [Fe(II)(Por)L_2_] complexes, as other kinds of macrocycles do not have the necessary p.c.s. and p.q.s. values. In addition, it is to be noted from the positions that the [Z] ane 4 macrocyclic ligands would have in Fig.e 11, that many of the monodentate axial ligands would be below them in the figure (and thus when present in complexes with [Z] ane 4 generate overall negative *ΔE*_*Q*_ values) and thus cause low-spin [Fe(II)((Z)ane N_4_] complexes to have negative *ΔE*_*Q*_ values as observed by Busch et al. [77, 78].

Further examination of the literature shows that some work has been carried out on such macrocyclic iron complexes to try to understand the question posed here (for aliphatic [164]) and predominantly aliphatic [165, 166] macrocycles) on varying ring size. Molecular mechanics calculations on purely aliphatic macrocycles have been carried out to determine best-fit sizes for metal ions fitting into the holes in the trans conformers. The trans-form that has the best fit depends on its hole size and the size of the metal sitting in it [164]. Low-spin bis-pyridine iron(II) complexes of five different partially conjugated macrocycles have been studied [165]. It is possible to derive approximate p.q.s. values for these (approximate again because three have distorted centres, also all have asymmetric conjugation). The p.q.s. values decrease from − 0.46 to − 0.896 mm s^−1^ as the *ΔE*_*Q*_ increases from 0.0 to 1.75 mm s^−1^. Once again, the more negative p.q.s. value is associated with the smaller ring (smaller hole size, [14]-membered ring) and the better σ-donating ligand. The [16]-membered ring exhibits a spin state equilibrium.

Intermediate spin iron(II), low and high-spin iron(III) complexes of [14]-, [15]- and [16]- membered ring macrocycle compounds have been reported. The spin states were found to be dependent both on ring size and axial ligand [166, 167].

We previously referred to TAAB [77] which is the only other known conjugated macrocycle nitrogen ligand that has a symmetrical centre, though it lies well away from the porphyrins in Fig. 11, indicating that the electric field it imposes on the iron atom is also very different to that of the porphyrins. This indicates that electron donating/accepting properties is the most important property in the haem proteins and that the symmetry of the centre of the macrocycle is of secondary importance.

### Biological significance of these findings

Although it has been shown herein that all the porphyrins other than OMTBP have relatively similar bonding properties as seen from their observed p.c.s. and observed p.q.s. values, the differences observed are significant. This is evidence that the periphery groups that differ from those on naturally occurring haems are responsible for the observed differences on the porphyrin nitrogen iron bonding (when the iron is in the (II) valence state), compared to [Fe(II)(PPIX)] itself. These differences would clearly affect the performance of the active sites in enzymes, as seen when comparing the Mossbauer parameters. Obviously in the case of [Fe(II)(PPIX)] itself, its periphery groups will also have other roles in the enzymes in which this haem is found, (such as bonding to enzyme) and protecting the Fe(II) centre from the unwanted attention of other small molecules and thus may in turn influence the active sites.

From this work it is possible to get a qualitative feel for the Fe–N bonding present in porphyrins compared to the Fe–N bonding from monodentate nitrogen ligands. Linking 4 nitrogen ligands in a conjugated porphyrin ring causes the σ-bonding ability of the N-atoms to be increased as they are forced nearer to the iron(II) then would otherwise be the case. The distance is not as close as that in pc and thus the σ-donation is probably not maximised. The most likely reason for this is that the distance is critical to controlling the properties of the Fe(II) atom holding it in a form of poised state so that fine control can be easily achieved using one or two of the axial ligands. The fact that the p.q.s. value for each porphyrin does not manifest noticeable change when axial ligands change verifies that in low-spin Fe(II)porphyrin complexes the contribution the porphyrin makes to the asymmetry of the electron field around the Fe(II) atom is invariant unless other forces cause the porphyrin to deform. In contrast the fact that the p.c.s. value for each porphyrin shows variation with major change in axial ligand (for example when a nitrogenous base is substituted by a CO) is a manifestation of the electron sink properties of the porphyrin.

As the p.q.s. values for the porphyrins are almost invariant then the overall *ΔE*_*Q*_ value of each low-spin Fe(II)porphyrin complex considered herein is dictated by the magnitude of the p.q.s. values of the axial ligands. If the axial ligands are arranged in the order of the magnitude of their p.q.s. values, the relationship to the spectrochemical series is apparent. This is not surprising as the latter was derived from the field these ligands imposed on d^6^ transition metals via their bonding orbitals. From the position of CO and CN^−^ in the spectrochemical series and from the p.q.s. values derived for them herein it is not surprising that they bond so strongly to Hb and Mb,

The position of O_2_ in the spectrochemical series and the fact that herein we found that if it was bonding to Fe(II) in the porphyrins, it’s p.q.s. value would be in the range 0.03–0.14 mm s^−1^ is strong evidence in favour of the argument that in Hb it binds as superoxide to Fe(III). It is noteworthy that a recent perspective in bioinorganic chemistry suggests the jury is still out in the iron-oxygen bonding in Hb [168].

## Conclusions

We have presented a new analysis of the Mössbauer parameters of low-spin six-co-ordinate [Fe(II)(Por)L_2_] complexes, which enabled the construction of figures manifesting the relation between p.c.s. values and partial *Δ*EQ values of a wide range of axial ligands including the cases for where the two axial ligands differed.

Nature has utilised and refined macrocycle ligands such as porphyrins (in haem proteins), porphyrins (in chlorophylls) and corrins (in the B_12_ enzymes) as the reactive centres for a range of widely different roles in living systems. In the case of the porphyrins the findings discussed in this paper give insight into factors/properties inherent in these “tailored” molecules allowing them to confine and control the chemistry of the imprisoned metal atom. The understanding of the principles by which this control has evolved/developed (based on the comparison of their σ and π bonding properties with other four nitrogen centred less-conjugated macrocyclic ligands) provides new insight to improving our manipulation and design of chemical systems. The qualitative results presented and interpreted in this work enable some factors that influence bonding in a wide range of aliphatic and aromatic ligands to be appreciated.

The derived p.c.s. and p.q.s. values were shown to fit all the six-coordinated low-spin [Fe(II)(Por)L_2_] complexes (that have known Mossbauer data). These derived values allowed an overview/understanding of the chemistry of the six-coordinated low-spin [Fe(II)(Por)L_2_] complexes that cannot be grasped from isolated studies on crystal structures and Mössbauer parameters of the iron porphyrins themselves.

In this work we have been able to estimate the range of *ΔE*_*Q*_ values that can exist for low-spin [Fe(II)(Por)L_2_] compounds and shown that ligands with p.q.s. values that are less negative than ~ − 0.34 mm s^−1^ cannot form these compounds alone. They can however be present in a six-coordinated low-spin [Fe(II)(Por)LX] complex where the second ligand is CO or an equivalent strongly binding ligand. We have also shown how ligands such as CO that have p.q.s. values of ~ − 0.8 mm s^−1^ bind very strongly to [Fe(II)(Por)] compounds and can reverse the sign of the major component of the EFG. In the presence of a strongly binding axial ligand the p.c.s. value of the porphyrin was shown to vary, in contrast the p.q.s. value was invariant.

This versatile approach has herein been shown to be applicable to all the six-coordinated low-spin [Fe(II)(Por)L_2_] complexes and the derived p.c.s. and p.q.s. values of the axial ligands have been shown to be inversely related to the spectrochemical series. It was further shown that the derived p.q.s. can be directly related to:-

- Fe-N_axial_ bond lengths,
- to ^*ʋ*^_CO_-stretching frequencies (in cm^−1^) when CO is bound as an axial ligand,
- to Co(III) *d*_*Co*_/10^3^ cm^−1^ values,
- in addition, it was shown herein that the p.q.s. values of the axial ligands in the presence of an Fe(II)(Por) combination are modified compared to the values they manifest in six-coordinate complexes that contain only monodentate or bidentate ligands. This illustrates the role of the porphyrin in controlling the chemistry of the Fe(II).
- It was also shown herein that the p.q.s. value of PPIX was different to that of the other porphyrins even though the core of the porphyrins are all similar. This has important implications for living chemistry as [Fe(PPIX)] is referred to haem b and is closely related in structure to heams a and c (all three are common in a large range of natural biological molecules.

It proved impossible to obtain a reasonable fit to a p.q.s. value for the oxygen molecule from the *ΔE*_*Q*_ value reported from the Mossbauer spectrum of Hb using the p.q.s. values for PPIX and histidine for Fe(II) complexes. This is interpreted herein as evidence that the oxyheamoglobin is best formulated as containing an Fe(III) bound to a superoxide O_2_^−^ molecule.

## Experiment

We have reported both a review of the history of deriving p.c.s. and p.q.s. values in the main text of the paper and included in the text our new equation that allows the perspective from the point of view of the axial ligands, so we do not feel we need to report more of this here. All graphs were calculated using simple MS Excel methodology. We have reported many details of our experimental methodology previously, herein we will only give details of new synthetic preparations and brief details of our Mossbauer spectroscopic technique.

Haematin was purchased from Sigma and used without further purification.

The *α, β, γ, δ* – tetra substituted porphines used to prepare the new compexes reported herein were prepared from pyrrole and the appropriate aldehydes in refluxing propionic acid according to Alder et al. [169]

For diphenyl (di-p-tolyl)porphine (ph_2_Tol_2_PH_2_) a 1:1 molar mixture of benzaldehyde and p-tolaldehyde was used.

The porphines were purified by chromatography on alumina columns and subsequent recrystallisation from chloroform. TNPH_2_ has been prepared previously using this method [170].

The sulphonated porphines were prepared from the above porphines by treatment with concentrated sulphuric acid according to Fleisher et al. [171].

The naphthalene derivative was heated for 8 h and then allowed to stand for 48 h. Dark green solids were washed many times with acetone and dried.

Infrared spectra indicated the presence of both water and acetone in the solids. Chromatography did not resolve isomers.

We have previously published full details of the preparation of [Fe(II)(TPPS)] and [Fe(II)(TNPS)] in solution [61, 62].

Details of the preparation of the complexes reported herein in frozen solution have been presented in detail [172, 173].

For the new [Fe(II)(Por)(P-ligand)_2_] complexes reported in this work the ligands tri-n-butylphosphine, tri-n-butylphosphite, tri-methylphosphite and diethylphenylphosphinate were all purchased from Aldrich. The ligands were vacuum distilled under nitrogen.

All the P ligand complexes were formed in solution in the presence of [Fe(II)(PPIX)] at PH = 12.

Heamatin was first dissolved in NaOH (0.1 M) and then diluted to the desired concentration (~ 10^–5^ M) with NaOH to give a solution of final pH = 12. The heamatin was reduced to [Fe(II)(PPXI)] with a slight excess of solid sodium dithionite. To prepare {Fe(II)(PPIX)(CO)_2_] the pH was raised to around 14 [174].

Electronic absorption Spectra used for verification of complexes in solution but not reported herein were obtained using a DU-7 spectrophotometer (Beckman).

Mössbauer spectra were recorded on concentrated frozen solutions at 78 K. The Mössbauer spectrometer and experimental details have previously been described [175].

## Data Availability

No datasets were generated or analysed during the current study.

## Abbreviations

Por: Porphyrin
L: A nitrogenous aliphatic, an aromatic base or a heterocyclic ligand
p.q.s.: Partial quadrupole splitting
p.c.s.: Partial chemical shifts
Fe(PPIX): Iron protoporphyrin IX
TPP: Tetra(phenyl)porphyrin
TMP: Tetramesitylporphyrin
OEP: Octaethylporphyrin
Hb: Haemoglobin
Mb: Myoglobin
*δ* (mm s^−1^): Mössbauer chemical shift
*Δ*EQ (mm s^−1^): Quadrupole splitting
pc: Phthalocyanine
TPPS: meso-tetrakis(p-sulfonatophenyl)porphyrin
TPPH_2_: α, β, γ, *δ*-tetraphenylporphin
py: Pyridine
pip: Piperidine
Im: Imidazole
1-VinylIm: Vinylimidazole
1-SiMe_3_Im: 1-(Trimethylsilyl)imidazole
1-BzylIm: 1-Benzylimidazole
1-AcIm: Acetylimidazole
1-MeIm: 1-Methylimidazole
but: N-Butylamine
^s^but: Secondary butylamine
Morph: Morpholine
Et_2_NH: Diethylamine
PrNH_2_: Propylamine
OHCH_2_CH_2_NH_2_: Ethanolamine
PMXPPH_2_: *α, β, γ, δ*-tetra(p-methoxyphenyl)porphin
TMXPPH_2_: *α, β, γ, δ*-tetra(2,4,6-methoxy-phenyl)porphin
PMEPPH_2_: *α, β, γ, δ*-tetra(p-tolyl)porphin
PClPPH_2_: *α, β, γ, δ*-tetra(p-chlorophenyl)porphin
OEPH_2_: Octaethylporphin
OMTBPH_2_: Octamethyltetrabenzoporphyrin
TMPH_2_: Tetramesitylporphin
TPPSH_2_: *α, β, γ, δ*-tetra(p-sulfophenyl)porphin
TNPSH_2_: *α, β, γ,δ*-tetra(p-sulfo 1-napthyl)porphin
(SP)_2_(SMeP)_2_PH_2_: *α, γ* Bis(p-sulfophenyl) *β, δ* bis(m-sulfo-p-tolyl)porphin
TNPH_2_: *α, β, γ, δ*-tetra 1-napthylporphin
TpivPPH_2_: “Picket Fence” porphyrin, meso-tetrakis(α,α,α,α-o-pivalamidophenyl)porphyrin
MbenTpivPPH_2_: Benzoyl modified “Picket Fence” porphin TImPPH2 = α,α,α,α-o-(1-methylimidazole-5-carboxylaminophenyl)porphin
TFPPBr_8_H_2_: Fully halogenated(on the vinyl rings *α, β, γ, δ*-tetra(pentafluorophenl)porphin
TFPPH_2_: *α, β, γ, δ*-tetra(pentafluorophenyl)porphin
Enim: Ethylenimine
Pydn: Pyrrolidine
DMM: 2,6-Dimethylmorpholine
THpy: Tetrahydropyridine
3-Mepip: 3-Methylpiperidine
4-NMe_2_py: 4-Dimethylaminopyridine
4-Acpy: 4-Acetylpyridine
3-Acpy: 3-Acetylpyridine
4-CNpy: 4-Cyanopyridine
4-Mepy: 4-Methylpyridine
3-CNpy: 3-Cyanopyridine
3,5-Me_2_py: 3,5-Dimethylpyridine
4-Mepip: 4-Methylpiperidine
3-Mepy: 3-Methylpyridine
2-MeHIm: 2-Methylimidazole
3-Clpy: 3-Chloropyridine
1,2-Me_2_Im: 1,2-Dimethylimidazole
2-Mepy: 2-Methylpyridine
3.4-Me_2_py: 3,4-Dimethylpyridine
3-MeNHpy: 3-Methylaminopyridine
4-NHTRIZ: 4-Amino 1,2,4 triazole
GEE: Glycine ethylester
1-EtIm: 1-Ethylimidazole
amp: 4-(2Aminoethyl)morpholine
Fe(C_5_H_5_)(C_4_H_4_N): Azaferrocene
PPIX: Protoporphyrin IX
MeNH_2_: Methylamine
EtNH_2_: Ethylamine
Et_2_NH: Di-ethylamine
(NH_2_CH_2_): 1,2-Diamino-ethane
oct: Octylamine
5-Cl,1-MeIm: 5-Chloro,1-methylimidazole
4-ClPy: 4-Chloropyridine
3-NHMepy: 3-Aminomethylpyridine
isoquin: Isoquinoline
CME: Cysteine methylester
His: Histidine
NAHis: N-α-acetyl histidine
2-Mepip: 2-Methylpiperidine
2-MeIm: 2-Methylimidazole
4(3H)pyr: 4-Hydroxypyrimidine
5-Mepyr: 5-Methylpyrimidine
2-Mepyra: 2-Methylpyrazine
2-Meopyra: 2-Methoxypyrazine
3-Mepyrida: 3-Methylpyridazine
THZ: Thiazole
OXZ: Oxazole
4-n-butTRIZ: 4-*N*-butyl 1,2,4 triazole
1-Cl2phTRIZ: 1-(2,4-Dichlorophenyl)-2-(1-*N*-1,2,4-triazole)-3-(hydroxy)-4-(4dimethyl)pentane
3.N(TTZMeac): 3-N(1,2,3,4 tetrazole)-1-methylacetate
2-Mepy: 2-Methylpyridine
^t^but: Tertiary butylamine
s.o.d.: Second-order doppler shift
TAAB: Tetraan-hydroaminobenzaldehyde
q_lattice_: Field gradient due to external charges
PPD: Protoporphyrin dimethyl ester
L’’: Sulfonated ligand tiv
EMCA: Ethyl 2-mercaptoacetate
CN^−^: Cyanide

## References

[1] Antonini E, Brunori M (1971) Hemoglobin and myoglobin in their reactions with ligands. North-Holland, Amsterdam

[2] Papathanasiou G, Mamali A, Papafloratos S, Zerva E (2014) Health Sci J 2014(8):272–288

[3] Turino GM (1981) Circulation (United States) 63:A253–A259

[4] Lemberg R, Barrett J (1971) Cytochromes. Academic Press, London

[5] Wilson MT (1993). In: Silver J (ed) Chemistry of iron. Blackie Academic and Professional, London

[6] Pasternak RF, Cobbs MA (1973) J Inorg Nucl Chem 35:4327–4339

[7] Fleischer EB, Jacobs S, Mestichelli L (1968) J Am Chem Soc 90:2527–2531

[8] Fleischer EB, Krishnamurthy M (1972) J Coord Chem 2:89–100

[9] Stynes DV, Stynes HC, Ibers JA, James BA (1973) J Am Chem Soc 95:1142–1149 4687683 10.1021/ja00785a024

[10] Brault D, Rougee M (1973) Nat New Biol 241:19 4512324 10.1038/newbio241019a0

[11] Brault D, Rougee M (1974) Biochemistry 13:4591–4597 4425650 10.1021/bi00719a019

[12] Coleman JP, Reed CA (1973) J Am Chem Soc 95:2048–2049 4689928 10.1021/ja00787a075

[13] Brault D, Rougee M (1974) Biochem Biophys Res Commun 57:654–659 4827828 10.1016/0006-291x(74)90596-8

[14] Hoard JL (1971) Science 174:1295–1302 4332625 10.1126/science.174.4016.1295

[15] Frauenfelder H, Sligar SG, Wolynes PG (1991) Science 254:1598–1603 1749933 10.1126/science.1749933

[16] Young RD, Frauenfelder H, Johnson JB, Lamb DC, Nienhaus GU, Phillip R, Scholl R (1991) Chem Phys 158:315–327

[17] Alben JO, Caughey WS (1968) Biochemistry 7:175–183 5758542 10.1021/bi00841a022

[18] Scheidt WR, Reed CA (1981) Chem Rev 81:543–555

[19] Scheidt WR, Gouterman M (1993). In: Lever ABP, Grey HB (eds) Iron porphyrins. Addison-Wesley, Reading

[20] Hu C, Noll BC, Schulz CE, Scheidt WR (2005) Inorg Chem 44:4346–4358 15934765 10.1021/ic050320p PMC1502394

[21] Lukas B, Miller JR, Silver J, Wilson MT (1982). J Chem Soc Dalton Trans. 10.1039/DT9820001035

[22] Lukas B, Silver J, Morrison IEG, Barnard PWC (1983) Inorg Chim Acta 78:205–210

[23] Lukas B, Silver J (1983) Inorg Chim Acta 78:219–224

[24] Lukas B, Silver J (1983) Inorg Chim Acta 80:107–113

[25] Lukas B, Peterson J, Silver J, Wilson MT (1983) Inorg Chim Acta 80:245–250

[26] Silver J, Lukas B, Al-Jaff G (1984) Inorg Chim Acta 91:125–128

[27] Silver J, Lukas B (1984) Inorg Chim Acta 91:279–283

[28] Silver J, Lukas B (1985) Inorg Chim Acta 106:219–222

[29] Silver J, Al-Jaff G, Taies JA (1987) Inorg Chim Acta 135:151–153

[30] Abu-Soud H, Silver J (1988) Inorg Chim Acta 152:61–66

[31] Medhi OK, Silver J (1988) Inorg Chim Aca 153:133–134

[32] Medhi OK, Houlton A, Silver J (1989) Inorg Chim 161:213–216

[33] Medhi OK, Silver J (1989). J Chem Soc Chem Commun. 10.1039/C39890001199

[34] Medhi OK, Silver J (1989) Inorg Chim Acta 164:231–234

[35] Medhi OK, Silver J (1990). J Chem Soc Dalton Trans. 10.1039/DT9900000263

[36] Medhi OK, Silver J (1990). J Chem Soc Dalton Trans. 10.1039/DT9900000555

[37] Medhi OK, Silver J (1990) Inorg Chim Acta 168:271–274

[38] Al-Jaff G, Silver J, Wilson MT (1990) Inorg Chim Acta 176:307–316

[39] Ahmet M, Al-Jaff G, Silver J, Wilson MT (1991) Inorg Chim Acta 183:43–49

[40] Silver J, Al-Jaff G, Wilson MT, den Engelsen D, Fern GR, Ireland TG (2022) J Biol Inorg Chem 27:297–313 35235042 10.1007/s00775-022-01929-4 PMC8960585

[41] Silver J, Al-Jaff G, Taies JA, Wilson MT, den Engelsen D, Fern GR, Ireland TG (2023) J Biol Inorg Chem 28:65–84 36478266 10.1007/s00775-022-01969-w PMC9938061

[42] Marsh PJ, Silver J, Symons MCR, Taiwo FA (1996). J Chem Soc Dalton Trans. 10.1039/DT9960002361

[43] Fern G, Silver J, Snowden MJ, Withnall R (1998) Proceedings of ICORS 16 Supplementary Volume Cape Town, South Africa p48

[44] Smalley JW, Silver J, Marsh PJ, Birss AJ (1998) Biochem J 331:681–685 9560292 10.1042/bj3310681 PMC1219405

[45] Cornelius VJ, Titler PJ, Fern GR, Miller JR, Silver J, Snowden MJ, McCammon CA (2002) Hyperfine Interact 144(135):359–363

[46] Cornelius VJ, Snowden MJ, Silver J, Fern GR (2004) React Funct Polym 58:165–173

[47] Peterson J, Silver J, Wilson MT, Morrison IEG (1980) J Inorg Biochem 13:75–82

[48] Peterson J, Saleem MMM, Silver J, Wilson MT, Morrison IEG (1983) J Inorg Biochem 19:165–178

[49] Adams PA, Milton RCL, Silver J (1984) Biometals 7:217–220 10.1007/BF00149551 8043986

[50] Withnall R, Silver J, Fern G, Smalley JW (1999) Proceedings of the 8th European Conference on the Spectroscopy of Biological Molecules Enschede, The Netherlands, p 573

[51] Smalley JW, Birss AJ, Silver J (2000) FEMS Microbiol Lett 183:159–164 10650220 10.1111/j.1574-6968.2000.tb08951.x

[52] Smalley JW, Withnall R, Birss AJ, Silver J (2000) J Dental Res 79:1187

[53] Smalley JW, Birss AJ, Withnall R, Silver J (2001) J Dental Res 80:1148

[54] Smalley JW, Birss AJ, Withnall R, Silver J (2002) Biochem J 362:239–335 11829761 10.1042/0264-6021:3620239 PMC1222381

[55] Smalley JW, Thomas MF, Birss AJ, Withnall R, Silver J (2004) Biochem J 379:833–840 14741050 10.1042/BJ20031221 PMC1224116

[56] Smalley JW, Charalabous P, Hart CA, Silver J (2003) Microbiology 149:843–853 12686627 10.1099/mic.0.26160-0

[57] Smalley JW, Silver J, Birss AJ, Withnall R, Titler PJ (2003) Microbiology 149:1711–1718 12855722 10.1099/mic.0.26258-0

[58] Adams PA, Berman PAM, Egan TJ, Marsh PJ, Silver J (1996) J Inorg Biochem 63:69–77 8699174 10.1016/0162-0134(95)00212-x

[59] Adams PA, Egan TJ, Rees DC, Silver J, Marsh PJ (1996) Biochem J 318:25–27 8761447 10.1042/bj3180025 PMC1217583

[60] Silver J, Lukas B (1984) Inorg Chim Acta 92:259–263

[61] Silver J, Lukas B, Taies JA (1987) Inorg Chim Acta 136:99–106

[62] Miller JR, Taies JA, Silver J (1987) Inorg Chim Acta 138:205–214

[63] Silver J, Taies JA (1988) Inorg Chim Acta 151:69–75

[64] Abu-Soud HM, Houlton A, Silver J (1988) Inorg Chim Acta 151:77–83

[65] Abu-Soud HM, Silver J (1988) Inorg Chim Acta 151:139–144

[66] Silver J, Taies JA (1988) Inorg Chim Acta 153:235–245

[67] Silver J, Taies JA (1989) Inorg Chim Acta 159:231–235

[68] Abu-Soud HM, Silver J (1989) Inorg Chim Acta 161:139–141

[69] Abu-Soud HM, Silver J (1989) Inorg Chim Acta 164:105–109

[70] Cesario M, Giannotti C, Guilhelm J, Silver J, Zakrewski J (1997). J Chem Soc Dalton Trans. 10.1039/a602366e

[71] Silver J, Marsh PJ, Symons MCR, Svistunenko DA, Frampton CS, Fern GR (2000) Inorg Chem 39:2874–2881 11232827 10.1021/ic990848s

[72] Berrett RR, Fitzsimmons BW (1967). J Chem Soc A. 10.1039/J19670000525

[73] Bancroft GM, Mays MJ, Prater BE (1970). J Chem Soc A. 10.1039/J19700000956

[74] Bancroft GM, Garrod REB, Maddock AG (1971). J Chem Soc A. 10.1039/j19710003165

[75] Bancroft GM, Libby ET (1973). J Chem Soc A. 10.1039/DT9730002103

[76] Silver J (1991) Inorg Chim Acta 184:235–242

[77] Dabrowiak JC, Merrell PH, Stone JA, Busch DH (1973) J Am Chem Soc 95:6613–6622

[78] Watkins DD, Riley DP, Stone JA, Busch EH (1976) Inorg Chem 15:387–393

[79] Nemykin VN, Polshina AE, Polshind EV, Kobayashi N (2000) Mendeleev Commun 10(2):54–55

[80] Nemykin VN, Polshina AE, Chernii VY, Polshind EV, Kobayashi N (2000). J Chem Soc Dalton Trans. 10.1039/b000096p

[81] Voloshin YZ, Polshin EV, Nazarenko AY (2002) Hyperfine Interact 141(142):1390–2320

[82] James BR, Sams JR, Tsin TB, Reimer KJ (1978). J Chem Soc Chem Commun. 10.1039/C39780000746

[83] Bancroft GM, Mays MJ, Prater BE (1968). Chem Commun. 10.1039/C19680001374

[84] Bancroft GM, Mays MJ, Prater BE (1969). J Chem Soc D Chem Commun. 10.1039/C29690000039

[85] Fitzsimmons BW, Seeley NJ, Smith AW (1969). J Chem Soc A. 10.1039/J19690000143

[86] Parish RV, Platt RH (1969). J Chem Soc A. 10.1039/J19690002145

[87] Clark MG (1969) Discuss Faraday Soc 47:144–148

[88] Bancroft GM, Mays MJ, Prater BE (1969) Discuss Faraday Soc 47:136–143

[89] McClure DC (1961). In: Kirshner S (ed) Advances in the chemistry of coordination compounds. MacMillan, New York, p 498

[90] Kobayashi H, Maeda Y, Yamagawa Y (1970) Bull Chem Soc Jpn 43:2342–2346

[91] Coleman JP, Hoard JL, Kim N, Lang G, Reed CAC (1975) J Am Chem Soc 97:2676–2681 166106 10.1021/ja00843a015

[92] Epstein LP, Straub DK, Maricondi C (1967) Inorg Chem 6:1720–1724

[93] Safo MK, Scheidt WR, Gupta GP (1990) Inorg Chem 29:626–633

[94] Dale BW, Williams RJP, Edwards PR, Johnson CE (1968) Trans Faraday Soc 64:620–629

[95] Straub DK, Connor WM (1973) Am N Y Acad Sci 206:383–396 10.1111/j.1749-6632.1973.tb43223.x 4518394

[96] Connor WM, Straub DK (1979) Inorg Chem 18:866–867

[97] Dolphin D, Sams JR, Tsin TB, Wong KL (1976) J Am Chem Soc 98:6970–6975 965659 10.1021/ja00438a037

[98] Polam JR, Wright JL, Christensen KA, Walker FA, Flint H, Winkler H, Grodzicki M, Trautwein AX (1996) J Am Chem Soc 118:5272–5276

[99] Sams JR, Tsin TB (1974) Chem Phys Lett 25:599–601

[100] Safo MK, Nesset MJM, Walker FA, Debrunner PG, Scheidt WR (1997) J Am Chem Soc 119:9438–9448

[101] Li J, Nair SM, Noll BC, Schulz CE, Scheidt WR (2008) Inorg Chem 47:3841–3850 18351735 10.1021/ic702498c PMC2543116

[102] Sams JR, Tsin TB (1979). In: Dolphin D (ed) The porphyrins, vol IV. Academic Press, New York, pp 425–478

[103] Calderazzo F, Pampaloni G, Vitali D, Pelizzi D, Collamoti I, Fredioni S, Serra AM (1980) J Organomet Chem 191:217–242

[104] He B, Schulz CE, Li J (2015) Dalton Trans 44:13651–13661 26145452 10.1039/c5dt00941c

[105] Yao Z, Schulz CE, Zhan N, Li J (2017) Inorg Chem 56:12615–12624 28980807 10.1021/acs.inorgchem.7b02092

[106] Hu B, He M, Yao Z, Schulz CE, Li J (2016) Inorg Chem 55:9632–9643 27676612 10.1021/acs.inorgchem.6b01364

[107] Ben Haj Hassen L, Ezzayani K, Rousselin Y, Stern C, Nasri H, Schulz CE (2016) J Mol Struct 1110:138–142

[108] Havlin RH, Godbout N, Salzmann R, Wojdelski M, Arnold W, Schulz CE, Oldfield E (1998) J Am Chem Soc 120:3144–3151

[109] Silvernail NJ, Roth A, Schulz CE, Noll BC, Scheidt WR (2005) J Am Chem Soc 127:14422–14433 16218637 10.1021/ja053148x PMC1866288

[110] Conner WM, Straub DK (1976) Inorg Chem 15:2289–2291

[111] Collman JP, Gagne RR, Reed CA, Halbert TR, Lang G, Robinson WT (1975) J Am Chem Soc 97:1427–1439 1133392 10.1021/ja00839a026

[112] Collman JP, Sorrell TN (1975) J Am Chem Soc 97:4133–4134 1159217 10.1021/ja00847a046

[113] Silvernail NJ, Noll BC, Schulz CE, Scheidt WR (2006) Inorg Chem 45:7050–7052 16933901 10.1021/ic0613356 PMC1586066

[114] Maeda Y, Harami T, Morita Y, Trautwein AX, Gonser U (1981) J Chem Phys 75:36–43

[115] Lang G, Marshall W (1966) Proc Phys Soc Lond 87:3–34

[116] Sharrock M, Munck E, Debrunner PG, Marshall V, Lipscomb JD, Gunsalus IC (1973) Biochemistry 12:278–265 10.1021/bi00726a013 4682999

[117] Reimer KJ, Sibley CA, Sams JR (1983) J Am Chem Soc 105:5147–5149

[118] Calderazzo F, Frediani S, James BR, Pampaloni G, Reimer KJ, Sams JR, Serra AM, Vitali D (1982) Inorg Chem 21:2302–2306

[119] Reed CA, Mashiko T, Scheidt WR, Spartalian K, Lang G (1980) J Am Chem Soc 102:2302–2306

[120] Radonovich LJ, Bloom A, Hoard JL (1972) J Am Chem Soc 94:2073–2078 4335729 10.1021/ja00761a046

[121] Zerner M, Gouterman M, Kobayashi H (1966) Theor Chim Acta 6:363–400

[122] Li J, Noll BC, Schulz CE, Scheidt WR (2009) Angew Chem Int 48:5010–5013 10.1002/anie.200901434 PMC2893548 19492380

[123] Debrunner P (1989). In: Lever APB, Gray HB (eds) Iron porphyrins part 3 chapter 2. VCH Publishers Inc, New York, pp 137–227

[124] Schappacher M, Ricard L, Weiss R, Monteil-Montoya R, Gonser U, Bill E, Trautwein A (1983) Inorg Chim Acta 78:L9–L12

[125] Schejter A, Plotkin B (1988) Biochem J 255:353–356 2848510 PMC1135229

[126] Frolov EN, Gvosdev R, Goldanskii VI, Parak FG (1997) JBIC 2:710–713

[127] Cooke R, Debrunner P (1968) J Chem Phys 48:4532–4537 5664904 10.1063/1.1668022

[128] Connor WM, Straub DK (1977) Inorg Chem 16:491–493

[129] Ohya T, Morohoshi H, Sato M (1983) Inorg Chem 23:1303–1304

[130] Tolman CA (1977) Chem Rev 77:313–348

[131] Grodzicki M, Flint H, Winkler H, Walker FA, Trautwein AX (1997) J Phys Chem A 101:4202–4207

[132] Yosikawa S, O’Keeffe DH, Caughey WS (1985) J Biol Chem 269:3518–3528 3972836

[133] Li J, Lord RI, Noll BC, Baik M-H, Schultz CE, Scheidt WR (2008) Angew Chem Int 47:10144–10146 10.1002/anie.200804116 PMC2855125 18989877

[134] Li J, Peng Q, Barabanschikov A, Pavlik JW, Alp EE, Sturhahn W, Zhao J, Sage JT, Scheidt WR (2012) Inorg Chem 51:11769–11778 23082814 10.1021/ic301719v PMC3498855

[135] Weiss JJ (1964) Nature (London) 202:83–84 14166723 10.1038/202083b0

[136] Pauling L (1964) Nature (London) 203:182–18314207238

[137] Li N, Petricek V, Coppens P, Landrum J (1985) Acta Cryst C41:902–905

[138] Li N, Coppens P, Landrum J (1988) Inorg Chem 27:482–488

[139] Peng SM, Ibers JA (1976) J Am Chem Soc 98:8032–8036 993515 10.1021/ja00441a025

[140] Salzmann R, Ziegler CJ, Godbout M, McMahon MT, Suslick KS, Oldfield E (1998) J Am Chem Soc 120:11323–11334

[141] Silvernail NJ, Noll BC, Scheidt WR (2005) Acta Cryst Sect E 61:1201–1203 10.1107/S1600536805015989 PMC2361390 18449359

[142] Cariati F, Morazzoni F, Zocchi M (1978) J Chem Soc Dalton Trans. 10.1039/DT9780001018

[143] Ricard L, Weiss R, Momenteau M (1986) J Chem Soc Chem Commun. 10.1039/c39860000818

[144] Salzmann R, McMahon MT, Godbout N, Sanders LK, Wojdelski M, Oldfield E (1999) J Am Chem Soc 121:3818–3828

[145] Collins DM, Scheidt WR, Hoard JL (1972) J Am Chem Soc 94:6689–6696

[146] Hoard JL (1973) Ann NY Acad Sci 206:18–31 4518386 10.1111/j.1749-6632.1973.tb43202.x

[147] Nasri H, Ellison MK, Shang M, Schulz CE, Scheidt WR (2004) Inorg Chem 43:2932–2942 15106981 10.1021/ic035119y PMC1764913

[148] Nasri H, Ellison MK, Krebs C, Huynh BH, Scheidt WR (2000) J Am Chem Soc 122:10795–10804

[149] Nasri H, Ellison MK, Chen S, Huynh BH, Scheidt WR (1977) J Am Chem Soc 119:6274–6283

[150] Scheidt WR, Piciulo PL (1976) J Am Chem Soc 98:1913–1919 1254850 10.1021/ja00423a044

[151] Wyllie GRA, Schulz CE, Scheidt WR (2003) Inorg Chem 42:5722–5734 12950223 10.1021/ic034473t PMC2080624

[152] Scheidt WR, Brinegar AC, Ferro EB, Kirner JF (1977) J Am Chem Soc 99:7315–7322

[153] Cao C, Dahal S, Shang M, Beatty AM, Hibbs W, Schulz CE, Scheidt WR (2003) Inorg Chem 42:5202–5210 12924891 10.1021/ic030043r PMC1993896

[154] Martins LMDRS, Duarte MT, Galvão AM, Resende C, Pombeiro AJL, Henderson RA*,* Evans DJ (1998) J Chem Soc Dalton Trans 3311–3317

[155] Shenoy GK (1984). In: Long GJ (ed) Mössbauer spectroscopy applied to inorganic chemistry. Plenum Press, New York, pp 57–76

[156] Bancroft GM, Mays MJ, Prater BE, Stefanini FP (1970) J Chem Soc A 2146–2149

[157] Fajans K (1923) Naturvissenschaften 11:165–172

[158] Tsuchida R (1938) Bull Chem Soc Jpn 13:388–400

[159] Tsuchida R (1938) Bull Chem Soc Jpn 13:434–450

[160] Tsuchida R, Kobayashi M (1938) Bull Chem Soc Jpn 13:471–480

[161] Jorgensen CK (1962) Absorption Spectra and Chemical Bonding in Complexes. Pergamon Press, Oxford, pp 107–133 (**Chapter 7**)

[162] Shimura Y (1988) Bull Chem Soc Jpn 61:693–698

[163] Riley DP, Stone JA, Busch DH (1976) J Am Chem Soc 98:1752–1762

[164] Pilsbury DG, Busch DH (1976) J Am Chem Soc 98:7836–7839 993500 10.1021/ja00440a072

[165] Thon VJ, Fox CC, Boeyens JCA, Hancock RD (1984) J Am Chem Soc 106:5947–5955

[166] Riley DP, Stone JA, Busch DH (1977) J Am Chem Soc 99:767–777

[167] Koch S, Holm RH, Frankel RB (1975) J Am Chem Soc 97:6714–6723 10.1021/ja00837a052 1133375

[168] Bren KL, Eisenberg R, Gray HB (2015) PNAS 112:13123–13127 26508205 10.1073/pnas.1515704112 PMC4629386

[169] Adler AD, Longo FR, Finarelli JD, Goldmacher J, Assour J, Korsakoff L (1967) J Org Chem 32:467

[170] Torrens MA, Straub DK, Epstein LM (1972) J Am Chem Soc 94:4160–4162 5036644 10.1021/ja00767a018

[171] Fleisher EB, Palmer JM, Srivastava TS, Chatterjee A (1971) J Am Chem Soc 93:3162–3167 5559598 10.1021/ja00742a012

[172] Unpublished results Silver J, Taies JA (1987) (Taies JA PhD Thesis University of Essex).

[173] Taies JA (2012) J Univ Anbar Pure Sci 6:1–7

[174] Lukas B. Silver J, Wilson MT, Harmesh A, Bicker D to be submitted

[175] Hamed MY, Hider RC, Silver J (1982) Inorg Chim Acta 66:13–18

